# Disruption of *NEUROD2* causes a neurodevelopmental syndrome with autistic features via cell-autonomous defects in forebrain glutamatergic neurons

**DOI:** 10.1038/s41380-021-01179-x

**Published:** 2021-06-29

**Authors:** Karen Runge, Rémi Mathieu, Stéphane Bugeon, Sahra Lafi, Corinne Beurrier, Surajit Sahu, Fabienne Schaller, Arthur Loubat, Leonard Herault, Stéphane Gaillard, Emilie Pallesi-Pocachard, Aurélie Montheil, Andreas Bosio, Jill A. Rosenfeld, Eva Hudson, Kristin Lindstrom, Saadet Mercimek-Andrews, Lauren Jeffries, Arie van Haeringen, Olivier Vanakker, Audrey Van Hecke, Dina Amrom, Sebastien Küry, Chana Ratner, Reena Jethva, Candace Gamble, Bernard Jacq, Laurent Fasano, Gabriel Santpere, Belen Lorente-Galdos, Nenad Sestan, Antoinette Gelot, Sylvie Giacuzz, Sandra Goebbels, Alfonso Represa, Carlos Cardoso, Harold Cremer, Antoine de Chevigny

**Affiliations:** 1 INMED, INSERM, Aix-Marseille University, Marseille, France; 2 IBDM, Aix-Marseille University, CNRS, UMR, Marseille, France; 3 TAGC INSERM U1090, Aix-Marseille University, Marseille, France; 4 Phenotype Expertise, 5 Boulevard du Maréchal Koenig, Marseille, France; 5 Miltenyi Biotec, Bergisch-Gladbach, Germany; 6 Baylor College of Medicine, Houston, TX USA; 7 Cook Children’s Clinical Genetics, Fort Worth, TX USA; 8 Division of genetics and Metabolism, Phoenix Children’s Hospital, Phoenix, AZ USA; 9 Department of Medical Genetics, Faculty of Medicine & Dentistry, University of Alberta, Edmonton, AB Canada; 10 Pediatric Genomics Discovery Program, Department of Pediatrics, Yale University School of Medicine, New Haven, CT USA; 11 Department of Clinical Genetics, Leiden University Medical Center, Leiden, Netherlands; 12 Center for Medical Genetics, Department of Biomolecular Medicine, Ghent University and Ghent University Hospital, Ghent, Belgium; 13 Department of Neurology, Queen Fabiola Children’s University Hospital, Brussels, Belgium; 14 Université Libre de Bruxelles (ULB), Brussels, Belgium; 15 Neuropediatric Unit, Kannerklinik, Centre Hospitalier de Luxembourg, Luxembourg, Grand-Duchy of Luxembourg, Luxembourg, Luxembourg; 16 Centre Hospitalier Universitaire de Nantes, Service de Génétique Médicale, Nantes, France; 17 INSERM, CNRS, UNIV Nantes, l’institut du thorax, Nantes, France; 18 Hackensack University Medical Center, Hackensack, NJ USA; 19 Department of Neuroscience and Kavli Institute for Neuroscience, Yale School of Medicine, New Haven, CT USA; 20 Trousseau Hospital, Paris, France; 21 Max-Planck-Institute of Experimental Medicine, Goettingen, Germany

**Keywords:** Neuroscience, Autism spectrum disorders

## Abstract

While the transcription factor NEUROD2 has recently been associated with epilepsy, its precise role during nervous system development remains unclear. Using a multi-scale approach, we set out to understand how *Neurod2* deletion affects the development of the cerebral cortex in mice. In *Neurod2* KO embryos, cortical projection neurons over-migrated, thereby altering the final size and position of layers. In juvenile and adults, spine density and turnover were dysregulated in apical but not basal compartments in layer 5 neurons. Patch-clamp recordings in layer 5 neurons of juvenile mice revealed increased intrinsic excitability. Bulk RNA sequencing showed dysregulated expression of many genes associated with neuronal excitability and synaptic function, whose human orthologs were strongly associated with autism spectrum disorders (ASD). At the behavior level, *Neurod2* KO mice displayed social interaction deficits, stereotypies, hyperactivity, and occasionally spontaneous seizures. Mice heterozygous for *Neurod2* had similar defects, indicating that *Neurod2* is haploinsufficient. Finally, specific deletion of *Neurod2* in forebrain excitatory neurons recapitulated cellular and behavioral phenotypes found in constitutive KO mice, ﻿revealing the region-specific contribution of dysfunctional *Neurod2* in symptoms. Informed by these neurobehavioral features in mouse mutants, we identified eleven patients from eight families with a neurodevelopmental disorder including intellectual disability and ASD associated with *NEUROD2* pathogenic mutations. Our findings demonstrate crucial roles for *Neurod2* in neocortical development, whose alterations can cause neurodevelopmental disorders including intellectual disability and ASD.

## Introduction

Alterations in cellular migration, synaptic transmission, and intrinsic excitability of cortical projection neurons (cPNs) are prevalent theories of the pathophysiology of neurodevelopmental disorders (NDDs) including autism spectrum disorders (ASD) and intellectual disability [1–3]. Despite a field of intense investigation, the mechanisms and genes regulating each of these processes remain poorly known.

NEUROD2 belongs to the family of NEUROD basic helix-loop-helix transcription factors that regulate early neuronal differentiation during development [4]. While cortical expression of its closest and first identified paralog NEUROD1 is turned off around birth [4], NEUROD2 cortical expression persists postnatally [5], indicating that it might be involved in maturational processes. Furthermore, few studies have suggested a link between NEUROD2 and synapse formation. *Neurod2* mutant mice show reduced density of synaptic markers in the amygdala [6] and of dendritic spines in projection neurons of selected hippocampal subfields [7]. Moreover, the degradation of NEUROD2 by the ubiquitin-proteasome system is required for the maturation of presynaptic terminals in cerebellar neurons [8]. Also, the electrophysiological maturation of the thalamo-cortical synapse is altered in the barrel cortex of *Neurod2* deficient mice [9].

Interestingly, two recent studies suggest that NEUROD2 might be associated with NDDs. Indeed, two children with epilepsy and one child with neurodevelopmental delay were identified with rare *de novo* mutations in the DNA binding domain of NEUROD2, and functional testing in tadpoles suggested pathogenicity [10, 11]. Although a causative role for NEUROD2 in epilepsy is validated by the phenotypic overlap in two epileptic patients with similar mutations, a single patient with neurodevelopmental delay is currently insufficient to determine whether NEUROD2 is involved in NDDs.

With hippocampus and amygdala, the cerebral cortex is the brain structure that is the most strongly associated with NDDs [12, 13]. Current knowledge about the role of *Neurod2* in cortex development is limited. *Neurod2* deficient mice have a morphologically disorganized barrel cortex although layer 4 (L4) cPNs are present [9]. Electrophysiologically, L2/3 cPNs show increased intrinsic excitability in the somatosensory cortex [14]. In spite of this information, the impact of *Neurod2* deletion on the different steps of cortical development has not been thoroughly investigated.

Here, by combining mouse genetics, *in utero* electroporation, neuronal tracing, reconstruction, histology, electrophysiology, RNA-seq, behavioral testing, and *in silico* quantitative analyses, we provide strong experimental evidence for a causal relationship between *Neurod2* disruption, morpho-functional defects in cPNs and ASD-like behavioral symptoms in mice. Informed by these neurobehavioral features in mouse mutants, we searched for and identified five families with pathogenic *NEUROD2* mutations associated with intellectual disability, ASD, hyperactivity, and speech delay, with or without epilepsy. These features overlap with those uncovered in the mouse studies, demonstrating the necessity of NEUROD2 for normal brain development and revealing the region-specific contributions of dysfunctional NEUROD2 to intellectual disability, ASD, hyperactivity, and social-behavioral deficits.

## Results

### NEUROD2 is restricted to cPNs in the embryonic and postnatal mouse neocortex

**Fig. 1:**
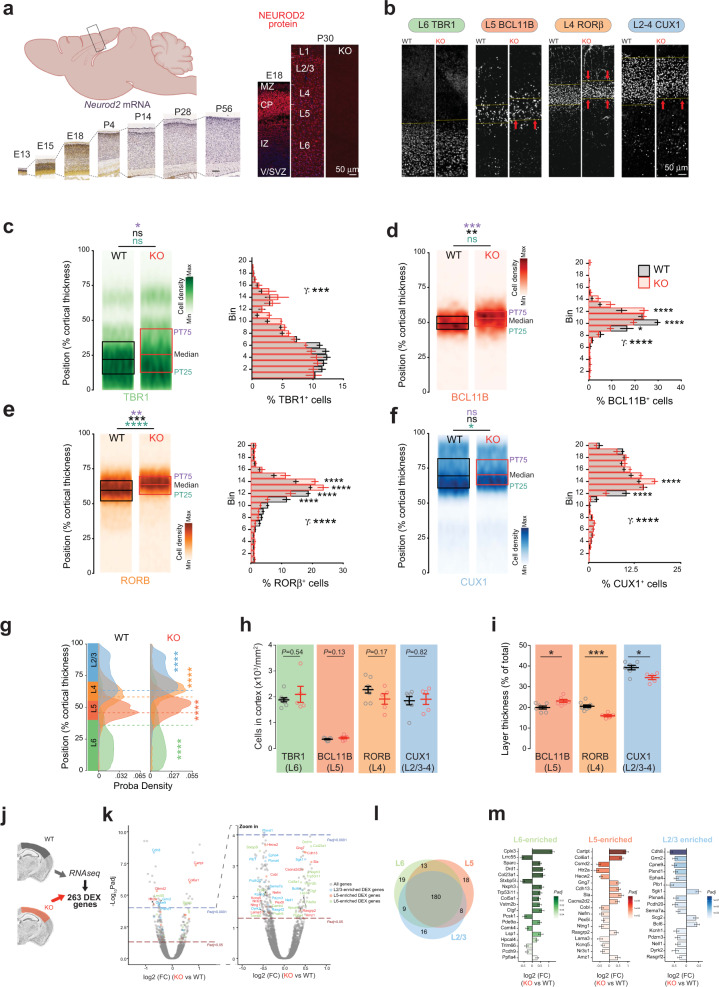
Altered laminar organization and gene expression in the cortex of *Neurod2* null mice. **a**–**i** Altered lamination of cortical layers at P30 in *Neurod2* KO mice. **a** Neurod2 mRNA and NEUROD2 protein were expressed from corticogenesis to adulthood. In situ hybridization pictures are from ALLEN BRAIN ATLAS. CP: cortical plate, IZ: intermediate zone, MZ: marginal zone, V/SVZ: ventricular/subventricular zone. **b** Representative confocal photomicrographs of S1 cortical columns at the somatosensory level for the four markers analyzed. **c**–**f** Left, mean cell laminar position in percentage of the cortical thickness. Right, twenty-bin based laminar distribution of cPN subtypes in WT (black bars) and KO mice (red bars). We used the markers TBR1 for L6 (**c**), BCL11B for L5 (**d**), RORB for L4 (**e**) and CUX1 for L2-4 (**f**). We analyzed 25 to 42 S1 cortical columns from 9/6/9/7 WT and 5/5/5/5 KO mice (for TBR1, BCL11B, RORB and CUX1, respectively). **g** Probability density function showing the average laminar distribution of cells. All layers were switched superficially, with the strongest significance for L5 and L4. **h**–**k** Dysregulation of layer-specific genes in *Neurod2* KO mice. (**h**) Number of cPN subtypes in a S1 cortical column, labeled using the four-layer markers; note the absence of significant difference between genotypes. **i** Thickness of the BCL11B, RORB, and CUX1 layers. **j**–**m** Differential expression in *Neurod2* KO S1-M1 cortex at P30. **j** Schematic of the RNA-seq experiment. **k** Volcano plots depicting differential gene expression. Right plot is a magnification of left plot (*N* = 3 biological replicates). **l** Venn diagram of DEX genes showing enrichement in L2/3, L5, and L6 (180 are generic to all layers, some are common between 2 layers and others specific to a single layer). **m** Fold change expression (FC; log2 scale) of DEX genes showing layer enrichment in normal conditions. Left bars depict downregulated genes, right bars show upregulated genes. Genes are ranked according to adjusted *P* value). Data are represented as means ± SEM except in mean cell position plots (**c**–**f**) where median, first and third quartiles are represented. Statistical significance was evaluated by permutation test for medians [(**c**–**f**), (**h**–**i**)], Student *t*-test or Mann–Whitney test depending on normality of samples [(**h**–**i**)], Anderson–Darling test [probability densities in (**g**)] or by Permutation test for a spatially adjusted two-way ANOVA followed by Bonferroni’s post hoc test [bin graphs in (**c**–**f**)] (*P < 0.05, **P < 0.01, ***P < 0.001, ****P < 0.0001). See also Figs. S1–S3.

To characterize the temporal expression of *Neurod2* in the neocortex, we first performed quantitative real-time PCR from the whole cortex of embryonic and postnatal stages. *Neurod2* mRNA expression increased from E14.5 to reach a peak at E18.5, and remained at lower albeit constant levels postnatally (Fig. S1a). In situ hybridizations from ALLEN BRAIN ATLAS (http://www.brain-map.org/) revealed *Neurod2* mRNA expression in the cortical plate (CP) and hippocampus (not shown) starting from E13.5 onwards (Fig. 1a). In the postnatal cortex, *Neurod2* mRNA was maintained after birth, and observed in L2 to L6 at all ages examined.

At the protein level, NEUROD2 was also detected embryonically, in the intermediate zone (IZ) where migration occurs and in the CP but not in the germinal ventricular/subventricular zone (V-SVZ, Fig. 1a), confirming previous results [15]. NEUROD2 protein was maintained postnatally in cortical L2 to L6 (Fig. 1a). We immunolabeled brain slices with cell type markers to identify which cells express NEUROD2. It co-localized with the L5 pyramidal tract cPN marker BCL11B (Fig. S1b) and with cPN markers of other layers (TBR1 for L6, RORB for L4 and SATB2, and CUX1 for L2/3) at all ages examined (P3, P30, P90 and 1 year, not shown). In contrast, NEUROD2 was never detected in Gad67-GFP^+^ inhibitory neurons (Fig. S1c), nor in non-neuronal cortical cells (not shown). In sum, *Neurod2* mRNA and NEUROD2 protein are expressed in migratory and post-migratory cPNs of L2 to L6.

### Gross anatomy and axonal output targets of the cortex are preserved in *Neurod2* KO mice

We examined the cortical anatomy of *Neurod2* KO mice at P30, when cPNs shall have completed the migration and are fully integrated into the cortical circuit. The thickness of the corpus callosum was slightly reduced (Fig. S2a), while cortical anatomy appeared otherwise grossly normal as indicated by the normal aspect and thickness of the cortical wall at sensory-motor levels (Fig. S2b). However, the cortex area from coronal sections that included M1 and S1 was slightly reduced in *Neurod2* KO mice (Fig. S2b), suggesting subtle alterations in developmental cell death and/or proliferation at least in some cortical areas. Cell proliferation in germinative zones was normal in *Neurod2* KO mice at E18, P7 and P30, as demonstrated by KI67 and PH3 immunostainings (Fig. S3). This result was consistent with the absence of NEUROD2 expression in VZ progenitors. Quantification of activated caspase-3 positive cells to evaluate apoptosis in all brain areas did not show any defect at E18, P7, and P30, suggesting that subtle changes in cell death, if any, were too subtle to be visualized with current methods.

We assessed the axonal outputs of cortical layers in M1 by injecting retrograde cholera toxin beta (CTB)-based tracers in the typical output target areas. In both WT and *Neurod2* KO mice, cPNs with thalamic and striatal projections settled in L6 and L5, respectively (Fig. S2d), while cPNs projecting to contralateral M1 were distributed in layers L2-6 with strong enrichment in L2/3 (Fig. S2d). Thus, gross anatomical organization and axonal targeting specificities of cPN subtypes are preserved in *Neurod2* KO mice.

### Laminar position of cortical layers is shifted superficially in *Neurod2* KO mice

We quantified numbers and laminar distribution of cPN subtypes in S1 at P30. We used immunostainings for major regulatory transcription factors of selected cortical layers, TBR1 for L6, BCL11B for L5 pyramidal tract, RORβ for L4, and CUX1 for L2-4 cPNs. Each cPN type was found in normal amounts in the cortex (Fig. 1b, h). This finding is coherent with unaltered proliferation and apoptosis in *Neurod2* KO mice (Fig. S3). Global inside-out cortical patterning was also normal since TBR1, BCL11B, RORB, and CUX1 were successively expressed from deep to superficial layers (Fig. 1b). Strikingly however, cortical layers were shifted towards the pial surface in *Neurod2* KO mice (Fig. 1c–g). More specifically, TBR1 and BCL11B-expressing deep layers extended superficially (Fig. 1c, d, g). This superficial extension of deep layers was associated with an enlargement of L5 (Fig. 1i) and a thinning of RORβ and CUX1-expressing layers 2–4, which were shifted superficially (Fig. 1i).

### *Neurod2* deletion alters gene expression in the neocortex

To identify genes that are regulated by the transcription factor *Neurod2* in the neocortex, we performed RNA-seq on motor and somatosensory cortices isolated from *Neurod2* WT versus KO mice at P30. Comparative analysis identified 263 differentially expressed (DEX) genes, among which 185 were downregulated and 78 were upregulated in KO mice (Fig. 1j, k; File S1 for a complete list). To determine whether some of the DEX genes are enriched in specific layers and might have functional roles in their development, we integrated information from the literature and several public data sources (DeCoN, Allen Brain Atlas, Single Cell Analysis of Mouse Cortex, GenePaint, Eurexpress). This search yielded 180 genes generic to all layers and 83 genes which showed layer enrichment (Fig. 1l, m). The proportion of layer-specific factors among DEX genes was similar in L2/3, L5, and L6 (Fig. 1l, m), consistent with the idea that *Neurod2* impacts cPNs from all layers.

### Radial over-migration of cPNs in *Neurod2* KO and heterozygous (HET) mice

**Fig. 2:**
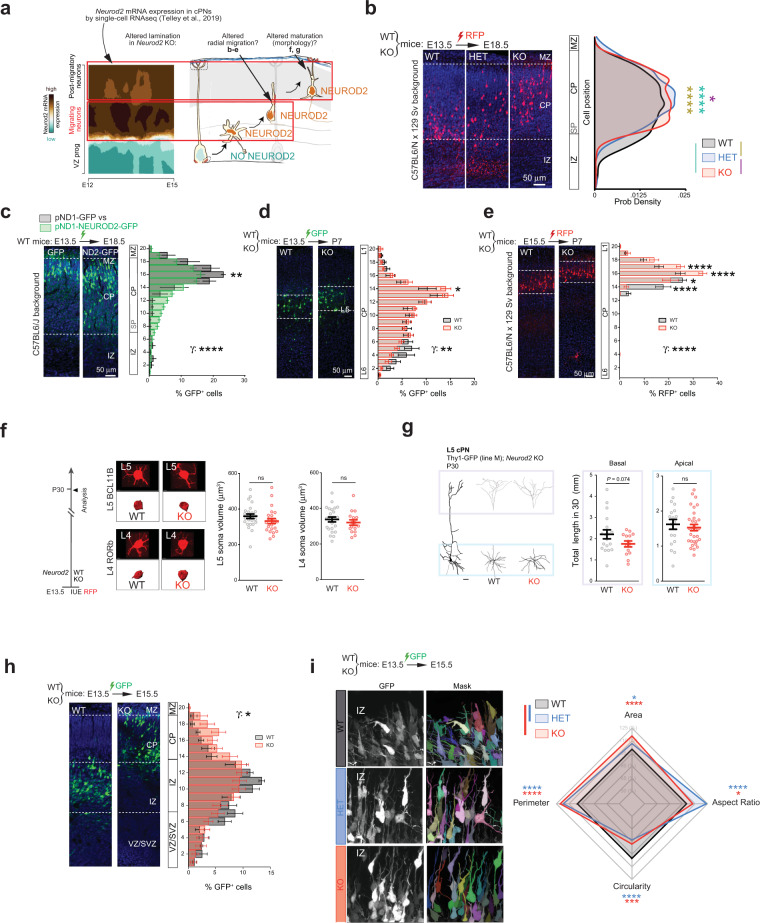
Radial over-migration of cPNs in *Neurod2* KO and HET mice. **a** Spatio-temporal expression of *Neurod2* from a longitudinal scRNA-seq study [16]. X axis is time of apical progenitor birth, Y axis represents time of neuron differentiation (1 h for VZ progenitors, 24 h for migrating neurons, 48 h for post-migratory neurons). The strongest expression of *Neurod2* mRNA is in migrating neurons. **b** Excess migration in *Neurod2* KO and HET mice. The probability density function is represented. See Fig. S4b for additional cell position analyses, which corroborate the over-migration phenotype. We analyzed 21 slices from 4 WT, 20 slices from 5 HET and 26 slices from 3 KO mice. **c** Post-mitotic overexpression of *NEUROD2* of WT mice reduced the distance migrated by cPNs. We analyzed 21 slices from 3 pND1-GFP mice and 65 slices from 10 pND1-NEUROD2-GFP mice. **d** Over-migration was maintained post-migration, at P7. We analyzed 9 slices from 2 WT and 13 slices from 3 KO mice. **e** Over-migration of L2/3 cPNs born at E15.5 was also evident. We analyzed 26 slices from 5 WT and 15 slices from 3 KO mice. **f**, **g** At P30, somatic volume (**f**) and dendritic complexity (**g**) from 3D-reconstructed neurons were not significantly altered. For somatic volumes we analyzed 24/26 L4/L5 cells from 3 WT mice and 16/24 L4/L5 cells from 3 KO mice (individual cells plotted). For the dendritic length we measured 17/19 basal/apical dendrites from 4 WT and 14/28 basal/apical dendrites from 4 KO mice (circles represent individual dendrites). **h** Over-migration was already visible at 2 days post-electroporation in KO mice. We analyzed 6 slices from 2 WT and 9 slices from 3 KO mice. **i** Radar plot investigating morphological parameters. HET and KO cells showed decreased circularity, increased aspect ratio, increased perimeter, and increased area. 704 WT, 659 HET, and 410 KO cells were investigated from 6, 6, and 5 mice, respectively. Data are represented as means ± SEM. Statistical significance was evaluated by Anderson-Darling test [probability density graph in (**b**)], permutation test for a spatially adjusted two-way Anova followed by post-hoc analysis with Bonferroni correction [bin graphs in (**c**), (**d**), (**e**), (**h**)], Student *t*-test or Mann–Whitney test depending on normality of samples [morphometric analyses in (**f**), (**g**)], and by a two-sided permutation *t*-test [radar plot in (*i*), see also Fig. S4-S5]. (*P < 0.05, **P < 0.01, ***P < 0.001, ****P < 0.0001). See also Figs. S4, S5, and S6.

Superficially-shifted layers in *Neurod2*-null mice could result from altered radial migration or from post-migratory maturational defects such as changes in somatic/dendritic sizes of cPNs (Fig. 2a, right). Interestingly, searching for *Neurod2* in a recent single-cell analysis of temporal patterning during corticogenesis shows that *Neurod2* expression reaches a peak in radially migrating cPNs [16] (Fig. 2a, left). Furthermore, our own gene ontology analysis of NEUROD2-bound genes at E14.5 cortex from a published study [17], a time during which radial migration occurs, revealed 136 migration-related bound genes including 20 radial cortical migration genes (Fig. S4a, File S1 Sheet ‘egoE14.5_Chip_Seq_neuronMigrati_Disc’). These data indicate that NEUROD2 might be important for the transcriptional control of radial migration.

First, we asked whether the lamination phenotype observed at P30 might be present already at the end of the layer formation period, i.e., at P7, which would be consistent with an alteration of cPN radial migration. We labeled P7 brain sections for BCL11B, RORB, and CUX1 and quantified cell type numbers and distributions in S1. cPN subtypes expressing BCL11B, RORB, and CUX1 showed normal numbers (Fig. S5a, b) but superficial shifts in their average laminar positions (Fig. S5c–f), reminiscent of the P30 lamination phenotype. This suggests that early events such as excessive radial migration might provoke the phenotype.

Then, we directly assessed if radial migration was altered in *Neurod2* KO mice during embryogenesis. We tracked a cohort of L5 cPNs by performing *in utero* electroporation of a red fluorescent plasmid at E13.5 and analyzed cell distributions at E18.5, which is around the end of their radial migration. Multiple types of cell distribution analyses including probability density (Fig. 2b), cell laminar position, cumulative distribution, and bin-based laminar distribution (Fig. S4b) showed that at E18.5 the migrating neurons had reached more superficial layers in *Neurod2* KO mice than in control littermates, revealing an over-migration phenotype. Importantly, heterozygous (HET) littermates displayed a phenotype that was similar to the KO mice (Fig. 2b and Fig. S4b), demonstrating that cortical migration is sensitive to *Neurod2* haploinsufficiency. At the opposite of the HET and KO phenotypes, adding extra copies of *NEUROD2* in post-mitotic cPNs of control mice using the post-mitotic *Neurod1* promoter increased the fraction of cells in deep layers as compared to a control plasmid (Fig. 2c, S4c). Is this over-migration maintained postnatally? We sacrificed another group of mice at P7, after the end of migration. In *Neurod2* KO mice, electroporated cell still lied more dorsally than in controls (Fig. 2d) which confirmed the maintenance of the migratory phenotype. To test whether *Neurod2* is important for radial migration of other cPNs, we labeled L2/3 cPNs by in utero electroporation at E15.5. Like for L5 neurons, the labeled cohort was shifted more dorsally at P7 in the *Neurod2* KO genotype (Fig. 2e), suggesting that pro-migratory effect of *Neurod2* deletion can be generalized to cPN subtypes.

Together, our *in utero* electroporation experiments demonstrate that *Neurod2* regulates the radial migration of cPNs, and the fact that a focal mosaic expression of extra *NEUROD2* copies in a wild-type mouse is sufficient to hamper migration suggests that this role is cell autonomous.

Post-migratory alterations in cell or neuropil volumes could also contribute to the distal shift in cPN lamination at P30. However, somatic volumes (Fig. 2f) of L4 and L5 cPNs and dendritic complexity of L5 cPNs (Fig. 2g) were not significantly altered. Thus, the abnormal cortical lamination in *Neurod2* KO mice is a consequence of embryonic radial migration defects rather than post-migratory events such as variations in neuronal size.

We next searched for the possible cellular mechanisms by which *Neurod2* deficiency and haploinsufficiency provoke cPN over-migration. Cortical radial migration occurs in three consecutive steps: multipolar-bipolar transition from slow to fast migration in the IZ, glia-guided locomotion in the CP, and terminal somal translocation in the primitive cortical zone (PCZ), the uppermost part of the CP, in response to cues secreted by the marginal zone (MZ). A recent study described that RNA interference against *Neurod2* in wild type mice induced “ectopic” neurons in the MZ (corresponding to bins 19-20 in Fig. 2b–e), compensated by reduced neuronal numbers in the PCZ (bins 17-18 in Fig. 2b–e) [18]. This suggested a link between *Neurod2* interference and terminal somal translocation. We did not observe this phenotype in *Neurod2* KO and HET mice as the percentage of cells in MZ was not affected despite a similar electroporation paradigm (Fig. S4d, e). Accordingly, thicknesses of MZ and subsequent L1 were unaltered in HET and KO brains at all ages examined (E18, P7, and P30) (Fig. S4f). Instead, the IZ and lower CP showed the strongest reductions in electroporated cell proportions in E13-E18 electroporated brains (Fig. 2b; Fig. S4b, bin graph), indicating that over-migration arose during IZ/CP crossing, likely through facilitation of the transition from slow multipolar to fast glia-guided migration. In line with this hypothesis, over-migration was evident already at 2 days post-electroporation when cells had not started terminal translocation yet (Fig. 2h), confirming that alterations of cell behavior in IZ and/or lower CP were instead responsible for over-migration. Although the average number of thin neurites in IZ multipolar neurons seem unaffected in KO and HET mice (Fig. S4g), an unbiased analysis of cellular shape using state-of-the-art deep learning algorithms (see “Materials and methods”) highlighted that more cells had elongated morphologies and increased overall areas and perimeters, typical of the multipolar-to-bipolar transition (Fig. 2h, Fig. S6). Elongation was measured by circularity and aspect ratio (long axis length/short axis length) (Fig. 2h, Fig. S6). In sum, cPNs display favored radial migration in the IZ and lower CP of Neurod2 HET and KO mice, at least in part through a favored multipolar to bipolar transition.

### Altered excitatory synapse density and turnover in Neurod2 KO mice

**Fig. 3:**
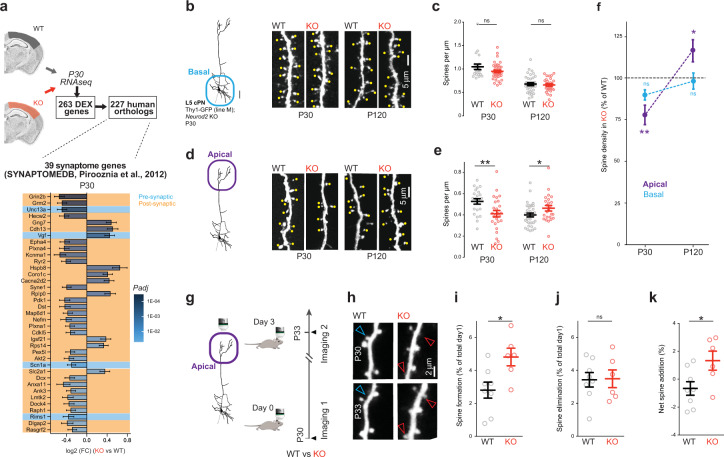
Altered excitatory synapse density and turnover in *Neurod2* KO mice. **a** Among the 227 human orthologs of the 263 DEX genes at P30, 39 were synaptome genes, including 4 pre-synaptic and 35 post-synaptic. This suggests synaptic alterations in *Neurod2* KO mice. Bottom: fold change of DEX gene expression (log2 scale), genes are ranked according to *P*adj. In blue, pre-synaptic genes; in orange, post-synaptic genes. (**b–f**) Age-dependent spine density defects in L5 cPNs. **b**–**c** Spine density in basal dendrites. **b** Representative photomicrographs of GFP-expressing basal dendritic stretches with spines underscored by yellow dots. **c** Spine density at P30 and P120 in the basal compartment. We counted 21 dendritic segments from 4 WT and 40 segments from 6 KO mice at P30, 33 segments from 4 WT and 27 segments from 4 KO at P120 (individual cells plotted). **d**, **e** Apical tuft spine density. Representative images (**d**) and apical spine density (**e**). **f** Spine density variation relative to WT in basal and apical compartments. We analyzed 22 dendritic segments from 5 WT and 26 segments from 7 KO mice at P30, 34 segments from 4 WT and 27 segments from 4 KO at P120 (individual cells plotted). **g**–**k** Increased spine turnover in an apical tuft at P30. **g** Scheme of the experimental paradigm. **h** Representative 2-photon images of same dendrites at 3 days intervals. Red and blue arrowheads depict gained and lost spines, respectively. **i** Spine formation, **j** spine elimination and **k** net spine addition over the 3-day interval period. We counted spine changes in 42 dendritic segments from 8 WT mice and 39 segments from 6 KO mice between P30 and P33 (animals plotted). Data are represented as means ± SEM. Statistical analyses were performed using two-tailed *t*-tests or Mann–Whitney test depending on the normality of samples. *P < 0.05, **P < 0.01. See also Fig. S7.

Maintenance of NEUROD2 expression in post-migratory maturing and mature cPNs (Fig. 1) suggests that it might also play a role on synaptogenesis, synaptic transmission, and/or synaptic plasticity [7]. This is corroborated by our RNAseq at P30, which shows that among the 227 human orthologs of *Neurod2* KO DEX genes, 39 are associated with the synaptome (4 with the presynapse, 25 with the post-synapse) (Fig. 3a). The Thy1-GFP mouse allowed us to gain experimental access to the fine morphology of dendritic spines [19], the post-synaptic elements of excitatory synapses, in L5 cPNs of *Neurod2* KO mouse M1. We measured spine density on basal and apical compartments at two ages, P30 and P120, which correspond to juvenile and adult ages, respectively. In basal dendrites, spine density was unchanged at both P30 and P120 (Fig. 3b, c). In both WT and *Neurod2* KO mice there was a significant reduction in basal spine density between P30 and P120, consistent with synaptic pruning (Fig. S7a, b). In contrast, apical tuft dendrites showed abnormal spine density in *Neurod2* KO mice. Surprisingly, spine density was decreased at P30 but increased at P120 when compared to WT littermates (Fig. 3d–f and Fig. S7c). The density of inhibitory synapses received by L5 neurons, assessed by counting Gephyrin-GFP^+^ puncta at P30 after Gephyrin-GFP intrabody electroporation at E13.5 [20], was normal in both basal and apical compartments of *Neurod2* KO mice (Fig. S7d, e).

The age-dependent difference in spine density defects in the apical tuft of *Neurod2* mutant mice suggested that spine turnover rates in this compartment might be abnormal in the temporal window examined. We used 2-photon live imaging to measure spine turnover at the beginning of the temporal window, i.e., P30. We counted the proportion of formed and eliminated spines on identified dendritic segments at 3 days interval, between P30 and P33 (Fig. 3g). Formed and eliminated spines compensated for each other in WT mice (Fig. 3h–k). In contrast, formed spines outnumbered eliminated spines in *Neurod2* KO mice, resulting in a net spine gain (Fig. 3h–k). In sum, net spine addition was still ongoing at P30 in *Neurod2* KO mice, in contrast to WT mice (Fig. 3k). Our synaptic analyses show that the development and turnover of dendritic spines in the apical tufts of L5 cPNs is altered in *Neurod2* KO mice.

Are dendritic spines altered in other cPN types? We injected an adeno-associated virus (AAV) inducing TurboRFP neuronal expression in L2/3 of M1 in *Neurod2* WT and KO mice, and measured spine density on infected neurons at P35 and >P120. No significant difference was observed at both ages in basal and apical compartments (Fig. S7f), suggesting that the spine phenotype in L5 neurons is not generalizable to all other cPN types.

### Increased excitability of L5 cPNs in *Neurod2* KO mice

The lamination phenotype as well as dendritic spine alterations observed in L5 cPNs at P30 prompted us to study the electrophysiological properties of these cells in Thy1-GFP; *Neurod2* KO mice with whole-cell patch-clamp recordings (Fig. S8a). The frequency and amplitude of somatic miniature excitatory post-synaptic currents (mEPSCs) were unaltered (Fig. S8b). The fact that the frequency of mEPSCs was not significantly reduced at P30 can be explained by the fact that spine density reduction was restricted to the apical tuft, a distal subcellular compartment where individual synapses are not likely to induce visible changes in somatic electrophysiological recordings [21]. When measuring miniature inhibitory PSCs (mIPSCs) we found no change in frequency but a small increase in amplitude in *Neurod2* KO mice (Fig. S8c). Since it was previously shown that L2/3 cPNs receive reduced mEPSC and mIPSC frequencies but normal amplitudes in *Neurod2* KO mice [14], our result implies that synaptic inputs are distinctly altered depending on the layer in *Neurod2* KO mice. We also calculated the E/I ratio based upon both frequency and amplitude values and saw no difference between *Neurod2* WT and KO neurons (Fig. S8d).

Next, we measured intrinsic physiological properties. Resting membrane potential as well as action potential threshold and amplitude were normal in L5 cPNs of *Neurod2* KO mice (Fig. S8e). Input membrane resistance and capacitance (Fig. S8f) showed a trend towards increased and decrease, respectively. More strikingly, L5 cPNs fired significantly more action potentials in response to depolarizing current injections in *Neurod2* KO mice (Fig. S8g), demonstrating increased intrinsic excitability. This phenotype was neither due to variations in action potential after-hyperpolarization (Fig. S8h)—which was the case for L2/3 cPNs in *Neurod2* in another study [14]—nor to alterations in resting membrane potential or action potential threshold and amplitude (Fig. S8e). Finally, we measured the hyperpolarization-activated cation (HCN) Ih currents that typically influence intrinsic neuronal excitability [22] and are critical integrators of synaptic integration in L5 cPNs [23]. Compared with WT mice, L5 cPNs in *Neurod2* KO mice exhibited an increased Ih current density (Fig. S8i), suggesting that *Neurod2* deficiency alters the expression or function of HCN channels. Since increased Ih current typically down-tunes intrinsic neuronal excitability [22], this phenotype might represent a compensatory mechanism that counter-balances *Neurod2*-induced neuronal hyper excitability.

### *Neurod2* loss and haploinsufficiency result in autism-like behaviors in mice

Because alterations in cPN migration, spine density, and turnover are strongly associated with neurobehavioral phenotypes [24–27], we investigated whether *Neurod2* loss and haploinsufficiency, the latter being more likely to match the situation of putative patients, could result in ASD-like phenotypes. We measured the two core features, that is, impairment of social interactions and stereotyped repetitive behaviors, which serve to diagnose ASD (DSM-5).

**Fig. 4:**
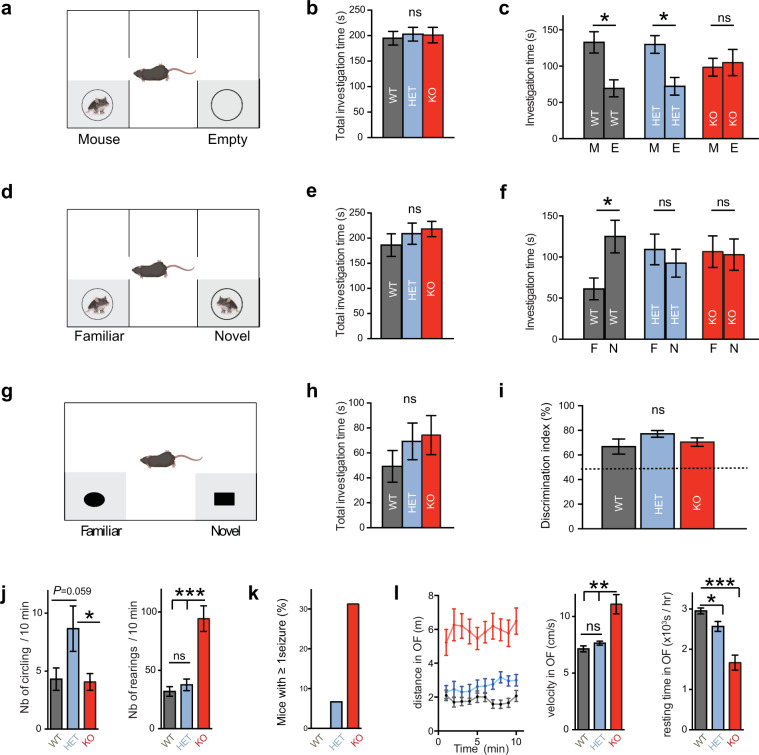
Autism-like behavior in *Neurod2* KO and HET mice. **a**–**c** During social interaction in the three-chamber test (**a**), all genotypes displayed similar total investigation time (**b**). However, while WT and HET mice spent around twice more time interacting with the mouse box than with the empty box, *Neurod2* KO mice spent equivalent times investigating mouse and empty box (**c**). **d**–**f** Social recognition/memory test. When a familiar and a novel mouse were placed in each box (**d**), all genotypes spent similar times having social interactions (**e**), but only WT mice spent significantly more time investigating the novel mouse, indicating altered social recognition or memory in HET and KO mice (**f**). **g**–**i** In the novel object recognition test (**g**), *Neurod2* KO and HET mice displayed unaltered total investigation time (**h**) and discriminated normally between the familiar and the novel object (**i**). Total investigation time is the time spent around left and right boxes (gray zones). **j** Circling (left graph) and rearing (right graph) behaviors in the 3 genotypes. **k** Spontaneous seizures were observed in a third of *Neurod2* KO mice and in one HET mouse during the behavioral experiments, but never in WT littermates. **l** Hyperactivity in *Neurod2* KO mice. Left graph depicts the distance traveled in 1 min-intervals during 10 minutes in the open field. Middle graph shows velocity during motion and right graph shows resting time, both during a 1-h recording. We used 19 WT, 22 HET, and 18 KO male mice, age-matched 8–14 weeks depending on the test. Data are means ± SEM. Statistical significance was evaluated by one-way ANOVA [(**b**), (**e**), (**h**), (**i**), (**j**), (**k**) and (**l**)] or two-way Mixed ANOVA followed by post hoc analysis using paired *t*-test [(**c**) and (**f**)] (ns, not significant; *P < 0.05; **P < 0.01; ***P < 0.001).

The first criterion, that is, impaired social interactions, was evaluated using the three-chamber test. In the sociability task, unlike wild-types the *Neurod2* KO mice did not investigate more frequently a conspecific than an empty box (Fig. 4a–c and Fig. S9a). *Neurod2* HET mice behaved similar to WT in this task, however (Fig. 4c and Fig. S9a). In the social recognition test, WT mice preferred interacting with the novel mouse, as expected (Fig. 4d–f and Fig. S9b). In contrast, both *Neurod2* KO and HET mice did not show any preference (Fig. 4f, Fig. S9b). Thus, sociability was altered only in *Neurod2* KO mice while social recognition/memory was impaired in both KO and HET mice. In contrast to the social tasks, interest and memory for objects were unaltered in *Neurod2* HET and KO mice, as shown in the novel object recognition task (Fig. 4g–i). This confirms the specificity of the social behavior alteration.

The second ASD criterion, namely repetitive patterns of behavior, was assessed by analyzing ASD-related stereotypic mouse behaviors such as grooming, circling, and rearing [28]. Because mice of all three genotypes displayed almost no grooming behaviors owing to the mixed C57BL/6 N;129 Sv genetic background, we focused on rearing and circling. As previously described [29], *Neurod2* HET mice displayed increased circling (Fig. 4j). Instead, *Neurod2* null mice showed increased rearing, both at and outside the walls of the cage (Fig. 4j).

Finally, we assessed mouse behaviors related to ASD comorbidities, such as epilepsy, change in locomotor activity (an ADHD-related phenotype), and conflict anxiety. During the course of the behavioral experiments, a third of *Neurod2* KO mice were observed having spontaneous epileptic seizures at the behavioral level (Fig. 4k and Video S1 for two examples). The seizures were lethal for half of KO mice for which seizures were observed (three out of six with seizures among 18 *Neurod2* KO mice). Two out of 22 HET mice were observed with a spontaneous epileptic crisis, while no WT mice showed behavioral seizure activity. The proportion of epileptic seizures among *Neurod2* KO and HET mice is likely an underestimate since mice were not monitored continuously.

**Fig. 5:**
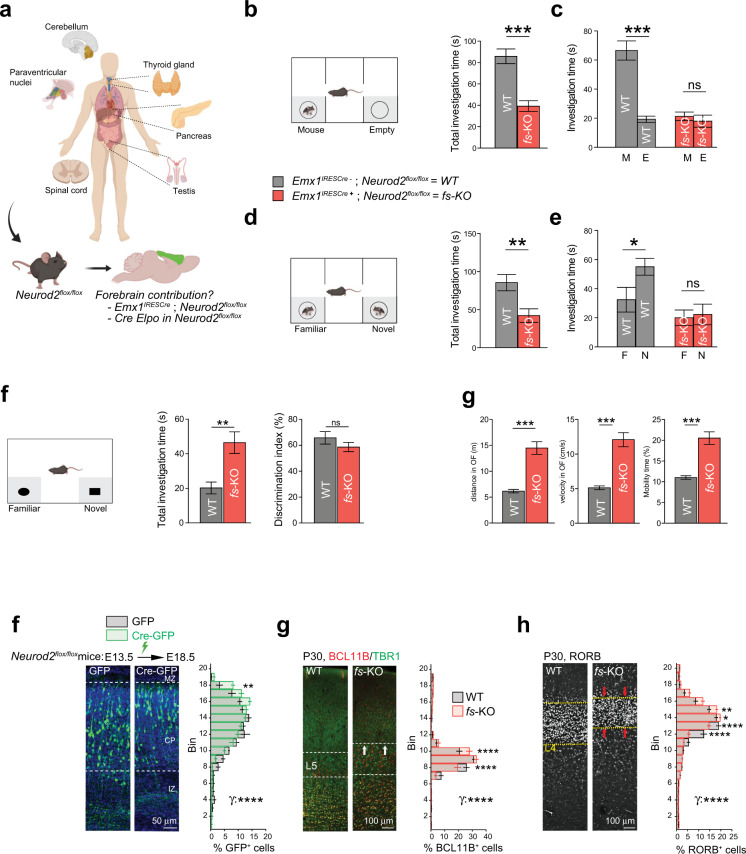
Forebrain excitatory neuron-specific *Neurod2* deletion recapitulates ASD-like phenotypes in mice. **a** Top scheme: Tissue types expressing *NEUROD2* in humans include paraventricular nuclei and thyroid hormone, which could account for ASD phenotypes in the *NEUROD2* syndrome. We generated *Neurod2*^flox/flox^ mice and crossed them with Emx1^IRES*Cre*^ mice to assess the specific contribution of forebrain excitatory neurons to the ASD-related phenotypes (bottom scheme). **b**–**e** Three-chamber assay. **b**, **c** Social interaction was altered in Emx1^IRES*Cre*^; *Neurod2*^flox/flox^ (cKO) mice. **d**, **e** Social recognition was reduced. Total investigation time is the time spent around left and right boxes (gray zones). This parameter was increased in *fs*-KO mice, which contrasts with the full KO mice. *N* = 11 WT and 9 *fs*KO mice. **f** Over-migration of cPNs was cell-autonomous, as shown by Cre electroporation in pallial progenitors at E13.5 followed by analysis at E18.5. 20-bin based distribution of electroporated cells is shown (for more analyses, see Fig. S10d). Mean cell laminar position in percentage of the cortical thickness, probability density function (bars representing maximal probability density), empirical cumulative distribution function, and 20-bin based distribution of electroporated cells are represented from left to right. *N* = 5 WT and 4 KO mice, five slices per animal counted. **g** Mean cell laminar position in percentage of the cortical thickness, probability density function (bars representing maximal probability density), empirical cumulative distribution function and 20-bin based distribution of cPN types using the markers RORB for L4. *N* = 7 WT and 6 KO mice, five slices per animal counted. Data are means ± SEM. Statistical analyses were performed using two-tailed *t*-tests or Mann–Whitney test depending on the normality of samples [(**b**), (**d**)], two-way mixed ANOVA followed by post hoc analysis using paired *t*-test [(**c**)], and by Permutation test for a spatially adjusted two-way ANOVA followed by Bonferroni’s post-hoc test [bin graphs in (**f**)–(**h**)] (ns, not significant; *P < 0.05; **P < 0.01; ***P < 0.001, ****P < 0.0001). See also Fig. S10.

*Neurod2* KO mice were hyperactive in the open field (Fig. 4l, Fig. S9c), while HET mice displayed only subtle hyper-locomotion compared to WTs (Fig. 5l). *Neurod2* KO mice displayed both higher displacement velocities and shorter resting periods (Fig. 5l).

In summary, *Neurod2* HET and KO mice exhibited behavioral defects reminiscent of the symptoms seen in ASD (altered social interest and memory, stereotypies) and ASD comorbidities (epilepsy, hyperactivity). *Neurod2* HET mice had clear phenotypes although milder than KO mice, indicating that *Neurod2* is a haploinsufficient gene.

### Forebrain excitatory neuron-specific *Neurod2* deletion recapitulates ASD-related phenotypes in mice

The cellular and behavioral phenotypes observed in *Neurod2* full KO mice could result from alterations in areas other than the forebrain where *Neurod2* is also expressed, such as paraventricular hypothalamic nuclei, cerebellum, spinal cord or even extra-CNS areas such as thyroid or pancreas (Fig. 5a). Furthermore, although we did not detect Neurod2 mRNA or protein in inhibitory neurons, it remains possible that *Neurod2* is expressed in these cells at developmental stages not analyzed here.

To evaluate the contribution of excitatory neurons of the forebrain to the ASD phenotypes, we generated conditional *Neurod2*^flox/flox^ mice in which the whole coding exon was flanked by loxP sites (Fig. S10a), and crossed them with Emx1^IRES*Cre*^ mice [30] to generate *forebrain* excitatory neuron *specific* KOs (from now on referred to as *fs-*KO mice). Strikingly, *fs-*KO mice recapitulated major ASD-related behavioral defects, as both social interaction and recognition were strongly altered compared to Emx1^IRES*Cre* -^ littermates (Fig. 5b–e). Like full *Neurod2* KO mice, *fs-*KO mice behaved normally in the novel object recognition task (Fig. 5f) and were hyperactive in the open field (Fig. 5g). We also observed occasional spontaneous seizures in *fs-*KO mice but never in Emx1^IRES*Cre *-^ littermates (second half of Video S1), which was consistent with the full KO situation. Thus, *fs-*mice recapitulate most behavioral phenotypes observed in *Neurod2* HET and KO mice.

We then tested whether the over-migration observed in full *Neurod2* KO mice was cell autonomous by electroporating Cre recombinase versus an empty plasmid in *Neurod2*^flox/flox^ embryos at E13.5. L5 cPNs from Cre-electroporated *Neurod2*^flox/flox^ mice indeed over-migrated as the average cellular population was shifted towards the pial surface (Fig. 5f). Because the absence of visible terminal translocation defect in constitutive HET and KO mice did not rule out the possibility that cell-autonomous *Neurod2* deletion could alter this migratory step [18], we measured the percentage of cells in MZ at five days post-electroporation. We did not observe significant amounts of ectopic cells in MZ (Fig. S10e), suggesting that *Neurod2* does not cell-autonomously regulate terminal translocation, at least for L5 cPNs.

The cell-autonomous nature of the over-migration phenotype suggested to us that *Neurod2* deletion only in forebrain cPNs might suffice to induce layer mispositioning as observed in *Neurod2* full KO mice at P30. Analyses of BCL11B and RORB immunostainings confirmed this hypothesis as *fs*-KO mice showed superficial switches of L4 and L5 cPNs (Fig. 5g, h and Fig. S10f), hence recapitulating the full KO phenotype.

### *Neurod2* deletion and heterozygosity alter the expression of neuronal voltage-gated ion channels

**Fig. 6:**
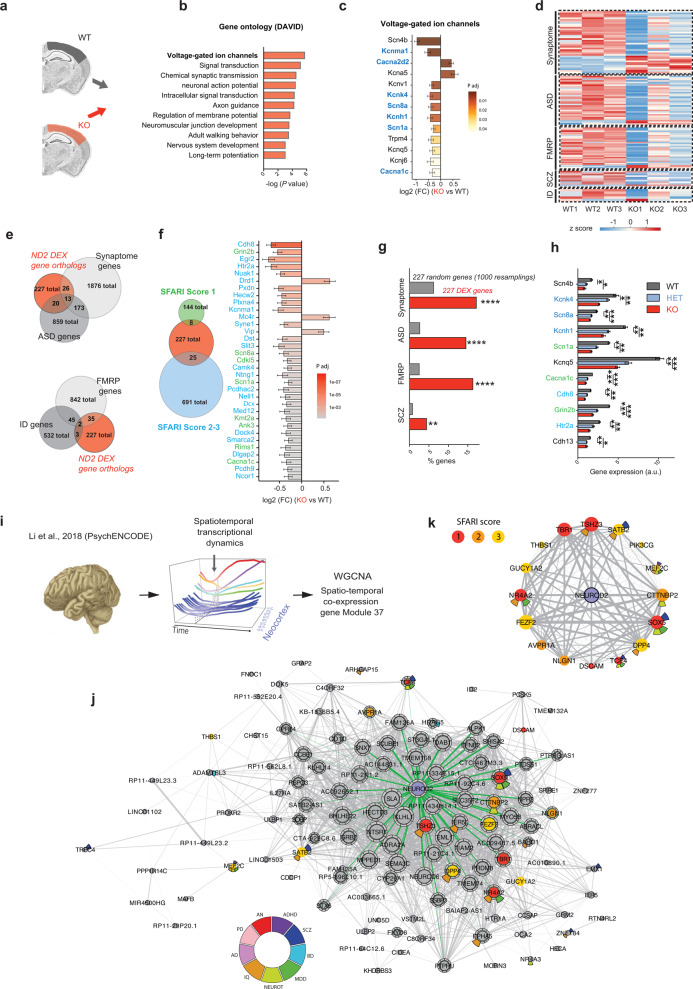
Molecular analyses in mice and humans place NEUROD2 as a nexus in NDD gene regulatory network. **a** Scheme of the RNA-seq experiment, performed at P30. **b** Gene set enrichment analysis using the DAVID knowledgebase. **c** Fold change expression (FC; log2 scale) of 13 DEX genes belonging to the voltage-dependent ion channel family, ranked according to *P*adj (lowest on top). Genes associated with neuropsychiatric recurrent syndromes are depicted in blue. **d** Heatmap for synaptic and disease gene sets among the orthologs of DEX genes (red, higher expression; blue, lower expression). **e**, **f** Venn diagrams identifying overlaps between DEX genes and synaptic/ NDD gene sets. Number of genes for each gene set is indicated. Right in (**f**), differentially-expressed genes associated with ASD and their score in SFARI. **g** Graphical representation of % [DEX genes] (orange) and % [same number of randomly selected genes] (gray) belonging to gene sets of interest. (**h**) qRT-PCR for 11 neuropsychiatric-related DEX genes in cortical samples from WT, HET, and KO. These data validate the RNA-seq results and show that HET mice have an intermediate phenotype between WT and KO mice, demonstrating that *Neurod2* is haploinsufficient. (**i**–**k**) *NEUROD2*-based coexpression network analysis in humans. **i** Gene module 37 with spatio-temporal co-expression was obtained from WGCNA of spatiotemporal transcriptional dynamic analyzes in the human cortex [49]. **j** Network representation of *NEUROD2* within module 37 from Li et al. [37] showing gene connectivity based on Pearson correlation (weight of edges scaled for *r* between 0.7 and 1). The size of each node is proportional to degree centrality calculated with a cutoff of *r* = 0.7. Green edges indicate direct neighbors of NEUROD2 with threshold *r* = 0.7. Genes described in SFARI are colored according from SFARI score (1–3). **k** Circular network representation of *NEUROD2* and SFARI genes interactions with weight of edges scaled for *r* between 0.5 and 1. The size of each node represents the same values of degree centrality as calculated in (**j**). For (**j**, **k**), we added a circle with nine cells. Each cell corresponds to a neuropsychiatric trait/disorder and color/position legend is at the bottom. For details on how neuropsychiatric trait/disorder cells were determined, see “Materials and methods” and File S1, sheet “GWAS_NEUROD2_GeneModule_Fig6”. *N* = 3 experiments per genotype for both RNA-seq and qRT-PCR. Data are means ± SEM. Statistical significance was evaluated by binomial test (**g**) or unpaired two-tailed Student *t* test (**h**) (*P < 0.05; **P < 0.01; ****P < 0.0001). See also Figs. S11 and S12.

To identify gene families and regulatory pathways most affected by *Neurod2* deletion in the neocortex, we performed Gene ontology (GO) analysis with DAVID (Fig. 6a, b and Fig. S11a) and ClueGO [31] on our bulk RNAseq dataset (Fig. S11b). DEX genes were most significantly enriched for voltage-gated ion channel (VGIC) activity, followed by cell projection morphogenesis and chemical synaptic transmission. We found 13 DEX VGICs including the sodium channels *Scn1a*, *Scn4b* and *Scn8a*, the potassium channels *Kcnh1*, *Kcnq5*, *Kcnj6*, *Kcna5*, *Kcnv1*, *Kcnk4* and *Kcnma1*, and the calcium channels *Cacna1c* and *Cacna2d2* (Fig. 6c), many of which are associated with neuropsychiatric syndromes (*Scn1a* in Dravet syndrome, *Cacna1c* in Timothy syndrome, *Kcnma1* in developmental delay and epilepsy, *Scn8a* in early epileptic encephalopathy and cognitive impairment, *Kcnh1* in Temple-Baraitser syndrome; blue in Fig. 6c). Among those 13 DEX VGICs, 11 were downregulated and 2 were upregulated (*Cacna2d2* and *Kcna5*). To test whether such voltage-gated ion channels were also dysregulated in *Neurod2* HET mice, we performed qRT-PCR for 6 of them. Strikingly, all of them were dysregulated like in full KO mice (Fig. 6h), hence demonstrating that these genes are sensitive to *Neurod2* haploinsufficiency.

### *Neurod2* deletion and heterozygosity modify ASD-associated and post-synaptic genes in mice

Because 227 of the 263 mouse DEX genes had a non-ambiguous human ortholog, we reasoned that examining their disease association could provide a valuable indication to *NEUROD2* function. An extensive PubMed search for the 227 human orthologs of DEX genes established that 190 of the 227 genes (83.7%) are putative causal loci for brain and/or nervous system disorders. ﻿Interestingly, the majority of these genes (146/190; 76.8%) have been associated with ASD, with the second most represented disease (5.8%) being schizophrenia (Fig. S12a, b and File S1 sheets “Orthologs_Disease(PubMed)_Fig6”).

In a complementary approach, we analyzed the overlap between the 227 orthologs of DEX genes and ASD-associated genes [32] from SFARI database (859 genes), FMRP-associated genes from Darnell and colleagues [33] (842 genes), intellectual disability-associated genes from multiple sources [34], schizophrenia-associated genes ([35] and OMIM, 196 genes) and synaptome-associated genes [36] (SynaptomeDB, 1876 genes). We generated a heatmap of expression levels for overlapping genes (Fig. 6d). Venn diagrams showed that among the 227 human orthologs of DEX genes, 39 (17.2%) were associated with the synaptome, 33 (14.5%) with ASD, 37 (16.3%) with FMRP, and 10 (4.4%) with schizophrenia (Fig. 6e, f and File S1 sheet “Orthologs_NND_asso_Fig6”). These proportions are much higher than what a random sampling would produce (Fig. 6g and File S1 sheet “sheet “Orthologs_NND_asso_Fig6” for binomial test with 1000 resamplings; hypergeometric test gave comparable results). Among DEX ASD genes, 8 were SFARI score 1 and 25 SFARI score 2–3 (Fig. 6f). These ASD genes included the Dravet syndrome gene *SCN1A* and the Timothy syndrome gene *CACNA1C*, but also *GRIN2B*, *HTR2A*, *PCDH9,* and other genes (Fig. 6f). Among the synaptome-related DEX genes, 4 were pre-synaptic and 36 were post-synaptic [36] (Fig. S12c), suggesting that *Neurod2* primarily regulates the post-synaptic element of synapses. Finally, qRT-PCR for 11 DEX genes representative of different expression profiles and disease associations (*Kcnh1* and *Cdh8* with expected L2/3 enrichment, *Kcnq5* and *Htr2a* with L5 enrichment, *Scn4b*, *Kcnk4*, *Scn8a*, *Scn1a*, *Cacna1c,* and *Grin2b* with L2-6 enrichment) showed an expression level in HET mice that was either intermediate between WT and KO or even undistinguishable to the KO level. This indicates that cortical *Neurod2* target genes are sensitive to *Neurod2* haploinsufficiency.

### *NEUROD2* is a nexus in a human brain developmental gene network whose defects are associated with ASD

Can integrated functional genomics in humans predict *NEUROD2* pathogenicity? We studied the localization of human *NEUROD2* within the 73 gene co-expression modules observed by weighted gene correlation network analysis (WGCNA) from human neocortical samples spanning the entire lifespan from psychENCODE [37]. Strikingly, NEUROD2 is central in gene co-expression module 37, which is composed of 145 genes enriched in neurons with fetal enhancers. This module shows exceptional enrichment in association signals for multiple neuropsychiatric traits and disorders, including intellectual disability, schizophrenia, neuroticism [37] (Fig. 6j) as well as in genes related to ASD from the analysis of de novo mutations [38], associated to developmental delay [39] and/or described in SFARI (Fig. 6j, k). Even more interestingly, NEUROD2 was one of the most strongly connected gene in module 37 (see the large size of circle in Fig. 6j, k), and presented highly correlated expression with genes listed in SFARI with scores from 1 to 3, including *TCF4, TSHZ3*, *TBR1*, *NR4A2* and *SOX5* (Fig. 6j, k).

Altogether, our molecular analyses in mice and humans point to *NEUROD2* as a central node in a brain developmental gene regulatory network whose defects are associated with NDDs including ASD.

### *NEUROD2* pathogenic mutations cause ASD and intellectual disability in humans

Our neurobehavioral data in mice and genetic data in mice and humans suggest that *NEUROD2* loss-of-function or haploinsufficiency might be associated with NDDs. Three *in silico* evidences are consistent with this hypothesis. First, assessment of *NEUROD2* mRNA in prediction algorithms for microRNA-mRNA interactions (TargetScan, http://www.targetscan.org/; miRanda, http://mirdb.org/) show the existence in its 3′UTR of a high-confidence 8-mer target site for the schizophrenia-associated microRNA miR-137 [40], suggesting that NEUROD2 might be involved in the pathogenesis of this disease. Second, copy number variations on chromosome 17 that encompass *NEUROD2* are systematically associated with NDDs for 13/13 patients (https://decipher.sanger.ac.uk/); the most represented phenotypes here are intellectual disability and ASD. And third, a machine learning prediction algorithm for autism-associated genes, based on already known ASD genes in the context of a human brain-specific gene interaction network, ranks *NEUROD2* 98^th^ among 25,825 human genes (https://asd.princeton.edu/genesets/) [41] a better rank than many confirmed ASD genes. These *in silico* data reinforce our results in positioning *NEUROD2* as a node in a gene regulatory networks associated with NDDs and in particular ASD. Guided by this knowledge, we sought to identify individuals with pathogenic mutations in *NEUROD2*.

**Fig. 7:**
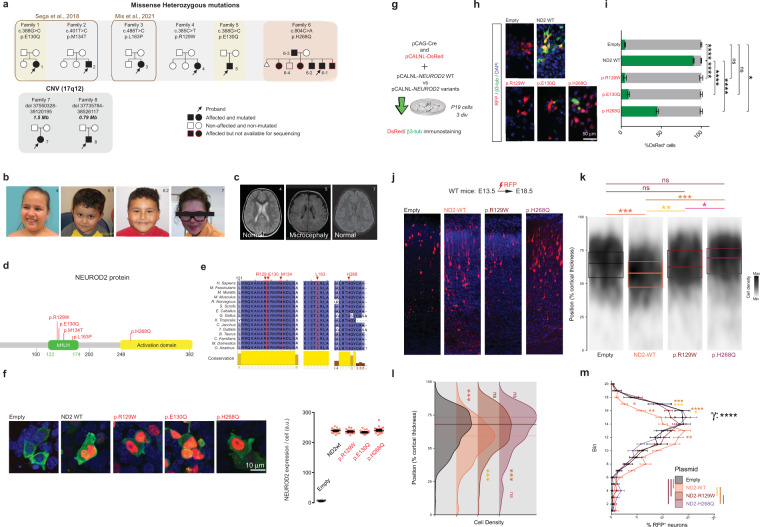
*NEUROD2* pathogenic mutations cause an ASD and intellectual disability syndrome. **a**–**c** The clinical phenotype of *NEUROD2* syndrome (see also Table 1). **a** Pedigrees of 8 families with *NEUROD2* disruptions (Families 1 to 6 with pathogenic missense heterozygous mutations, Families 6 and 7 with deletions). Yellow: Families 1 and 5 share the same mutation, pE130Q. Red: Family with inherited mutation. Patients 6-1 and 6-2 in family 6 share the same mutation with their father (patient 6–3). Sanger sequencing demonstrated that their healthy brother 6–4 does not carry the mutation in *NEUROD2*. **b** Affected individuals showed slight to no dysmorphic facial features (shown are pictures from individuals in families 4, 6, and 7). **c** Axial T2-weighted MRI images of patients 4, 5, and 7 showing no obvious abnormality in brain organization. Note that Patient 5 displayed microcephaly. **d** Localization of NEUROD2 mutations analyzed in this study, that is associated with intellectual disability/ASD. **e** ﻿Phylogenetic conservation of residues substituted in NEUROD2 for the six patients with missense mutations. The positions of substituted residues are indicated in red. In silico multiple sequence alignment was performed using Clustal Omega and Jalview. **f** Left, photomicrographs of HEK cells transfected with *NEUROD2* variants immunolabeled for NEUROD2 protein. Right, NEUROD2 fluorescence intensity was measured for wild type NEUROD2 and each variant. **g–i** Demonstration of the pathogenicity of *NEUROD2* variants using a novel in vitro test. **g** P19 cells were transfected with pCAG-Cre, pCALNL-RFP, and a plasmid containing different variants of Cre-dependent human *NEUROD2* (pCALNL-*NEUROD2*). **h** Representative images of RFP-transfected neurons immunostained for β3-tubulin (green) after 3 days in vitro. **i** The percentage of RFP^+^ cells expressing β3-tubulin in each condition was counted (*n* = 3 independent experiments, three coverslips per experiment). **j**–**m** p.R129W and p.H268Q variants are loss-of-function for migration in vivo. Pallial progenitors in WT mice were co-electroporated with RFP and an empty vector, NEUROD2_WT, p.R129W or p.H268Q at E13.5. Cell distribution analyses were performed at E18.5. **j** Representative images for each of the four conditions illustrating the distribution of RFP-expressing cells. **k** Cell laminar position in the percentage of the cortical thickness for each condition. **l** Probability density function showing the average laminar distribution of cells. **m** Twenty-bin-based laminar distribution of RFP-expressing cells in each condition. We analyzed 11 slices from 3 Empty vector, 21 slices from 5 NEUROD2_WT, 23 slices from 5 p.R129W, and 15 slices from 4 p.H268Q electroporated mice. Data are represented as means ± SEM. Statistical significance was evaluated by one-way ANOVA followed by Tukey’s multiple comparisons post hoc test. **i** Pairwise permutation test across groups for medians [(**k**)], Anderson-Darling test [probability densities in (**l**)] or by Permutation test for a spatially adjusted two-way ANOVA followed by Bonferroni’s post-hoc test [bin graphs in (**c**–**f**)] (*P < 0.05, **P < 0.01, ***P < 0.001, ****P < 0.0001). Consent was obtained to publish the clinical images. See also Table 2, Fig. S13 and File S1.

Through a collaborative effort, we gained access to 6 families with *NEUROD2* heterozygous missense mutations associated with NDD including intellectual disability, ASD and speech delay (Table 1 and Fig. 7a). Families 1 and 2 are followed up from published patients (p.Glu130Gln and p.Met134Thr) who suffered early epileptic encephalopathic seizures from 5 to 16 months of age [10]. Family 3 is a follow-up from a very recent study of a single patient with NDD (p.Leu163Pro) [11] thus insufficient to firmly conclude the association between mutation and disease. Families 4 to 6 are new to this study. Family 4 carries a newly identified *de novo NEUROD2* mutation, p.Arg129Trp. Family 5 is also newly identified; interestingly, it carries a patient with the exact same mutation as Family 1, p.Glu130Gln (both in yellow in Fig. 7a). The fact that patients from Families 1 and 5 carry the same mutation and display very similar symptoms (see below and Table 1) strongly suggests that the mutation is causative. Family 6 is also newly identified. It is a non-consanguineous family with a newly identified *NEUROD2* mutation (p.His268Gln) transmitted from the affected father to his five affected children, but not to his only unaffected child (6/8 individuals affected; 1 healthy child; Fig. 7a–c).

**Table 1:** Clinical features of individuals with NEUROD2 mutations.

Patient	1 (from Sega et al. [10])	2 (from Sega et al. [10])	3 (from Mis et al. [11])	4	5	6-1	7	8
Mutation	c.388 G> C: p.E130Q	c.401 T> C: p.M134T	c.488 T> C: p.L163P	c.385 C> T: p.R129W	c.388 G> C: p.E130Q	c.804 C> A: p.H268Q	1.57 Mb *deletion* (55 genes)	0.79 Mb *deletion* (37 genes)
Sex	Female	Male	Female	Female	Male	Male	Female	Male
Age	5 years	3.8 years	16 years	11 years	21 months	5 years	19 years	9 years
Inheritance	De novo	De novo	De novo	De novo	De novo	From affected father	De novo	De novo
Birth weight (g)	3175	Unknown	2778	4000	3175	3402	1300	2400
Birth length (cm)	Unknown	Unknown	49	unknown	51.4	50.8	38	unknown
Birth head circumference (cm)	Unknown	Unknown	Unknown	unknown	31.8	unknown	29	unknown
Age at last examination (years)	4.5	3.4	13	9	21 months	3	18	9
Weight at last examination	−2.47 SD	37th percentile	36.7 kg	>+2.5 SD	9.48 kg	46.9 kg	51 kg	Not collected
Height at last examination	−2.63 SD	41th percentile	147.5 cm	−1 SD	81.5 cm	165.2 cm	149 cm	Not collected
Head circumference at last examination	−2.49 SD	93th percentile	52 cm	53	43.5 cm <1 %ile (Z = −2.88)	54.3	52	52
Failure to thrive	Yes	Yes	Yes	No	No	No	Yes	No
Head control delay	Yes (3.5 years)	Yes (18 months)	ND	No	Yes	No	Yes	No
Walking delay	Yes (still needs a walker)	Yes (2 year 6 months)	No (18 months)	No	Probably Yes (not yet)	No	Yes (2 years)	Yes (3 years 6 months)
Epilepsy	Yes infantile spasms, onset age 5 months, seizure free since the age of 2.5 years. Started on the ketogenic diet at 16 months, discontinued at 3 years	Yes infantile spasms, onset age 5 months	No	No	Yes infantile spasms at 4 months	No	Unsure because of a stereotypic behavior remaining day and night, a 24 h EEG monitoring is planned (no results yet)	No
Intellectual Disability	Yes *Severe cognitive dysfunction, is not able to understand simple commands	ND	Yes *IQ in the borderline range *Per last exam, in school with IEP and a paraeducator.	Yes *IQ test 32	Yes	Yes	Yes	Yes
Speech disturbance	Yes *5 single words only, no sentences	Yes *at 3-years-5-months has vocalizations but not much consonants or intentional vowels	Yes *Started talking at about 2.5 years	Yes *First words at the age of 3 years	Yes *Delayed	Yes *single words only	Yes *Severely delayed, can speak words	Yes *Limited verbal comprehension to a few simple commands; no verbal expression.
Autistic features/Behavioral disorder	ND	Autism *Lacks reciprocal play and imitation	ASD *Responsiveness to environment falls outside expectations * Nonverbal learning disorder *Limitations in motor skills and visual/spatial ability	Autism	ASD	ASD	ASD *Mainly stereotypic behavior	ASD *Agitated child. He hits his head, he is aggressive to himself and towards others. He screams. Treatment with risperidone for behavioral disorder.
ADHD/aggressiveness	Yes *Hyperkinetic movements	Yes *Hyperactive behavior	No	No	ND	Yes *Aggressive and hyperactive behaviors	No	Yes *Aggressive and hyperactive behavior
MRI results	bilateral increased T2 signal intensities in putamina, parietal periventricular white matter, diffuse thinning of corpus callosum	At 10 months: mild, generalized cerebral volume loss, absence of the posterior pituitary T1 bright spot	At 3 years: Normal	Normal	MRI in 9/2019 showed Large bilateral tympanomastoid effusions	ND	Mildly enlarged Virchow-Robin space	Normal
Other clinical features	Bilateral esotropia, hyperkinetic movements	Dysphagia (thickened feed)	Self-resolved ventricular septal defect. Irregularly bordered café-au-lait spot on right lower back	Central obesitas, large (long) frontal teeth, tapering fingers	Microcephaly, hypotonia, global developmental delay, feeding difficulties, constipation	Inverted nipples, clumsy	Atrium septal defect, unilateral kidney dysplasia, syrinx in spine with secondary spasticity of the left leg, unilateral corral leukoma, hypothyroidism	Mild unspecific facial dysmorphism (low implanted ears), asthma

*ASD* autism spectrum disorder, *MRI* magnetic resonance imaging, *ND* not determined, *SD* standard deviation, %ile, percentile.

The three novel *NEUROD2* variants from Families 4 to 6 were identified by whole-exome sequencing and confirmed by family-based Sanger sequencing. De novo mutations Glu130Gln, Met134Thr, and Arg129Trp from Families 1, 2, and 4 are found in the conserved DNA-binding region of the bHLH domain, which is the basic region of the first helix (Fig. 7e, f). The de novo Leu163Pro mutation from Family 3 lies in the second helix of the bHLH domain thought to be important for DNA binding and is also highly conserved (Fig. 7d, e) [11]. The Histidine 268 of inherited His268Gln mutation from Family 6, and its close surroundings, are also highly conserved despite the fact that they lie in the more poorly conserved activation domain (Fig. 7d, e). The *NEUROD2* gene is very intolerant to variations, with a constraint *Z* = 4.59 for missense variants (ExAC) and a Residual Variation Intolerance Score (RVIS) of 28.9% (Genic Intolerance). All five *NEUROD2* variants from Families 1–6 are extremely rare or unique, as none can be found in ﻿any human mutation databases including the gnomAD cohort of 141,456 individuals, and are predicted to be deleterious by Combined Annotation-Dependent Depletion (CADD) [42] (File S1, sheet “NEUROD2_PathoPredict_Fig.7”). Importantly, no other variants besides *NEUROD2* were identified in known disease-causing genes that could account for the clinical phenotypes in any of the 6 families.

In additional, we also gained access to Families 7 and 8, two families with the smallest available de novo 17q12.3-q21 interstitial deletions (1.57 and 0.79 Mb) that include *NEUROD2*. The two deletions comprise 55 and 38 genes, respectively (Fig. 7a–c, Fig. S13). The patients with de novo deletions in Families 7 and 8 share the same core phenotypes with the 6 families with heterozygous missense *NEUROD2* mutations. Moreover, OMIM and Pubmed searches indicate that, besides *NEUROD2*, none of the other deleted genes in these deletions is predicted to contribute to these phenotypes (File S1 sheet “Genelist_DeletionPatients_Fig7” for more information about these genes). This suggests that NEUROD2 deletion might be, at least in part, responsible for the phenotypes in Families 7 and 8.

Clinically, all affected patients from Families 1 to 8 had normal or only slightly dysmorphic facial features (Fig. 7b) and grossly normal brain MRIs (Fig. 7c), but presented clinically with a core spectrum of phenotypes including intellectual disability, ASD, and speech disturbance (Table 1). Comorbidities include epilepsy (3, potentially 4/8), hyperactivity/ADHD (4/7), motor delay (5/8), and failure to thrive (4/8) (Table 1). Detailed MRI analysis identified diffuse corpus callosum thinning and slight microcephaly in patients from Families 1 and 5, respectively, who both carry the same Glu130Gln mutation. Such anatomical features are consistent with the observations made in our *Neurod2* mutant mice (Fig. S2).

The facts that (1) 7 patients (6 families with missense mutations, including Patients 6-1 and 6-2 from Family 6) share extremely rare *NEUROD2* variants with pathogenic prediction and similar NDD/ASD symptoms, (2) Patients 1 and 5 carry the same mutation (p.Glu130Gln) and show the same symptoms, and that (3) inheritances of His268Gln mutation and NDD symptoms are co-segregated in Family 6, strongly reinforce the notion of a causative link between *NEUROD2* mutations and NDD. Nevertheless, the pathogenicity of variants ought to be measured directly. First, quantitative analyses of NEUROD2-immunolabeling in HEK cells 3 days after their transfection with each variant demonstrated normal protein levels, indicating that none of the variants altered NEUROD2 protein production (Fig. 7f). The pathogenicity of Glu130Gln, Met134Thr, and Leu163Pro mutations from Families 1 to 3 has been examined by variant overexpression in tadpoles [10, 11]. In order to test the pathogenicity of Arg129Trp and His268Gln in mammalian cells and to verify the transposability of tadpole tests to mammalian cells for Glu130Gln (shared by Patients 1 and 5), we took advantage of the fact that ﻿overexpressing human *NEUROD2* in P19 mouse embryonic carcinoma cells induces potent neuronal differentiation, a phenotype that relies on binding to DNA through E boxes [43–45] (Fig. 7g). Quantitatively, 90.5% of P19 cells transfected with human wild-type *NEUROD2* differentiated into β3-tubulin expressing neurons, while only 4.7% did so after transfection of a control plasmid (Fig. 7h, i). In contrast to wild-type *NEUROD2* however, transfection of either Arg129Trp or Glu130Gln variants did not induce any neuronal differentiation as the percentage of neurons was similar to a control transfection (4.4 and 9% for Arg129Trp and Glu130Gln, respectively). His268Gln transfection gave an intermediate phenotype, with significantly less potent neuronal induction as compared to wild-type *NEUROD2* (45.1% versus 90.5%) (Fig. 7h, i). Notably, for each variant the severity of the phenotype was correlated with the CADD score [42]. Given that neurogenesis induced by *NEUROD2* in P19 cells exclusively relies on direct DNA binding [45], these results indicate that the new *NEUROD2* variants in our study are pathogenic, via the induction of a loss of *NEUROD2* transcription factor activity.

Finally, we tested the pathogenicity of two representative *NEUROD2* mutations directly in vivo. We chose Arg129Trp as this mutation is both new and represents the bHLH hotspot, and His268Gln as it is the only one found in the activation domain. At E13.5, C57BL/6J mice were electroporated with Arg129Trp or His268Gln encoding plasmids, and cell positions were analyzed at E18.5. We reasoned that electroporation of a *NEUROD2* loss-of-function variant should leave the radial migration of cPNs not or less altered than electroporation of wild type *NEUROD2*, which reduces migration (Fig. 2c). As expected, in utero electroporation of C57BL/6J mice with either Arg129Trp or His268Gln encoding plasmids did not alter neuronal positioning, while electroporation with wild type *NEUROD2* did (Fig. 7j–m). Suprisingly, His268Gln electroporated mice showed laminations identical to controls, which contrasts with the in vitro assay where His268Gln induced an intermediate phenotype situated between control and wild type *NEUROD2* transfection. This suggests that the in vivo regulation of cPN migration by NEUROD2 strongly relies on its activation domain, possibly through activity-dependent modifications [9].

## Discussion

In the present study, we identified *NEUROD2* as a novel gene associated with NDDs. The characteristic clinical features of *NEUROD2*-mutated patients include autistic traits, intellectual disability, and speech disturbance. Missense mutations occurred de novo for 5/6 patients while it was inherited from an affected parent in a familial case. Furthermore, two patients from different families who carried the exact same amino-acid change also shared very similar symptoms, overall supporting the causative link between *NEUROD2* mutation and the syndrome. While a previous study has suggested that NEUROD2 is involved in early epileptic encephalopathy [10], our data point to a core NEUROD2-associated phenotype centered on ASD, intellectual disability, and speech disturbance. Non-fully penetrant *NEUROD2*-associated phenotypes include ADHD symptoms (5/7 patients) and epilepsy (3/7 patients). The pathogenicity of representative variants was demonstrated both in vitro in mouse cells and in vivo during mouse cortical development. Of interest for clinicians, testing the pathogenicity of novel *NEUROD2* variants shall now be quick and easy by using the P19 in vitro assay.

The causative link between *NEUROD2* disruption and the reported clinical symptoms is strengthened by the mouse studies. Indeed, *Neurod2* mutation in the mouse recapitulates most features of the human phenotype: ASD-relevant social abnormalities and propensity to epilepsy and hyperactivity. Also, the spatio-temporal expression of NEUROD2 reinforces the concept, emerging from single-cell sequencing data of the human brain [46], that glutamatergic projection neurons at mid-gestation and during adulthood are the brain cells that are the most highly enriched in ASD genes. Interestingly, the DECIPHER human genome database reports that a small, 135 kb-long duplication encompassing the whole *NEUROD2* gene, is associated with intellectual disability and delayed language development. This suggests that not only reduced but also increased expression levels of functional NEUROD2 generate comparable symptoms, similar to what has been reported for the high confidence ASD genes *MECP2* [47] and *SHANK3* [48].

Our unbiased coexpression network analysis in humans using psychENCODE [37] positioned *NEUROD2* in gene coexpression module 37 that shows an exceptional enrichment in association signals from multiple neuropsychiatric traits and disorders, including ID and ASD [38]. *NEUROD2* presented a high degree of centrality within this module (Fig. 6i–k), and showed strongly correlated expression with high confidence ASD genes listed in SFARI, including *TCF4, TSHZ3*, *TBR1*, *NR4A2,* and *SOX5* (Fig. 6k). These data provide support for a hub position of *NEUROD2* in a cortical transcriptional regulatory network associated with NDDs including ID and ASD.

Our study in the mouse, showing gene expression variation in the cerebral cortex, enrichment of ASD-related genes in orthologs of DEX genes, anatomic/functional alterations in neural circuits formed by cPNs and behavioral abnormalities associated with *Neurod2* conditional deficiency, provide some clues to the causative link between NEUROD2 deletions and ASD.

Dysregulation of cortical migration and/or positioning of cPNs in the CP are commonly associated with NDDs [27], but the genetic and transcriptional mechanisms underlying these processes in health and disease remain poorly documented. Our results indicate that *Neurod2* cell-autonomously regulates cortical radial migration and laminar positioning of cPN subtypes. cPNs over-migrated in *Neurod2* KO, HET, and Emx1^IRES*Cre*^; *Neurod2*^flox/flox^ mice, and Cre electroporation in *Neurod2*^flox/flox^ mice also induced over-migration, indicating a cell-autonomous role. When investigating the cellular mechanisms, we did not find terminal translocation to be altered in KO, HET, and Cre-electroporated *Neurod2*^flox/flox^ mice. This result contrasts with a previous study where acute *Neurod2* knock-down was performed by in utero electroporation of a *Neurod2* shRNA [18]. The apparent discrepancy between this study and our results of Cre electroporation in *Neurod2*^flox/flox^ mice, which both aim to measure the cell-autonomous impact of *Neurod2*, might be explained the different experimental timelines used (Guzelsoy et al. used E14.5-E17.5 while we used E13.5-E18.5) and/or by possible nonspecific effects of the shRNA against *Neurod2* used in their study, as no rescue experiments were shown. In contrast to terminal translocation, our results point to altered cell shapes of new neurons in the IZ with cells being more elongated and bulkier in KO and HET mice. These quantitative changes suggest an acceleration of the transition from the round, small, and slowly migrating multipolar cells to larger, more elongated, and rapidly migrating bipolar neurons. Noteworthy, genetically induced hypo-migration phenotypes have been associated with increased branching in IZ neurons [49]. The fact that we did not make the opposite observation, namely reduced branching in IZ neurons, in our hyper-migration mouse, suggests that branching is minimal in control conditions and can only be augmented.

The molecular mechanisms downstream of *Neurod2* that are responsible for the over-migration remain to be discovered. Our gene ontology analyses of a Chip-seq dataset from the E14.5 cortex [17] revealed 136 migration-related genes, 28 of which are important regulators of radial migration (Fig. S4a). Our literature survey showed that multipolar-bipolar transition was directly associated with 16 of these 28 genes while glia-guided locomotion was associated with only six of these genes (File S1, sheet “egoE14.5_Chip_Seq_neuronMigrati_Disc”), consistent with multipolar-bipolar transition being the primarily affected process. In the future, it will be interesting to shorten the list of candidates by crossing this Chip-seq list with a RNAseq list from age-matched samples, and to perform rescue experiments with the best candidates in order to identify the main genetic factors impacting radial migration and subsequent neuronal positioning in *Neurod2* mutant mice.

Similar to our study, cPN over-migration phenotypes have been reported, for example after *in utero* overexpression of human *APP* [50], *Rac1* and its interactor *POSH* [51] or *Camk2b* [52]. After *APP* overexpression however, over-migration was transient since it was not visible anymore at 6 days post-electroporation. The long-term impact of ectopic expression of *Rac1*, *POSH,* or *Camk2b* on cortical lamination has not been described [50–52]. In our study, cPN over-migration was followed by an altered final laminar positioning and thickness of layers, within particular L5 expending superficially at the expense of L4, which consequently was thinned. This is to our knowledge the first time that such a phenotype is described. Since even subtle alterations in cPN positioning are sufficient to alter cortical output [53], this abnormal laminar cPN positioning likely participates in the disruption of cortical function and animal behavior.

We observed only a slight corpus callosum size reduction and no obvious axonal targeting specificity defects in *Neurod2* null mice, which contrasts with the total absence of corpus callosum in *Neurod2*/Neurod6 double mutant mice [54]. A likely explanation resides in the functional redundancy between *NeuroD* family members regarding axonal growth and navigation. In contrast, we suggest that the observed migratory and synaptic phenotypes are specific to *Neurod2* deletion among other *NeuroD* family members.

Our study indicates that some electrophysiological phenotypes are specific to cPN subtypes while others are generic. Although *Neurod2* is expressed in all cPN subtypes, L2/3 cPNs in *Neurod2* KO mice display reduced frequencies of both mEPSCs and mIPSCs [14], while L5 cPNs show slightly increased mIPSC amplitude (this study). Hence, *Neurod2* deletion appears to alter synaptic properties in a different manner depending on the cPN type and layer. In contrast to this layer-specific alteration of synaptic inputs, intrinsic excitability was increased similarly in both L2/3 and L5 cPNs, suggesting that the control of excitability might be a generic effect of *Neurod2* deficiency. This hypothesis is consistent with unpublished preliminary results indicating increased excitability of hippocampal CA1 PNs in *Neurod2* KO mice in vivo (Jérôme Epsztein, personal communication). However, the mechanisms by which *Neurod2* limits intrinsic excitability are likely different between L2/3 and L5 cPNs since decreased after-hyperpolarization was found as an underlying cause in L2/3 [14] but was unmodified in L5 cPNs (this study). The cause of intrinsic excitability in L5 cPNs of *Neurod2* KO mice requires further investigations and might involve axon initial segment (AIS) plasticity as many AIS VGICs were found to be dysregulated in our RNAseq.

A literature-based search of our 263 P30 DEX gene list and their human orthologs establishes a large number of *Neurod2* targets (direct or indirect) that might be causally associated with the cellular and behavioral phenotypes of *Neurod2* KO mice. The decreased expression of many L2/3 genes and increased expression of many L6 genes in Fig. 1m might at first sight suggest cPN subtype specification switches in *Neurod2* KO mice (Fig. 1m). However, several observations strongly argue against this possibility. First, based on public databases, the dysregulated genes in question are enriched in, but not specific to, L2/3 or L6, and none of them is a known critical player of cPN subtype specification. Furthermore, for some of these dysregulated genes the layer enrichment classification was based solely on in situ hybridization images from public databases, such that it is unclear whether they are expressed and dysregulated in cPNs, other cell types, or both. Second and more importantly, we found (i) normal numbers of neurons expressing the L2/3 and L6 cPN master regulator proteins CUX1 and TBR1, respectively (Fig. 1h), (ii) unaltered expression of known master regulators (such as *Cux1*, *Satb2,* or *Tbr1*) in our RNA-seq and (iii) normal output connectivity of L2/3 and L6 cPNs through CTB retrograde tracings after injection in typical target regions (Fig. S2). Thus, cells with the major molecular and morphofunctional properties of L2/3 and L6 projection neurons are produced in normal amounts in *Neurod2* KO mice. Therefore, the analyses in Fig. 1m do not reflect a bona fide subtype specification switch (from L2/3 to L6) but rather indicate differential dysregulation of gene expression between L2/3 and L6 cPNs. Further high-resolution experiments such as single-cell RNA-seq will be instrumental in determining the precise alterations in gene expression in cPN subtypes of different layers and areas.

Besides the over-migration phenotype, we also investigated dendritic spine density and turnover. First, our *Neurod2* KO DEX gene list contained as many as 39 synaptome genes, comprising 5 presynaptic and 34 post-synaptic genes. Second, dendritic spine density and turnover were dysregulated in apical tufts, but not basal dendrites of L5 cPNs. While over-migration was a general phenotype as it was observed in cPNs destined to different layers, the spine density and turnover phenotype was found in L5 neurons but not L2/3 neurons. L2/3 neurons showed overall normal spine densities in basal and apical dendrites, which might be considered surprising given that reduced miniatures post-synaptic currents were described for these cells in a previous study [14]. In the future, it will be interesting to determine whether and how the functionality of excitatory synapses might be altered in L2/3 neurons of *Neurod2* KO mice.

Among our 39 DEX synaptome genes at P30, two particularly good candidates for the alteration of apical tuft dendritic spine density and turnover are the glucocorticoid receptor gene *Nr3c1* and the synaptome gene *Syne1* (CPG2 isoform), which are both downregulated in *Neurod2* KO mice. *Nr3c1* is a critical regulator of dendritic spine development and plasticity in cPN apical dendrites [55]. Acute and chronic stresses both alter spine plasticity preferentially in the apical tuft of cPNs through glucocorticoid binding to *Nr3c1* [56]. This in turn provokes internalization and transcriptional activity of *Nr3c1*. Interestingly, a recent in vitro study indicates that the transcriptional activity of *Nr3c1* requires *Neurod2* as a cofactor [57]. Of note, *Neurod2* also interacts with the mineralocorticoid receptor *Nr3c2*, which is also involved in stress-dependent spine plasticity [55] and a newly identified ASD factor [46]. The hypothesis that *Neurod2* is involved in the stress pathway is corroborated by the fact that *Neurod2* KO mice show fearless behaviors in the elevated plus maze and during fear conditioning [6] and by the ADHD/aggressiveness symptoms in 4/7 of the *NEUROD2*-mutated patients. These observations suggest that *Neurod2* might be a nexus in stress-related synaptic plasticity in cPN apical dendrites. Besides *Nr3c1*, the *Syne1* isoform *CPG2* encodes an actin-binding protein regulating activity-dependent glutamate receptor endocytosis and RNA transport to nascent postsynaptic sites [58]. Interestingly, pathogenic *CPG2* variants are associated with bipolar disorders [59]. Although *Nr3c1* and *Syne1* are obvious candidates, other genes among the 33 post-synaptic and 4 pre-synaptic DEX genes might participate in *Neurod2* spine phenotypes.

Our DEX gene list also provides putative candidate genes for other phenotypes. Increased cPN excitability, locomotor hyperactivity and propensity to spontaneous epileptic seizures observed in *Neurod2* KO mice all suggest an overall increased cPN activity, and obvious candidate DEX genes for these phenotypes also lie among the 13 DEX VGICs. In particular, *Kcnq5* and *Scn8a* are both expressed only in cPNs and implicated in epilepsy and/or hyperactivity [60, 61]. The fact that the three DEX voltage-gated sodium channels *Scn1a*, *Scn4b,* and *Scn8a* show decreased expression in KO mice is not consistent with the increased excitability observed in L5 cPNs. In contrast, five out of the six decreased Kcn potassium channel genes are consistent with cortical hyperexcitability and associated behaviors, as potassium channel loss-of-functions are known cell-autonomous inducers for increased excitability and epilepsy [62, 63]. Nevertheless, it should be noted that our bulk RNAseq cannot determine how gene dysregulation is distributed among cell types. Single-cell RNAseq analyses will be instrumental in determining if and which specific genes can account for hyperexcitability of each cPN type, and in particular in L5 versus L2/3.

Concerning increased Ih current density in L5 cPNs, a likely candidate gene from our RNA-seq is *Trip8b*. *Trip8b* is a critical regulator of the membrane localization of HCN1 channels that induce Ih current density [64, 65]. Independently of its mechanism, increased Ih current density tend to decrease intrinsic neuronal excitability, suggesting that this phenotype might represent a compensatory mechanism to reduce neuronal hyperexcitability [22].

The large number of DEX genes that can possibly account, either alone or in synergy, for the phenotypes observed in *Neurod2* null mice, renders functional rescue experiments impractical, which is expected when studying a transcription factor [53, 66]. Furthermore, while our RNAseq was performed at P30, our results indicate that some cortical phenotypes of *Neurod2* KO mice arise during embryonic development. Hence, measuring transcriptomic changes in cPN subtypes at prenatal and perinatal stages will provide valuable new insights into the development of the physiopathology in *NEUROD2*-associated NDDs.

For the present study we have generated the first *Neurod2* floxed mouse line using the Tm1a approach [67]. Crossing this new mouse line to the Emx1^IRES*Cre*^ driver line [30] allowed us to demonstrate that forebrain excitatory neurons are the cellular origin for the main neurobehavioral symptoms in the *Neurod2* syndrome. This is an important finding because it shows that the Neurod2-expressing paraventricular nucleus, cerebellum, and thyroid gland, which have been associated with ASDs, are not strongly engaged in the neurobehavioral symptoms described here. Nevertheless, it is worth mentioning that the Emx1^IRES*Cre*^ driver line induces recombination not only in the cortex but also in the hippocampus and in a subset of pallial-derived amygdalar cells [30]. Hence, the contribution of the hippocampus and of pallial-derived amygdalar cells to the *Neurod2* neurobehavioral defects remains to be investigated.

In vitro evidences have indicated a link between *Neurod2* and neuronal activity. Transactivation of Neurod2 target genes is increased by neural activity in cultured cortical neurons [9], and *Neurod2* mRNA expression is regulated by NMDAR activation in excitatory neurons [68]. Using our new Neurod2^flox/flox^ line, it will be interesting to test whether and how *Neurod2* cell-autonomously regulates experience-dependent neuronal and synaptic development in vivo, and how this can relate to NDDs.

This study, from humans to mouse models, identifies *NEUROD2*/*Neurod2* as a new gene linked to ID and ASD, essential for cPN development and function. Global or Emx1-lineage specific *Neurod2* deletion potentiates radial migration, alters the cortical expression of numerous genes related to NDDs and induces ASD-relevant neurobehavioral deficits such as layer disorganization, synaptic, and excitability changes in L5 cPNs, without obvious alterations in neuron morphology, viability, and axonal growth. Our results point to NEUROD2 as a node in a gene regulatory network whose alteration leads to phenotypes including ID and ASD, and to murine *Neurod2* mutants as new candidate animal models of ASD.

## Material, patients and methods

Experimenters were always blinded to genotypes during data analysis (we used a custom-made algorithm to blind/unblind data).

### Animals

Mice (mus musculus) were group housed (2–5 mice/cage) with same-sex littermates on a 12-h light-dark cycle with access to food and water *ad libitum*. *Neurod2* full KO mice were previously described [54]. Initially on a C57BL/6J background, they were backcrossed for 4 generations to 129 Sv background to allow normal survival [69]. For layer 5 cPN morpho-functional analyses, *Neurod2* KO mice were bred with mice expressing eGFP under the Thy-1 promoter (The Jackson Laboratory, 007788, Tg (Thy1- EGFP)MJrs/J). Animal experiments were carried out in accordance with European Communities Council Directives and approved by French ethical committees (Comité d’Ethique pour l’expérimentation animale no. 14; permission number: 62-12112012, Apafis #21683-2019073011285386v4).

For *in utero* electroporations, mice were excluded when the fluorescent cell cohort was not targeted to the somatosensory cortex. For Thy1-GFP and AAV-TurboRFP morphological studies, mice with no fluorescent cells in M1/S1 were excluded. Male mice were used for behavioral experiments, while mice of either sex were used for all other experiments after verification that sex did not affect the observed phenotypes.

### Generation of *Neurod2*^flox/flox^ mice

*Neurod2* Tm1a mice, with a conditional allele based on the ‘knockout-first’ design [67], were generated on a C57BL/6N background using cell clone EPD0422_5_B06 from the Knock Out Mouse Project at UC Davis (http://www.komp.org), in which the only coding exon (exon 2) was flanked by loxP sites (Fig. S10a). *Neurod2*^flox/flox^ mice, or Tm1c mice for *Neurod2* [67], were produced by crossing Tm1a mice with actin-Flippase mice (Jackson Stock N° 003800). For forebrain-deletion experiments, *Neurod2*^flox/flox^ mice were crossed with Emx1^IRES*Cre*^ mice [30].

### Cell cultures

Human Embryonic Kidney 293T cells (HEK293T) cells were maintained at 37 °C in a humidified 5% CO_2_ incubator in Dulbecco’s modified Eagle’s medium (Thermofisher scientific) supplemented with 10% foetal bovine serum, 2 mM GlutaMAX supplement (Thermofisher scientific). P19 cells were maintained at 37 °C in a humidified 5% CO_2_ incubator in MEM α (Minimum Essential Medium α) supplemented with 2.5% foetal bovine serum, 7.5% bovin serum, 2 mM GlutaMAX supplement (Thermofisher scientific), and 1X MEM Non-Essential Amino Acids Solution. HEK and P19 cells were seeded at 100,000 cells per 24 well coverslip for transfection with lipofectamine 2000 (Thermofisher scientific) and fixed 3 days post-transfection.

### Histology and immunostainings

Mice were perfused transcardially with ice-cold 4% paraformaldehyde (in PBS). Brains were removed and post-fixed overnight at 4 °C with the same fixative. Coronal sections were cut at 50 μm thickness for all histological analyses except for dendritic reconstructions (200 μm), using a cryostat (Leica) or a sliding microtome (Microm).

Immunofluorescence experiments were performed as described before [70]. Briefly, free-floating sections were blocked and permeabilized for one hour in a blocking buffer composed of 10% Normal Goat Serum, 0.2% Triton X-100 (Sigma) in PBS. Primary antibodies, diluted in blocking solution and added overnight at 4 °C, were as follows: rabbit anti-NEUROD2 (1:500, Abcam, #ab104430), rabbit anti-TBR1 (1:1000, Abcam, #31940), rat anti-BCL11B (1:100, Abcam, #ab18465), rabbit anti-CUX1 (1:200, Santa Cruz Biotechnology, #sc13024), mouse anti-RORβ (1:200, Perseus Proteomics, #PP-N7927-00), chicken anti-GFP (1:500, Aves, #GFP-1010). For immunocytochemistry we used mouse anti-Tuj1 (1:500, Biolegend, BLE801201). Corresponding fluorescently labeled secondary antibodies (AlexaFluor, Invitrogen) were added for 2 h in blocking solution at room temperature. Hoechst was added in PBS for 10 min, and sections were mounted on microscope slides that were coversliped using Mowiol solution (Sigma).

### Cholera-toxin retrograde tracing

P28-P30 mice under xylazine/ketamine anesthesia received stereotaxic injections of 0.3 µl of cholera toxin subunit B (CT-B, 1 mg/ml; Thermo Fisher Scientific) conjugated with Alexa Fluor 647 in the striatum (AP:+1 mm; ML:+1.8 mm; DV:−2.9 mm from dura) and conjugated with Alexa Fluor 488 in the thalamus (AP:−1.3 mm; ML:+1.15 mm; DV:−3.5 mm from dura) using Bregma coordinates. This allowed retrograde labeling of, respectively, L5 PNs (striatal injection) and L6 PNs (thalamic injection). Another group of animals were injected with Alexa Fluor 488 CT-B in motor cortex (AP: 0.6 mm; ML: 1.3 mm; DV: 0.7 mm). Ten days after injection, animals were trans-cardially perfused with 4% paraformaldehyde, their brains processed and cut 50 μm thick using a sliding microtome (Microm).

### L2/3 neuron labeling for spine analyses

A volume of 100 nl AAV1.hSyn.TurboRFP.WPRE.RBG (105552-AAV1, Addgene) was slowly injected (1 nl/second) in M1 (AP:+0.86 mm; ML:+1.4 mm; DV:−0.32 mm from dura) of P27 or P120 mice, which were sacrificed 7 days later and their brains processes for confocal microscopy.

### In utero electroporations

Timed pregnant *Neurod2* heterozygous females fertilized by heterozygous males (E13.5 or E15.5) were anesthetized with isoflurane (7.5% for induction and 3.5% for surgery). The uterine horns were exposed. A volume of 1–2 µL of DNA plasmid (pCAGGS-RFP, 0.5 μg/μl or pCAGGS-GFP, 1.5 µg/µl; Gephyrin-GFP intrabodies, 0.5 μg/μl; pNeurod1_empty-IRES-GFP, pNeurod1_Neurod2 (wild type versus variants)-IRES-GFP, 0.5 μg/μl) combined with Fast Green (2 mg/ mL, Sigma) was injected into the lateral ventricle of each embryo with a pulled glass capillary and a microinjector (Picospritzer II, General Valve Corporation, Fairfield, NJ, USA). Electroporation was then conducted by discharging a 4000 µF capacitor charged to 27 V for E13.5 and 33 V for E15.5, with a BTX ECM 830 electroporator (BTX Harvard Apparatus, Holliston, MA, USA). Five electric pulses (5 ms duration) were delivered at 950 ms intervals using electrodes. Embryos were collected 5 days post-electroporation (at E18.5), and 80 μm coronal slices cut using a sliding microtome (Microm). Electroporated cortices were imaged with an Apotome microscope (Zeiss) for migration analysis and with a confocal microscope (LSM800, Zeiss) for spine and cell morphological analyses.

### Imaging

For NEUROD2 expression analysis (Fig. 1 and S1), we used a Zeiss LSM 800 confocal microscope with ×20 and ×40 objectives and z stacks with optimal z-steps. For laminar cell distribution in vivo, we used an apotome with a ×10 objective. Three stacks separated by 2.3 μm z-steps centered in the mid depth of each slice were imaged and a maximum intensity projection was generated. For 3D dendritic and somatic reconstructions, neurons were imaged from 200 μm thick sections using the LSM 800 confocal microscope with a ×20 objective and a 0.49 μm z-step. Spine images were acquired as 3D stacks with lateral and z-axis resolution of 100 nm and 380 nm using the LSM 800 confocal microscope, with an oil-immersed ×63 objective by adding a 3.4 digital zoom. For basal dendritic spines, dendritic segments directly distal to the first bifurcation away from the soma were imaged. For apical dendritic spines, dendritic segments within 5–100 μm of the pial surface ending were imaged. For morphological analyses in the IZ, cells were imaged with a ×40 objective (NA 1.4) with a digital zoom of 2 and a z-stack of 0.2 μm. Imaging of P19 and HEK cell cultures was performed at the LSM800 confocal microscope with ×20 and ×40 objectives, respectively. For HEK cell imaging we imaged 15 z-stacks of 0.2 μm.

### Image analyses

All cells, dendrites, and spines analyzed were located in M1 and S1 cortex. Cell counts were performed either manually by one or two independent investigators who were blinded to genotype, or by running Cellpose [71], a generalist, deep learning-based segmentation algorithm. When using Cellpose, for each labeling, cell detection parameters were first manually adjusted and then unbiasedly applied to all images by running the algorithm. The validity of Cellpose counting was demonstrated beforehand by comparing with manual quantifications for several image datasets.

For laminar cell distribution analyses, 3–5 slices per animal were selected in stereotyped rostro-caudal levels. Cortical columns were cropped in stereotyped positions of the somatosensory cortex. For P7 and P30 mice, lower and upper limits of the cropped images were the dorsal border of corpus callosum and the pial surface, respectively. For E15.5 and E18.5 embryos, lower and upper limits were SVZ-IZ boundary and pial surface, respectively. Each cortical column was divided into 20 bins of equal size spanning the cortical thickness using a custom-made algorithm on Fiji. Cells were quantified in each bin, and bin-distribution was defined as the percentage of cells in each bin relative to the total number of cells. The Y coordinate of each cell was also measured to calculate the probability density function, which defines the probability distribution of the cells in the depth of the cortical column. The cumulative distribution function was also calculated and represented to visualize the probability of a given cell to be located below a certain position in the cortical column.

The average thickness of a given layer was defined as the sum of its thickness measured at the left edge, the right edge and the middle of the image. Layer borders were defined by only taking into account strongly fluorescent cells. Layer thickness is represented as relative to the whole cortical thickness. For 3D morphological analyses of neuronal dendrites and somas, neurons were reconstructed three-dimensionally using Imaris software (Bitplane). Digital reconstructions were analyzed with Imaris. For morphological analyses in the IZ, electroporated cells were manually segmented by using the Cellpose GUI interface [71] (1773 cells were segmented, 704, 659, and 410 cells from WT, HET and KO mice respectively). Cell morphology was analyzed by using the VAMPIRE (Visually Aided Morpho-Phenotyping Image Recognition) algorithm [72], which enables the profiling and classification of cells into shape modes based on equidistant points along cell contours. Briefly, the number of coordinates to extract from cell contours was set to 200. To optimize the number of shape modes to use in the model, the inertia value was calculated on 10 separate runs of VAMPIRE analysis at each number of shape modes value. The optimal number of shape modes was determined to be around 20. Further analyses were carried out by using these parameters. Spine density quantifications were performed on maximum intensity projections using NeuronStudio [73]. Analysis of NEUROD2 protein levels in HEK cells was performed on maximum intensity projections with Fiji. Nuclei were selected and mean fluorescence intensity measured. Image analyses were all performed blindly to experimental groups, using a custom-made algorithm for file randomization/de-randomization.

### In vivo trans-cranial two-photon imaging for spine turnover measurements

Four-week-old *Neurod2 KO*; Thy1-GFP mice were ketamine/xylazine anesthetized. The skin over the skull was incised, and the skull was cleaned to ensure adhesion, using activator (10% citric acid). An aluminum bar was then glued and cemented using dental cement (Super-Bond C&B) on the skull, caudally to Bregma, keeping the skull parallel to the bar. The animal was then placed in a stereotaxic frame, attached to the metal bar, and the primary motor cortex was marked (coordinates: +1.0 AP; +2.0 ML). Using a scalpel blade, the skull was gently thinned until less than 20 μm thickness, avoiding any bleeding, breaking or extensive drying of the skull. Dendritic segments that were bright and visible were located and marked on a low magnification z-stack and then imaged at high resolution using a ×20 water immersion objective (1.0 NA, 1.7 mm wd, axial resolution: 0.21 μm; step size: 0.79 μm). After imaging the skin over the skull was stitched and animals received a subcutaneous injection of Carprofen (5 mg/kg) and Buprenorphine (0.3 mg/kg). Three days after the first imaging session, animals were anesthetized and the skin covering the skull was re-opened. Next, the skull was cleaned and new images of each dendritic segment were taken. New and eliminated spines were then determined blindly, comparing images at day 0 and at day 3 on ImageJ, on single z-planes.

### Quantitative reverse transcription-polymerase chain reaction (qRT-PCR)

Total RNAs were extracted either from quickly micro-dissected whole cortices (for developmental expression of *Neurod2*, *n* = 3–4 brains per stage in each condition) or from the pooled motor and somatosensory areas (for confirmation of RNAseq data at P30, *n* = 3 repetitions) using TRIZOL reagent according to manufacturer’s instructions (Life Technology). cDNA was synthesized from 1 μg of total RNA using Quantitect Reverse Transcription Kit and according to manufacturer protocol (Qiagen). RT-PCRs were then carried out using SYBR-Green chemistry (Roche Diagnostics) and Roche amplification technology (Light Cycler 480). PCR primers (Table 2 below) were designed for 12 mouse genes, and for 3 control genes, *Ptgs2* (Prostaglandin-Endoperoxide Synthase 2), Rpl13a (Ribosomal protein L13a), and *Hprt1* (Hypoxanthine Phosphoribosyltransferase 1) for normalization. All primer pairs were optimized to ensure specific amplification of the PCR product and the absence of any primer dimer. Quantitative PCR standard curves were set up for all. Values of fold change represent averages from duplicate measurements for each sample.

**Table 2:** Primer pairs used for qPCR in this study.

Gene	Forward	Reverse
*Neurod2*	AAGCCAGTGTCTCTTCGTGG	GCCTTGGTCATCTTGCGTTT
*Scn4b*	GAACCGAGGCAATACTCAGG	ACGACAGGTACATGGGAAGC
*Kcnk4*	CACTCACTGGCCTGGACAA	GAGCTCCTGGGGAGCAGT
*Scn8a*	CAAGCTGGAGAATGGAGGCA	TAAGAGGGGAGGGAGGCTGT
*Kcnh1*	GGTGAGAATGTTCACAAGCACT	ACTGGGGAAGGATGTCTGAA
*Scn1a*	GGTTTGAGACCTTCATTGTGTTC	TTTTGATCGTCTTTCGCTGA
*Kcnq5*	TACAGGAGCAGCACCGCCAG	CCTTGTTCTTTCTTGGTAGGGC
*Cacna1c*	CCCTTCTTGTGCTCTTCG TC	TTGTGCATCTTTCCCATG AA
*Grin2b*	TGCTGTAGCTGTCTTTGTCTTTG	CTTTGCCGATGGTGAAAGAT
*Htr2a*	CTGCTGGGTTTCCTTGTCAT	GTAAATCCAGACGGCACAGAG
*Cdh8*	GTGACCCTGATATCACTTCCAGT	TCTTCCCATCATCTGCATTG
*Ppia1*	QT00247709	QT00247709
*Rpl13a*	CCCTCCACCCTATGACAAGA	GCCCCAGGTAAGCAAACTT
*Hprt1*	QT00166768	QT00166768

### ChIP-Seq data analysis

We used the Chip-Seq dataset from the study of Bayam et al. [17], who isolated NEUROD2-associated chromatin from mouse E14.5 cortex. The dataset, analyzed until the peak calling step, was accessible from Gene Expression Omnibus (GEO) under the accession number [GEO:GSE67539]. We performed peak annotation by using the R package ChIPSeeker [74]. In the annotatePeak function of ChIPSeeker we set the Region Range of TSS between −1000 and 1000 to assign peaks to genes. We then conducted a GO analysis by using the R package ClusterProfiler [75]. This GO analysis allowed us to identify numerous terms related to neuronal migration.

### Electrophysiological recordings

Coronal slices (250 µm) from 21 to 30 days-old mice were cut with a VT 1000S vibratome (Leica) in ice-cold high-choline artificial cerebro-spinal fluid (ACSF) containing (in mM): 130 choline, 2.5 KCl, 1.25 NaH_2_PO_4_, 7 MgCl_2_, 0.5 CaCl_2_, 25 NaHCO_3_ and 7 glucose at 4 °C. Slices were then maintained at room temperature in oxygenated ACSF containing (in mM): 126 NaCl, 2.5 KCl, 1.2 NaH_2_PO_4_, 1.2 MgCl_2_, 2.4 CaCl_2_, 25 NaHCO_3_, and 11 glucose, to which 250 µM kynurenic acid and 1 mM sodium pyruvate were added. Slices were then transferred one at a time to a submersion recording chamber and were perfused continuously with ACSF warmed to 33 °C at a rate of 2.5–3 ml/min. All solutions were equilibrated with 95% O_2_/5% CO_2_. Neurons were visualized on an upright microscope (Nikon Eclipse FN1) equipped with DIC optic and filter set to visualize EYFP using a ×40 water-immersion objective. Miniature excitatory and inhibitory postsynaptic currents (mEPSCs and mIPSCs, respectively) were recorded in whole-cell configurations in voltage-clamp mode in oxygenated ACSF containing tetrodotoxin (TTX, 1 µM). Patch-clamp electrodes (4–6 MΩ) were filled with an intracellular solution of the following composition (in mM): 120 CsMeSO_4_, 12.3 CsCl, 0.1 CaCl_2_, 1 EGTA, 10 HEPES, 4 MgATP, 0.3 NaGTP, pH adjusted to 7.25 with CsOH and osmolarity adjusted to 270–280 mOsm/L. Cells were kept at −60 mV, the reversal potential for GABAergic events, or −4 mV, the reversal potential for glutamatergic events, for the recordings of mEPSCs and mIPSCs, respectively. In some experiments, picrotoxin (50 µM) and 6-cyano-7-nitroquinoxaline-2,3-dione (CNQX, 10 µM) were applied at the end of the experiment to verify that the currents were indeed GABAergic and glutamatergic, respectively. Access resistance was monitored throughout the experiments with a 5-mV negative step and was found to be constant.

To measure intrinsic properties, we used current-clamp recordings. Glass electrodes (6–9 MΩ) were filled with an internal solution containing the following (mM): 130 KMeSO_4_, 5 KCl, 10 4-(2-hydroxyethyl)-1-piperazineethanesulfonic acid, 2.5 MgATP, 0.3 NaGTP, 0.2 ethyleneglycoltetraacetic acid, 10 phosphocreatine, and 0.3–0.5% biocytin, pH =  7.21. Access resistance ranged between 15 and 22 MΩ, and the results were discarded if the access resistance changed by >20%. TTX, was obtained from Abcam, CNQX from Tocris and picrotoxin and kynurenic acid from Sigma.

Data were collected with a MultiClamp 700B amplifier (Molecular Devices), filtered at 2 kHz, digitized (10 kHz) with a Digidata 1440 A (Molecular Devices) to a personal computer, and acquired using Clampex 10.1 software (PClamp, Axon Instruments, Molecular Devices). Data were analyzed and plotted in clampfit (Molecular Devices, v 10.2). Miniature currents were analyzed with Mini Analysis (Synaptosoft, version 6.0.7).

### RNAseq

Tissue from motor plus somatosensory cortex of P28 mice was cut with a VT 1000S vibratome (Leica), rapidly micro-dissected and frozen at −80 °C (*n* = 3 independent experiments for WT and KO samples, 1–4 mice per sample). Total RNA was purified using spin columns of the RNeasy Mini Kit (Qiagen) according to manufacturer’s protocol. Library preparation was made with the TruSeq mRNA-seq Stranded v2 Kit sample preparation (Illumina) according to manufacturer’s instructions. Finally, libraries were sequenced on a high-output flow cell (400 M clusters) using the NextSeq® 500/550 High Output v2 150 cycles kit (Illumina), in paired-end 75/75nt mode, according to manufacturer’s instructions. 467 548 934 clusters were generated for 71 Gbp sequenced with 75% ≥ Q30. Reads were first trimmed with sickle v1.33 (https://github.com/najoshi/sickle, RRID:SCR_006800) with parameters –l 25 –q 20 and then aligned to mm10 using STAR v2.5.3a [76] to produce BAM alignment files. Multi-mapped reads and reads with more than 0.08 mismatches per pair relative to read length were discarded. Transcriptome assembly were performed with Cufflinks v2.2.1 [77] (RRID:SCR_014597) using the relative UCSC mm10 GTF file. For each sample Cufflinks assembles the RNA-Seq reads into individual transcripts, inferring the splicing structure of the genes and classified them as known or novel. The output GTF files from each of the Cufflinks analysis and the GTF annotation file were sent to Cuffmerge v2.2.1 [77] (RRID:SCR_014597) to amalgamate them into a single unified transcript catalog. The RNA-Seq data discussed in this publication have been deposited in NCBI’s GEO and are accessible through GEO Series accession number GSE110491.

### Differential gene expression analysis

Gene counts were calculated with featureCounts v1.4.6-p4 [78] (RRID:SCR_012919) from a GTF containing known UCSC mm10 genes as well as the novel genes detected by Cufflinks (Cufflinks class code “u”) and alignment files. The R package DESeq2 v1.14.1 [79] (RRID:SCR_000154) was then used to normalize counts and detect the DEX genes (FDR < 0.05). Batch effect between replicates was added in the design formula of DESeqDataSetFromMatrix function to model it in the regression step and subtract it in the differential expression test.

### Gene ontology

Gene ontology enrichment was performed using all of the expressed genes as background. We used DAVID (RRID:SCR_003033) with high stringency parameters, and ClueGo (Cytoscape) with a similar approach. DAVintellectual disability adjusted *p*-values were used for further evaluation.

### RNA-Seq statistics

We assumed that the samples were normally distributed. *P*-values for overlaps were calculated with binomial test using a custom-made R script. *P*-values were subsequently adjusted for multiple comparisons using Benjamini-Hochberg FDR procedure. Two-way permutation test of 1000 was adapted to validate the overlaps. We randomized the DEX gene sets by randomly selecting a same number of genes from RNA-seq expressed genes and subsequently calculating the overlap *P*-values. Moreover, we adapted a permutation test to evaluate the detected DEX genes, randomizing 1000 times the RNA-seq data and recalculating the DEX genes. Analysis for RNA-seq was performed using custom-made R scripts implementing functions and adapting statistical designs comprised in the libraries used. The heatmap and Volcano plots for gene expression were performed from the gene overlap file using scripts written on R.

### Coexpression network analysis in humans

RPKM data from [51] was obtained from 11 human neocortical areas in all developmental time points and genes corresponding to module 37 were selected. We performed pairwise Pearson correlation in R for all selected genes on (log2 + 1) transformed RPKM and calculated degree centrality for each gene when correlation was at least 0.70. Degree centrality and network visualization were performed with Cytoscape [80]. We colored genes according to SFARI score (release 12-05-2019). We also manually added a circle with 9 cells on all genes that are at least 0.7 correlated with *NEUROD2* and on other genes that are disorder associated. Each cell corresponds to a neuropsychiatric trait/disorder, and there is a color/position legend at the bottom of the figure. The cell is painted if (a) the gene is reported as a genome-wide significant hit in the original GWAS publication (the 9 GWASs we used are found in File S1 sheet “GWAS_NEUROD2_GeneModule”) and (b) if the gene is significant using MAGMA and correcting for Bonferroni. For (b) we used the complete summary stats for each GWAS to calculate a gene-wise *P*-value for enrichment in SNP-heritability. We considered a gene significant if it surpasses P*19120 < 0.05.

### Behavior

All behavioral tests were performed with age-matched male mice that were 8–14-weeks old depending on the timeline for each test. They were done according to the European Union and national recommendations for animal experimentation. The experimenter was blind to the genotypes.

#### Open field test

The test was performed in a 40 × 40 cm square arena with indirect illumination of 100 lux. Mouse movements were video-tracked using Smart 3.0 software (Panlab, Harvard apparatus) for one hour. Total distance traveled, time in center (exclusion of a 5 cm border arena), resting time, and mean velocity were measured. The open-field arena was cleaned and wiped with H20 and 70% ethanol between each mouse (WT: *n* = 16; HET: *n* = 15; KO: *n* = 15).

#### Stereotypic behavior

During the first 10 min of the open-field test, the number of rearing and circling were measured manually. Both on-wall and off-wall rearing were counted. An on-wall rearing event was counted when both forepaws were apposed to the wall. An off-wall rearing event was counted when both front paws had left the floor. A complete 360-degree turn of nose angle with respect to the mouse body center was counted as one circling event (WT: *n* = 16; HET: *n* = 15; KO: *n* = 15).

#### New object recognition test

The arena used for the novel object recognition test was the same used for the open-field test. The arena was cleaned and wiped with 70% ethanol between each mouse. For habituation, the tested mouse was placed in the arena and allowed to explore for 10 min. Following habituation, two identical objects (50 ml orange corning tube) were placed in opposite corners of the arena, 10 cm from the sidewalls. The tested mouse was placed in the center of the arena, and allowed to explore the arena for 10 min. After 24 h, one object was replaced with another novel object, which was of similar size but differed in shape and color with the previous object (white and blue LEGO® bricks). The test mouse was placed in the center, and allowed to explore the arena and the two objects (a new and an “old” familiar object) for 10 min. The movement of the mice was video-tracked with Smart 3.0 software. Time in each proximal area (nose located in a 2 cm area around the object) was measured (WT: *n* = 16; HET: *n* = 15; KO: *n* = 15).

#### Three-chamber test

The three-chamber apparatus consisted of a Plexiglas box (60 × 40 cm, each chamber being 20 × 40 cm) with removable floor and partitions dividing the box into three chambers with 5-cm large openings between chambers. Test mice were housed individually the day before the test. The task was carried out in five trials of 5 min each. After each trial, the mouse was returned to his home cage for 15 min. The three-chambers apparatus was cleaned and wiped with 70% ethanol between each trial.

In the first trial, a test mouse was placed in the center of the three-chamber unit, where two empty wire cages were located in the left and right chambers to habituate the test mouse. The mouse was allowed to freely explore each chamber. The mouse was video-tracked for 5 minutes with Smart 3.0 software. In the second trial, an 8-week old C57Bl/6J mouse (M1) was placed randomly in one of the two-wire cages to avoid any place preference. The second wire cage remained empty (E). The test mouse was placed in the center, and allowed to freely explore the chamber for 5 min. In the following two trials, the same mouse M1 was again placed in one of the wire cages, and the test mouse was placed in the center and allowed to explore each chamber. The goal of these two trials was to familiarize the test mouse with M1. In the last 5-min session, a new 8-week-old C57Bl/6J mouse (M2) was placed in the second wire cage. Thus, the test mouse had the choice between a now familiar mouse (M1) and a new, stranger mouse (M2). Time spent in each chamber and time spent within a 5-cm square proximal to each wire cage with the nose towards the cage (that we called investigation time) were measured (WT: *n* = 19; HET: *n* = 22; KO: *n* = 18).

### In vitro *NEUROD2* pathogenicity test

P19 cells culture and transfection were performed with modification from Farah et al. [43]. P19 mouse carcinoma cells were cultured in MEMα with 7.5% bovine serum and 2.5% fetal bovine serum (HyClone) containing Glutamax and essential amino acids (Gibco), and maintained subconfluent prior to transfection. For lipofectamine 2000 (Invitrogen) transfections, cells were plated on glass coverslips in 24-well dishes at a concentration of 50,000 cells per well and transfected 24 h later following manufacturer’s instructions. Three plasmids were co-transfected in each condition: pCAG-Cre, pCALNL-DsRed, and pCALNL-*NEUROD2, NEUROD2* being either the WT form or any of the patients’ variants. Cells were fixed 3 days post-transfection, immunostained for β3-tubulin (BioLegend BLE801201, 1:1000), counterstained with Hoechst solution (Molecular Probes) and mounted. For each condition we analyzed 3–4 independent experiments with 3 coverslips each.

### Consent and human ethics approval

All subjects or their legal representatives gave written informed consent for the study. Except for family 3 and 4 for which ﻿the study was approved by the Institutional Review Board (IRB) at Yale University, studies of other families in the present work used unlinked anonymized data and were performed in accordance with the Declaration of Helsinki protocols and approved by the Dutch, USA and Belgian ethics committees.

### Patients

The study includes nine patients from seven unrelated families in the Netherlands, USA, Mexico, Canada, and Belgium. All patients or their legal representatives gave written informed consent for the study. Except for Family 3 and 4 for which ﻿the study was approved by the IRB at Yale University, studies of other families in the present work used unlinked anonymized data and were performed in accordance with the Declaration of Helsinki protocols and approved by the Dutch, USA and Belgian ethics committees. Patients were identified in centers where High-throughput Sequencing (whole-exome sequencing and whole-genome sequencing) is being used to identify, or accurately characterize, genomic variants in individuals with developmental brain abnormalities, mental retardation, epilepsy and ASD. Clinical information, brain MRI, and blood were obtained after informed consent. DNA from subjects was extracted from peripheral blood lymphocytes by standard extraction procedures and used for Sanger confirmation of high-throughput sequencing.

### Statistics

All statistical tests are described in the figure legends. Statistical methods to predetermine sample size were not used; sample sizes were estimated based on previous studies using the techniques described. Unless otherwise stated, all values represent the averages of independent experiments ±SEM. Statistical significance for comparisons with one variable was determined by Student’s *t*-test using two-tailed distribution for two normally distributed groups, and by Mann-Whitney test when distributions were not normal. Significance of multiple groups was determined by Kruskal–Wallis, one-way, two-way, or two-way repeated measure ANOVA followed by Bonferroni’s post hoc test as indicated in figure legends.

In two-way ANOVA, the three assumptions for sampling data were tested: normality, homoscedasticity, independence. To test the assumption of residuals normality and homoscedasticity, Shapiro–Wilk test and Levene’s test were performed, respectively. If the assumption of normality was violated, a permutation test for a two-way ANOVA was used.

In particular, to statistically analyze the laminar distribution of the cells within bins (Fig. 2), we first checked if the variables were spatially autocorrelated in order to eliminate the effects of nearby spatial units. The purpose was to meet one of the conditions of two-way ANOVA: independence. We used spatial weights matrix and Moran’s I index to detect spatial autocorrelation. We chose a contiguity matrix as a spatial weights matrix. If one bin was connected with another bin, the corresponding matrix was assigned the value 1, otherwise assigned the value 0. Then, the weights matrix was normalized by dividing each matrix line by its total. If the Moran’s test was not significant, the residuals in the traditional two-way ANOVA were not significantly spatially autocorrelated so a traditional two-way ANOVA could be performed. In the opposite situation, we filtered out the influence of spatial autocorrelation by applying the spatial lag model. The spatial autocorrelation parameter ρ in spatial lag model was estimated by using a maximum likehood method. In spatial lag model, the spatial autocorrelation in residuals was eliminated and therefore spatially adjusted two-way ANOVA can be performed. RNA-seq statistics are described above. Differences were considered significant when *P* < 0.05. All statistical analyses were performed with Prism 6 software (Graphpad) or RStudio.

## Supplementary information

Figure S1

Figure S2

Figure S3

Figure S4

Figure S5

Figure S6

Figure S7

Figure S8

Figure S9

Figure S10

Figure S11

Figure S12

Figure S13

File S1

Video S1

Supplementary Legends
