# Correction: Disruption of *NEUROD2* causes a neurodevelopmental syndrome with autistic features via cell-autonomous defects in forebrain glutamatergic neurons

**DOI:** 10.1038/s41380-021-01234-7

**Published:** 2021-07-19

**Authors:** Karen Runge, Rémi Mathieu, Stéphane Bugeon, Sahra Lafi, Corinne Beurrier, Surajit Sahu, Fabienne Schaller, Arthur Loubat, Leonard Herault, Stéphane Gaillard, Emilie Pallesi-Pocachard, Aurélie Montheil, Andreas Bosio, Jill A. Rosenfeld, Eva Hudson, Kristin Lindstrom, Saadet Mercimek-Andrews, Lauren Jeffries, Arie van Haeringen, Olivier Vanakker, Audrey Van Hecke, Dina Amrom, Sebastien Küry, Chana Ratner, Reena Jethva, Candace Gamble, Bernard Jacq, Laurent Fasano, Gabriel Santpere, Belen Lorente-Galdos, Nenad Sestan, Antoinette Gelot, Sylvie Giacuzz, Sandra Goebbels, Alfonso Represa, Carlos Cardoso, Harold Cremer, Antoine de Chevigny

**Affiliations:** 1 INMED, INSERM, Aix-Marseille University, Marseille, France; 2 IBDM, Aix-Marseille University, CNRS, UMR, Marseille, France; 3 TAGC INSERM U1090, Aix-Marseille University, Marseille, France; 4 Phenotype Expertise, 5 Boulevard du Maréchal Koenig, Marseille, France; 5 Miltenyi Biotec, Bergisch-Gladbach, Germany; 6 Baylor College of Medicine, Houston, TX USA; 7 Cook Children’s Clinical Genetics, Fort Worth, TX USA; 8 Division of genetics and Metabolism, Phoenix Children’s Hospital, Phoenix, AZ USA; 9 Department of Medical Genetics, Faculty of Medicine & Dentistry, University of Alberta, Edmonton, AB Canada; 10 Pediatric Genomics Discovery Program, Department of Pediatrics, Yale University School of Medicine, New Haven, CT USA; 11 Department of Clinical Genetics, Leiden University Medical Center, Leiden, Netherlands; 12 Center for Medical Genetics, Department of Biomolecular Medicine, Ghent University and Ghent University Hospital, Ghent, Belgium; 13 Department of Neurology, Queen Fabiola Children’s University Hospital, Brussels, Belgium; 14 Université Libre de Bruxelles (ULB), Brussels, Belgium; 15 Neuropediatric Unit, Kannerklinik, Centre Hospitalier de Luxembourg, Luxembourg, Grand-Duchy of Luxembourg, Luxembourg, Luxembourg; 16 Centre Hospitalier Universitaire de Nantes, Service de Génétique Médicale, Nantes, France; 17 INSERM, CNRS, UNIV Nantes, l’institut du thorax, Nantes, France; 18 Hackensack University Medical Center, Hackensack, NJ USA; 19 Department of Neuroscience and Kavli Institute for Neuroscience, Yale School of Medicine, New Haven, CT USA; 20 Trousseau Hospital, Paris, France; 21 Max-Planck-Institute of Experimental Medicine, Goettingen, Germany

**Keywords:** Neuroscience, Autism spectrum disorders

Correction to: *Mol Psychiatry* 10.1038/s41380-021-01179-x, published online 29 June 2021

The article “Disruption of *NEUROD2* causes a neurodevelopmental syndrome with autistic features via cell-autonomous defects in forebrain glutamatergic neurons”, written by Karen Runge, Rémi Mathieu, Stéphane Bugeon, Sahra Lafi, Corinne Beurrier, Surajit Sahu, Fabienne Schaller, Arthur Loubat, Leonard Herault, Stéphane Gaillard, Emilie Pallesi-Pocachard, Aurélie Montheil, Andreas Bosio, Jill A. Rosenfeld, Eva Hudson, Kristin Lindstrom, Saadet Mercimek-Andrews, Lauren Jeffries, Arie van Haeringen, Olivier Vanakker, Audrey Van Hecke, Dina Amrom, Sebastien Küry, Chana Ratner, Reena Jethva, Candace Gamble, Bernard Jacq, Laurent Fasano, Gabriel Santpere, Belen Lorente-Galdos, Nenad Sestan, Antoinette Gelot, Sylvie Giacuzz, Sandra Goebbels, Alfonso Represa, Carlos Cardoso, Harold Cremer & Antoine de Chevigny, was originally published electronically on the publisher’s internet portal on 29 June 2021 without open access. With the author(s)’ decision to opt for Open Choice the copyright of the article changed on 6 July 2021 to © The Author(s) 2021 and the article is forthwith distributed under a Creative Commons Attribution 4.0 International License, which permits use, sharing, adaptation, distribution and reproduction in any medium or format, as long as you give appropriate credit to the original author(s) and the source, provide a link to the Creative Commons licence, and indicate if changes were made. The images or other third party material in this article are included in the article’s Creative Commons licence, unless indicated otherwise in a credit line to the material. If material is not included in the article’s Creative Commons licence and your intended use is not permitted by statutory regulation or exceeds the permitted use, you will need to obtain permission directly from the copyright holder. To view a copy of this licence, visit http://creativecommons.org/licenses/by/4.0.

