# Supplementary figures and images for: Disruption of NEUROD2 causes a neurodevelopmental syndrome with autistic features via cell-autonomous defects in forebrain glutamatergic neurons
Source: Mol Psychiatry. 2021 Jun 29;26(11):6125–48. doi: 10.1038/s41380-021-01179-x (PMC8760061; doi:10.1038/s41380-021-01179-x)

**S1****a**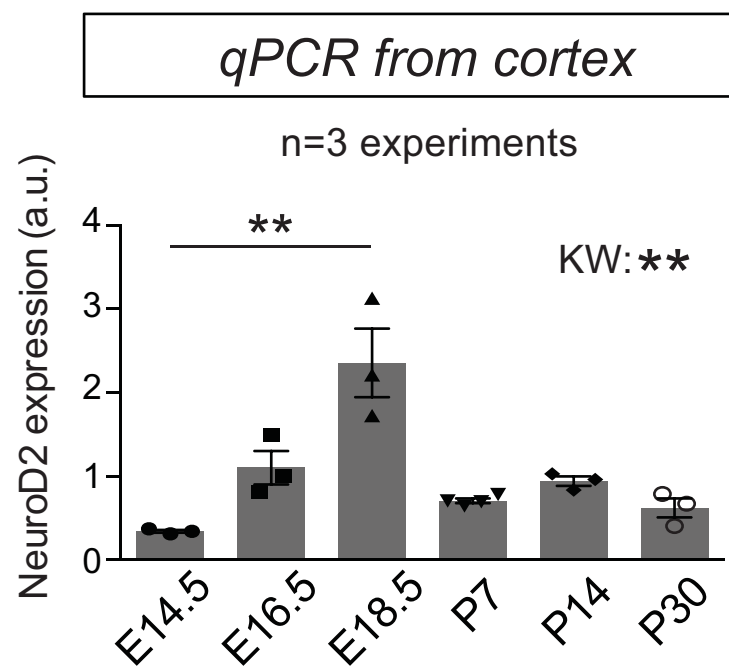**b**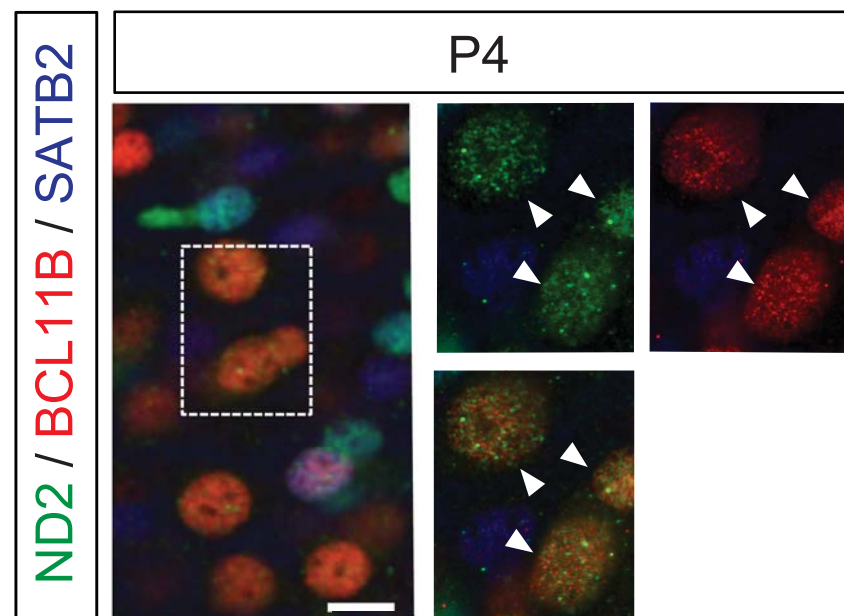**c**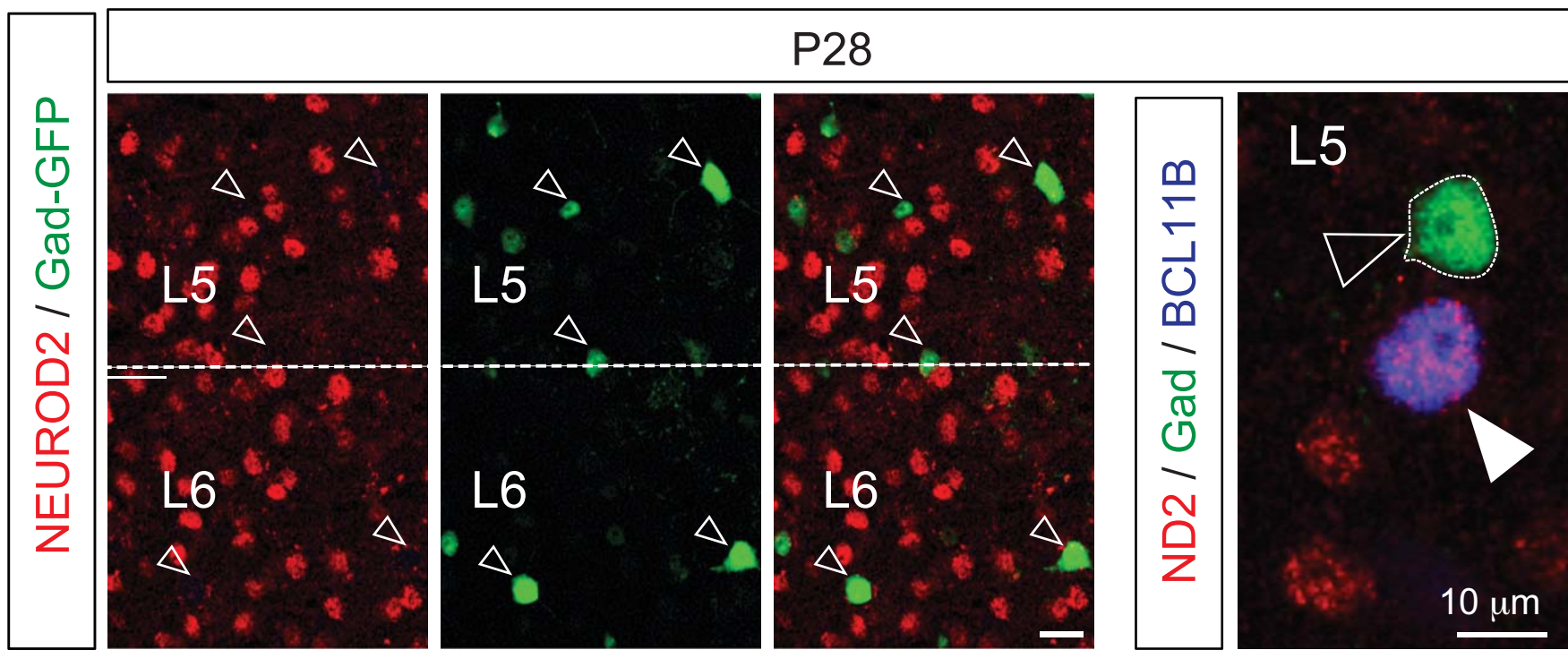

Supplement: Supplementary file 1 — Figure S1 [file 41380_2021_1179_MOESM1_ESM.pdf]

## CALLOSAL SIZE

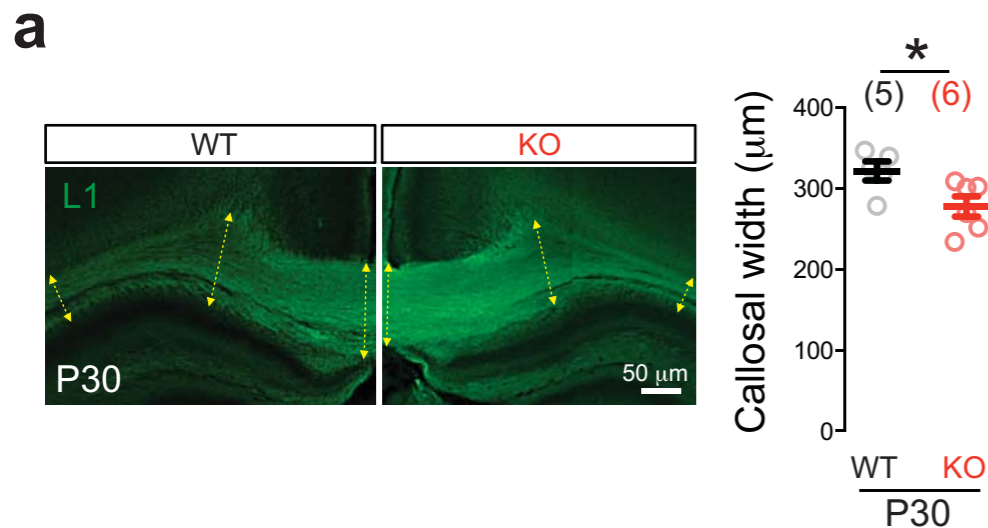**b**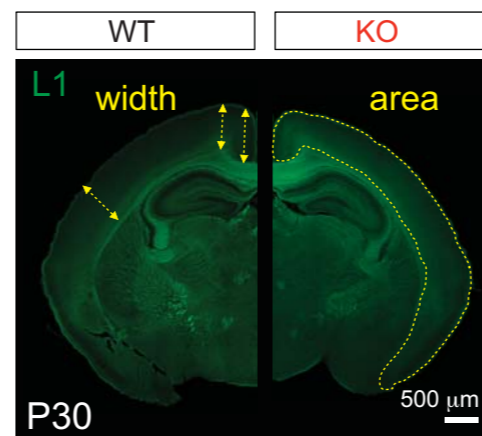

## CORTICAL SIZE

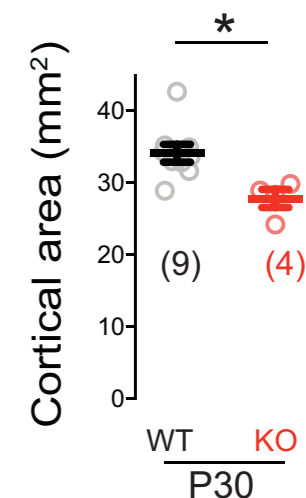

## AXONAL OUTPUTS

**c**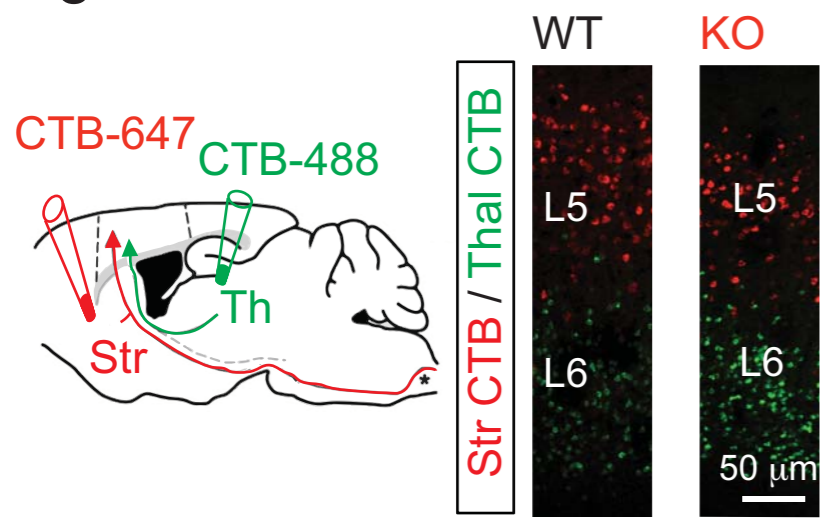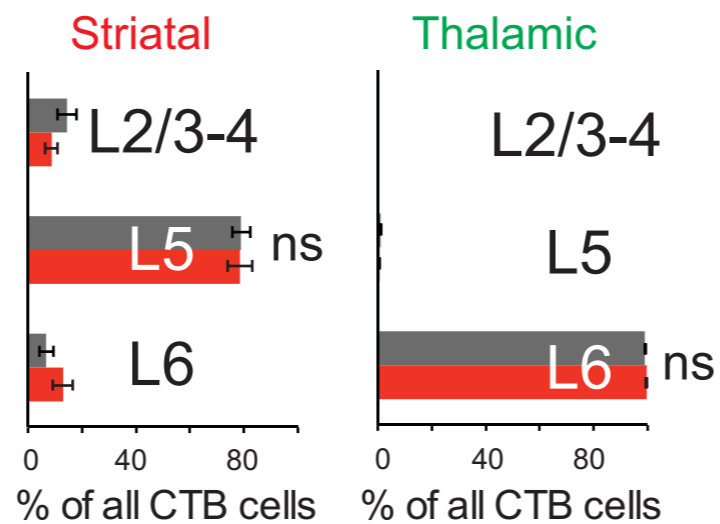**d**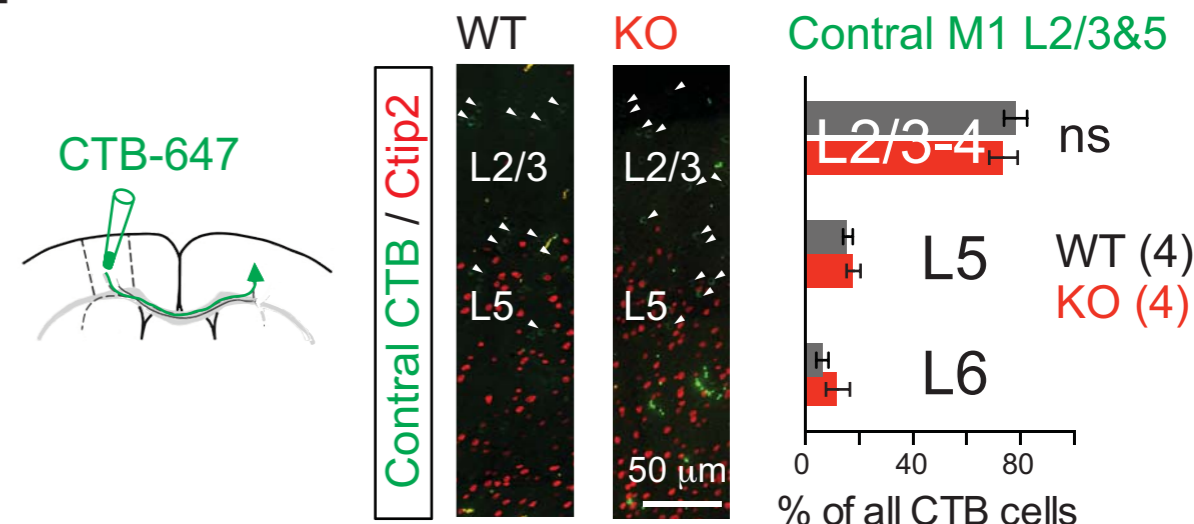

Supplement: Supplementary file 2 — Figure S2 [file 41380_2021_1179_MOESM2_ESM.pdf]

a

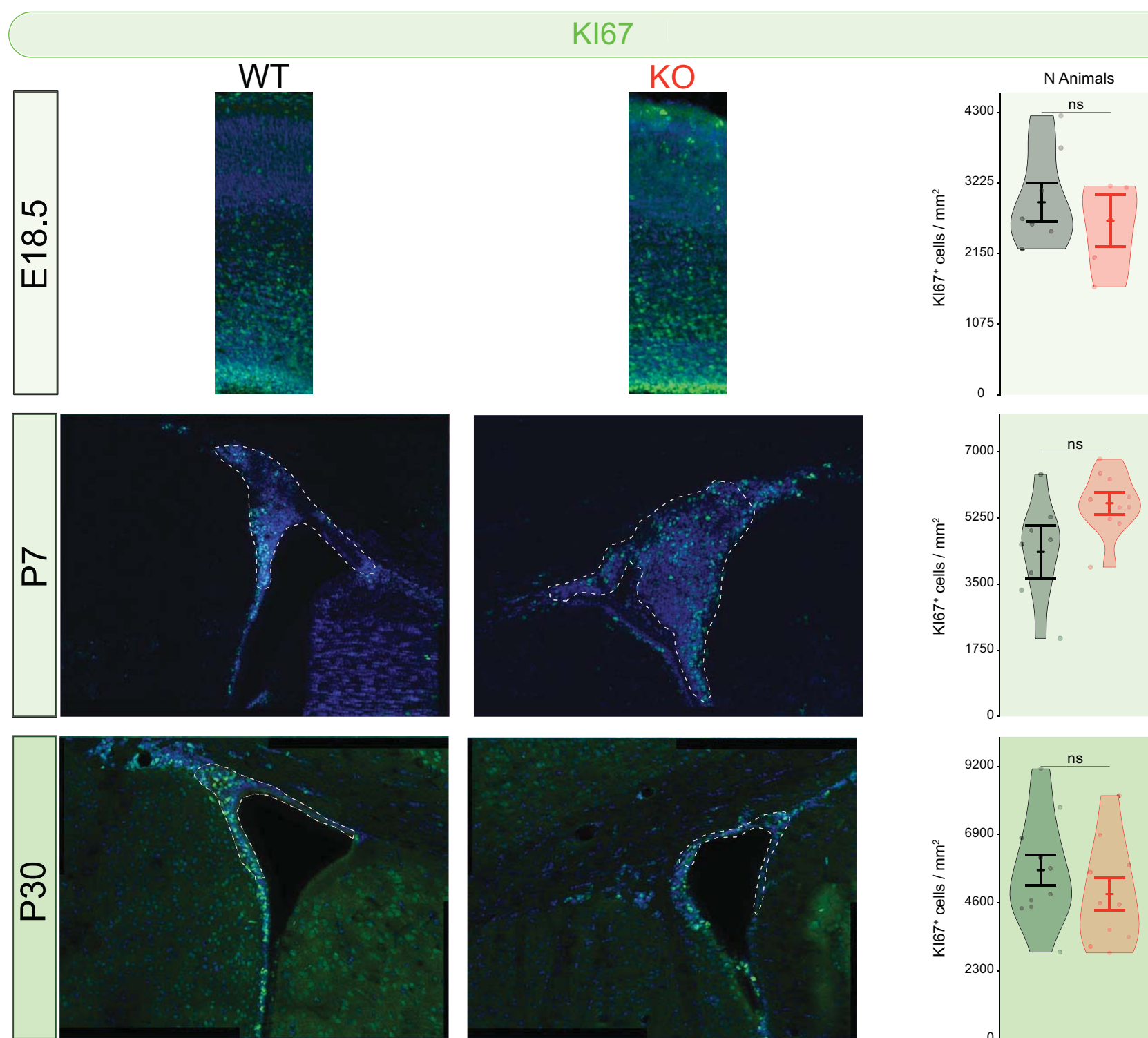

b

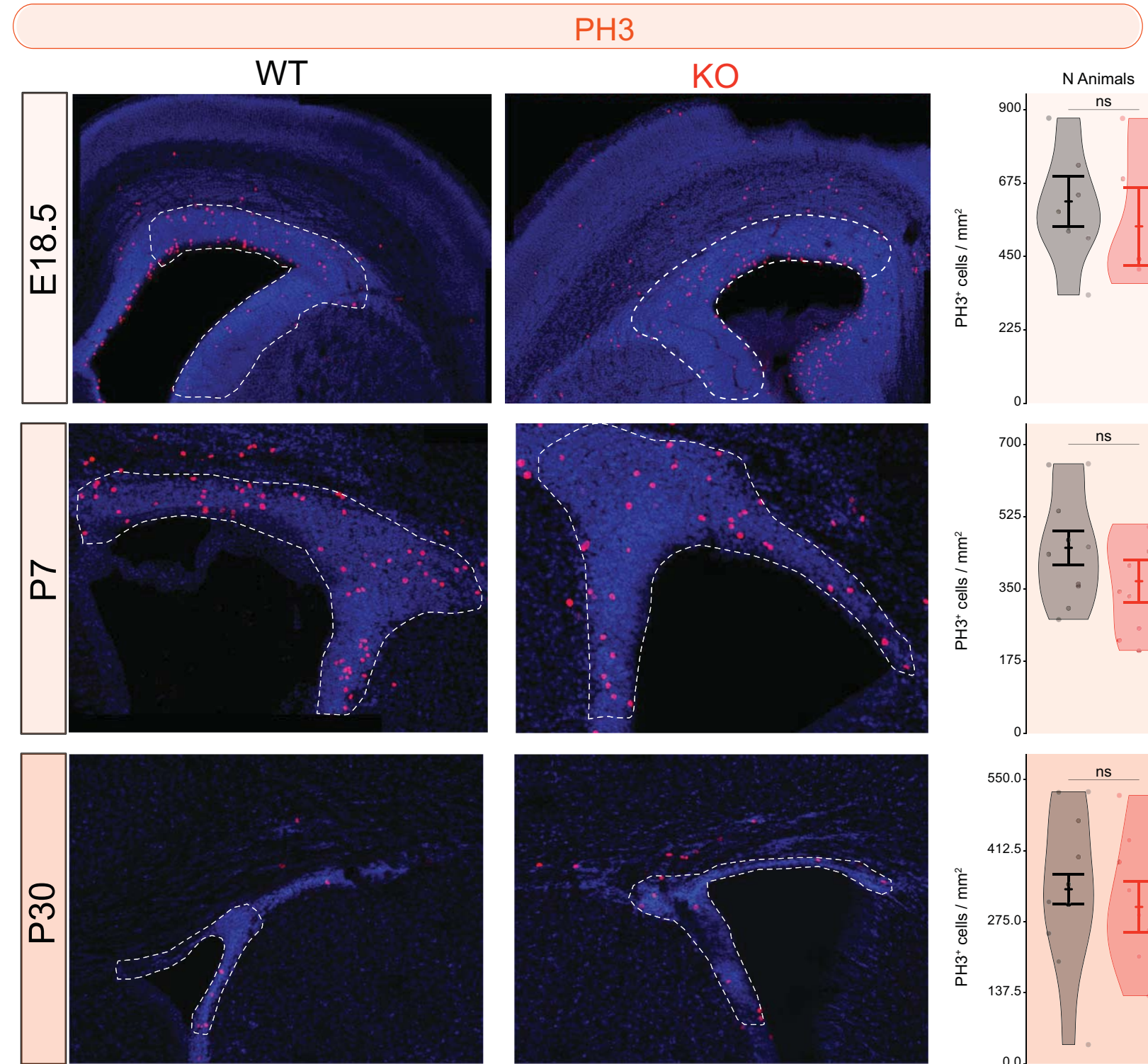

c

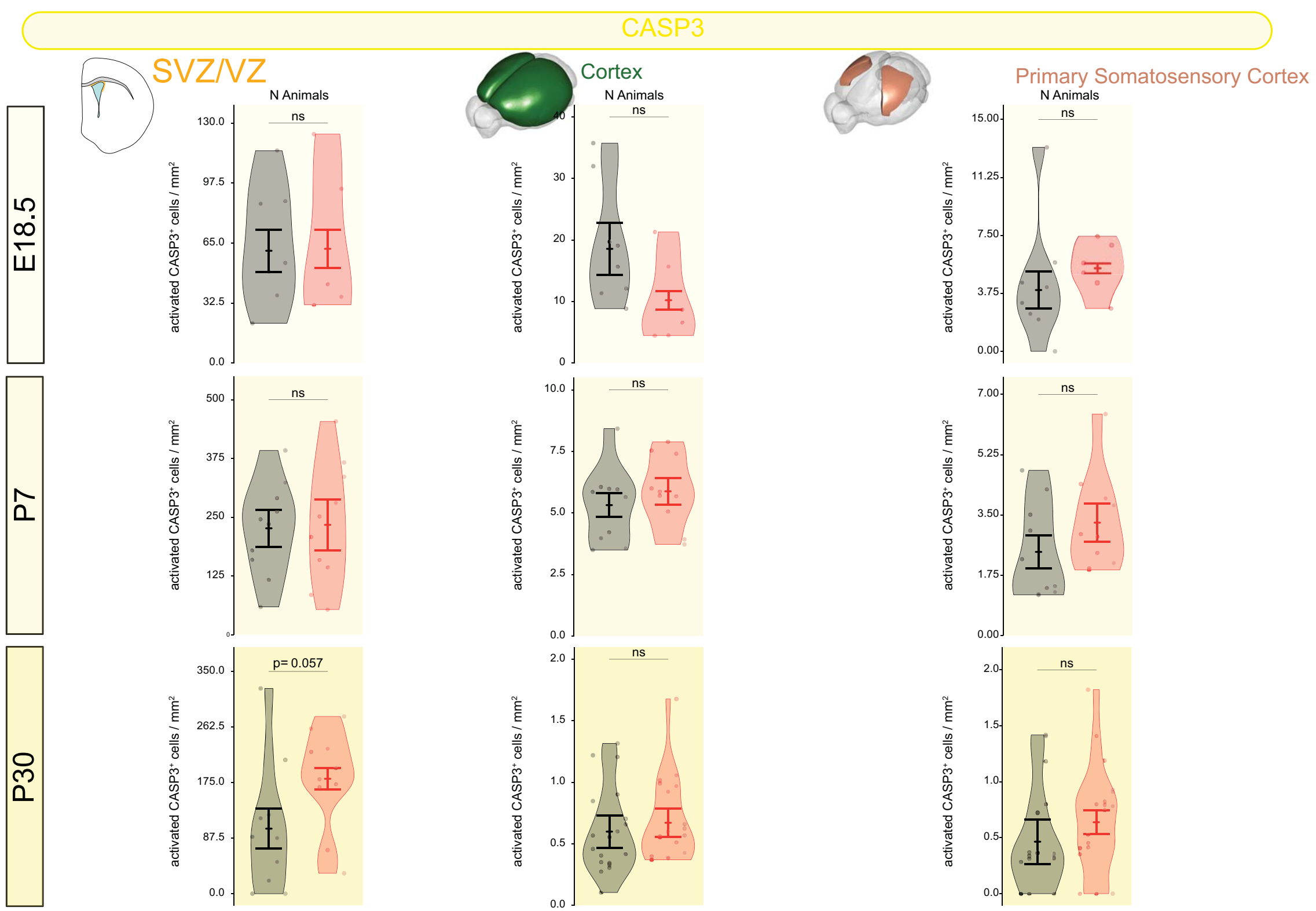

Supplement: Supplementary file 3 — Figure S3 [file 41380_2021_1179_MOESM3_ESM.pdf]

**a**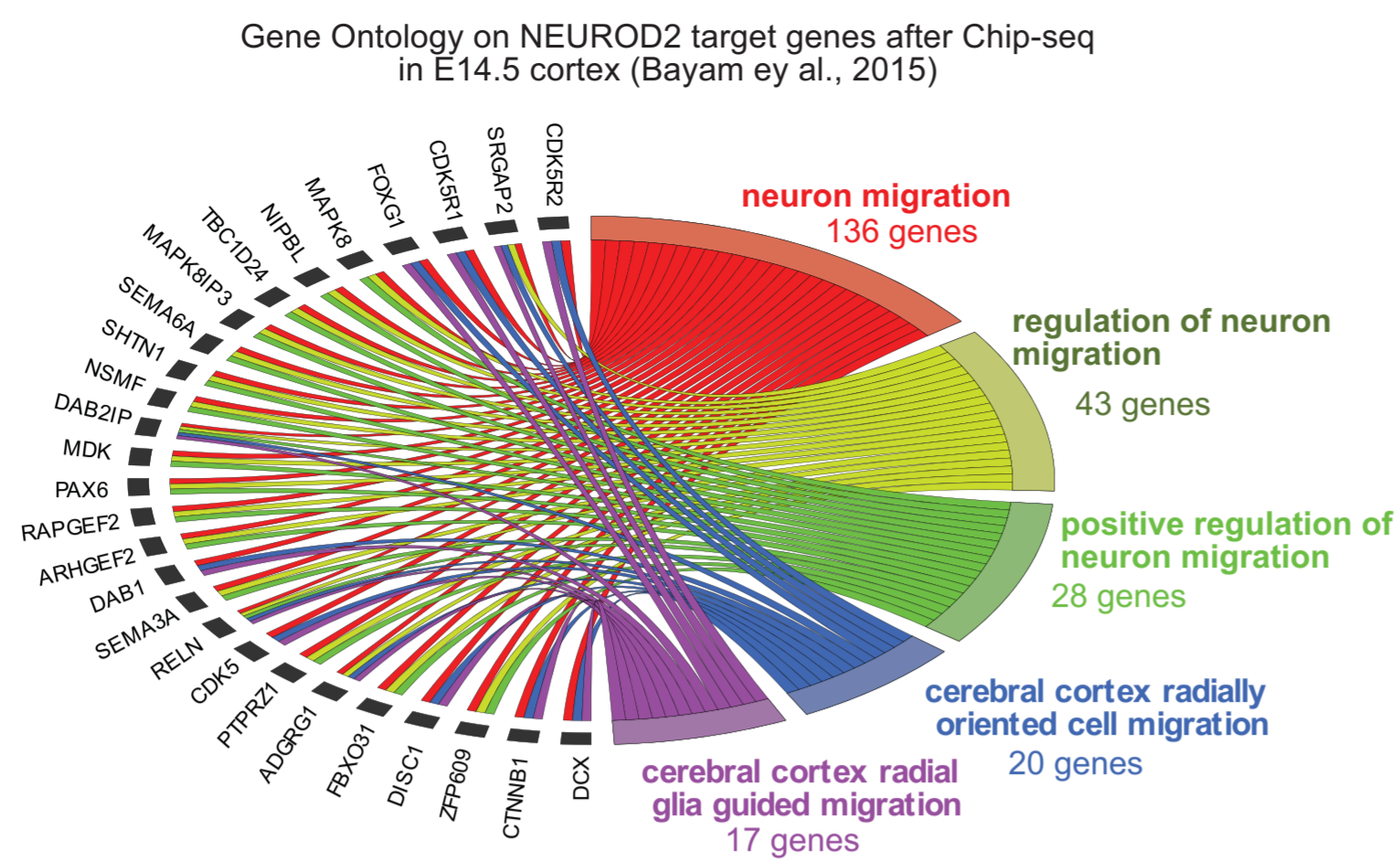**b**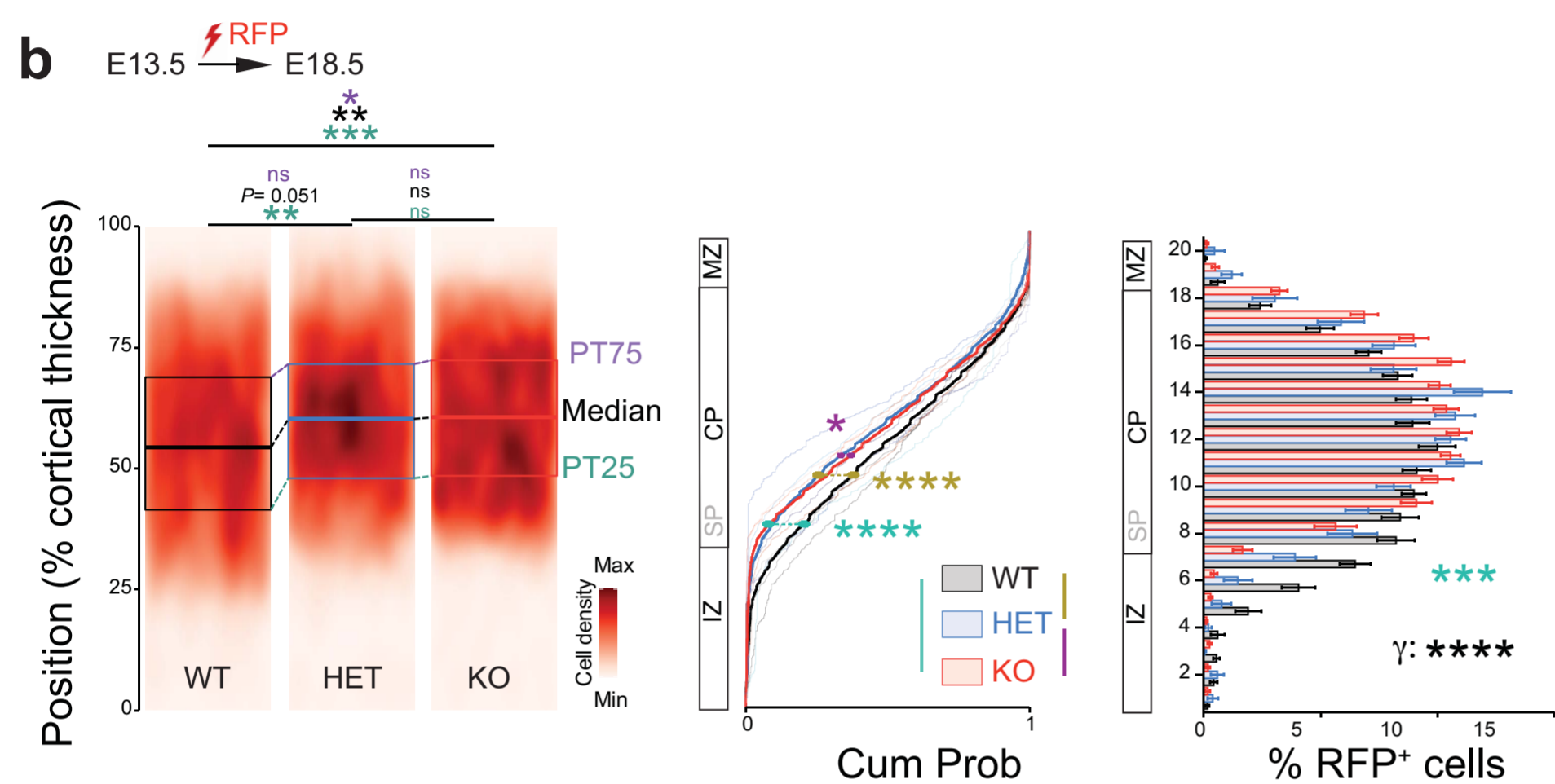**c**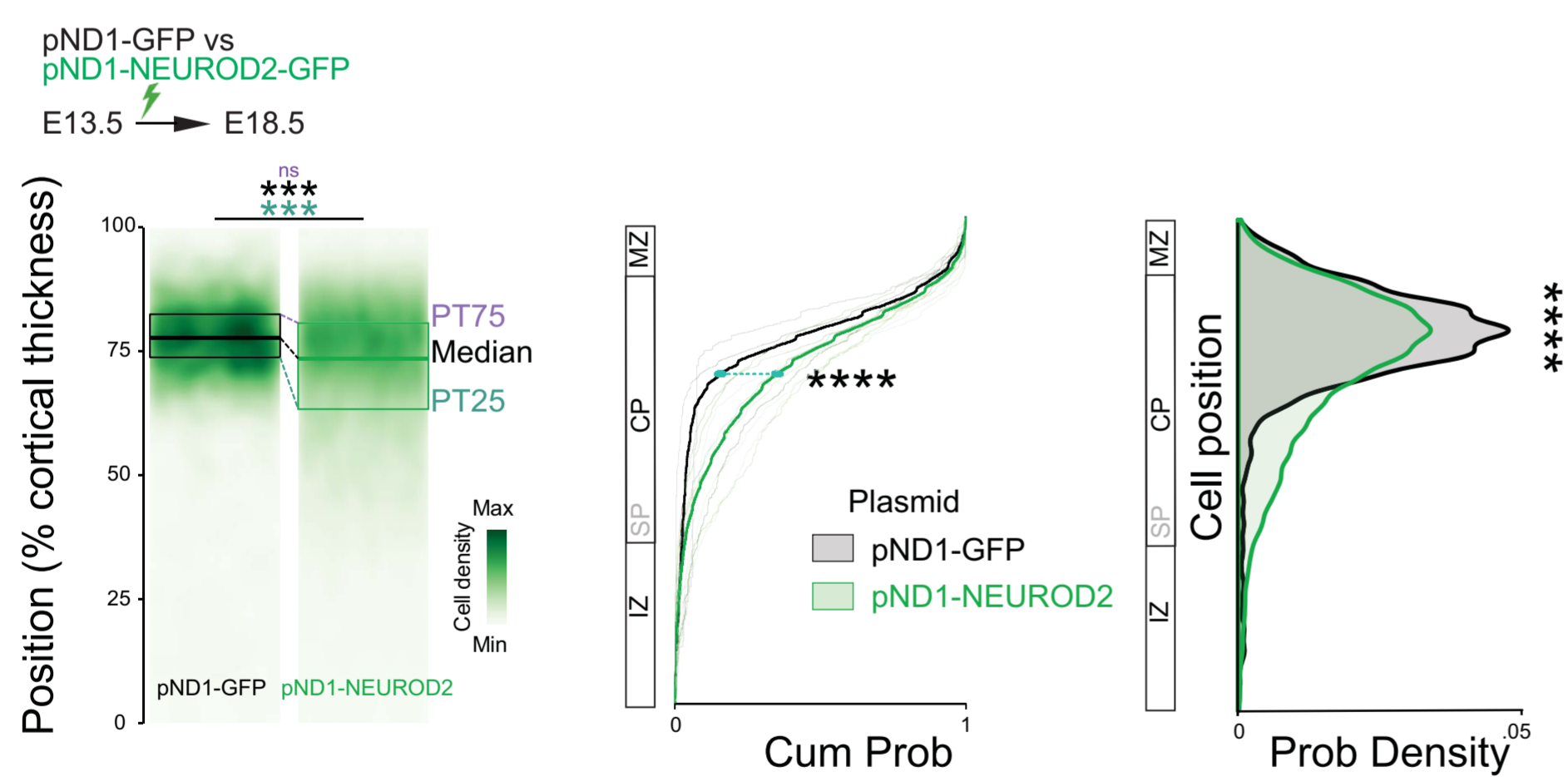**g**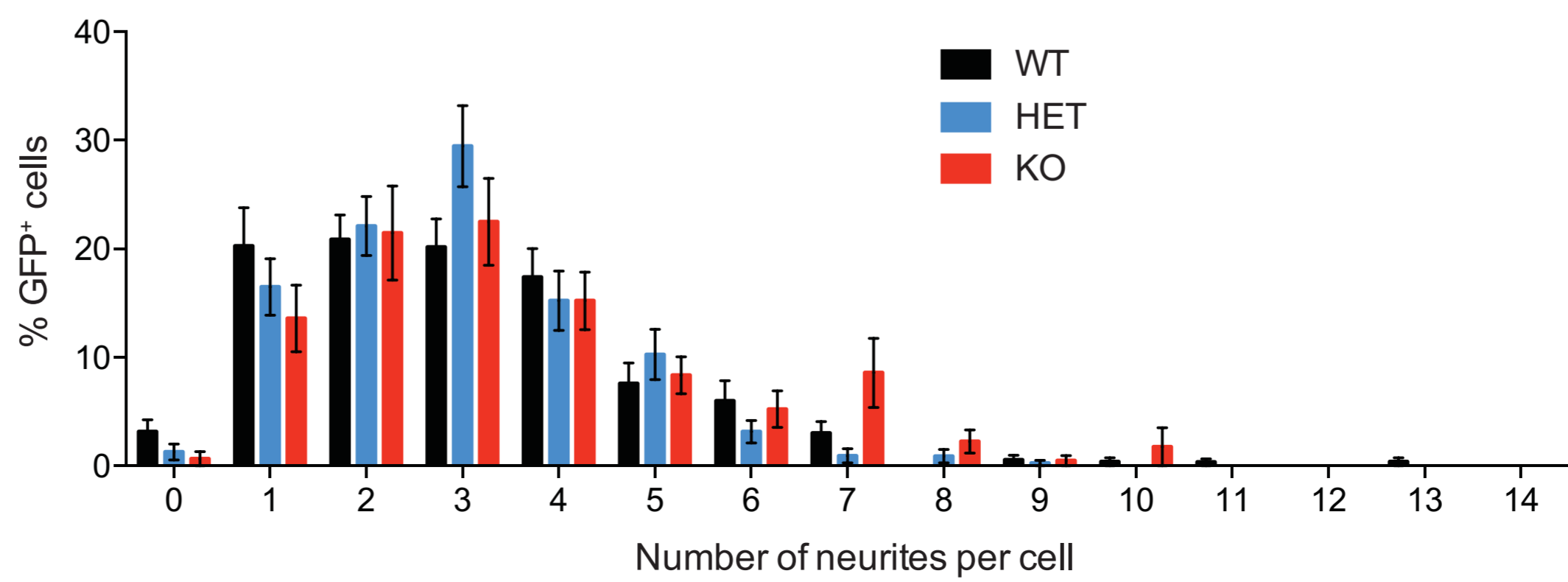**d**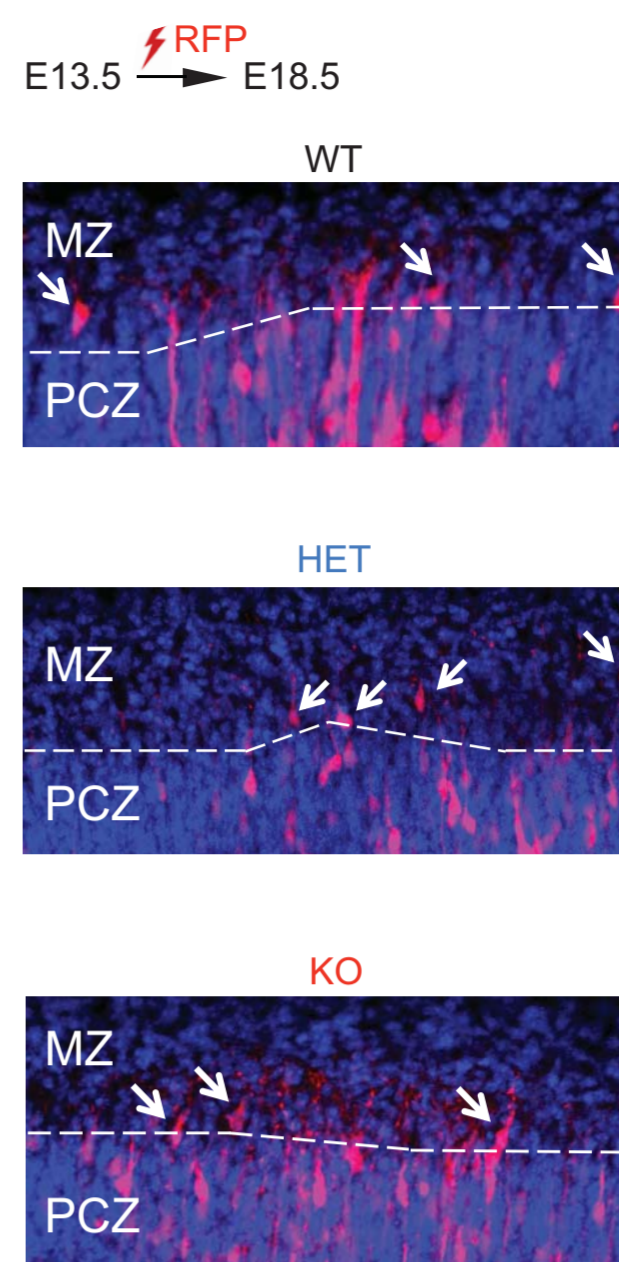**e**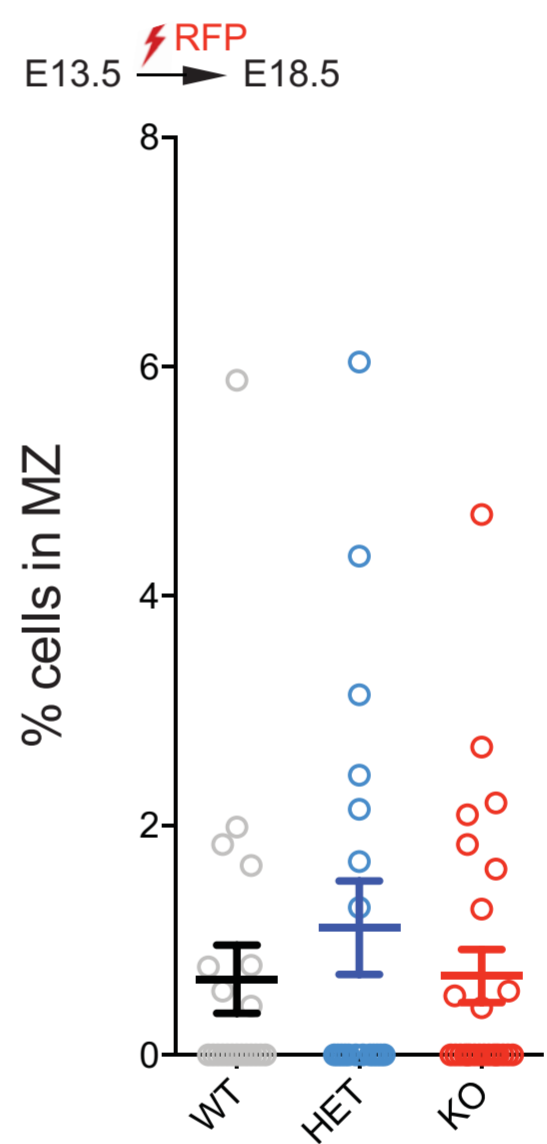**f**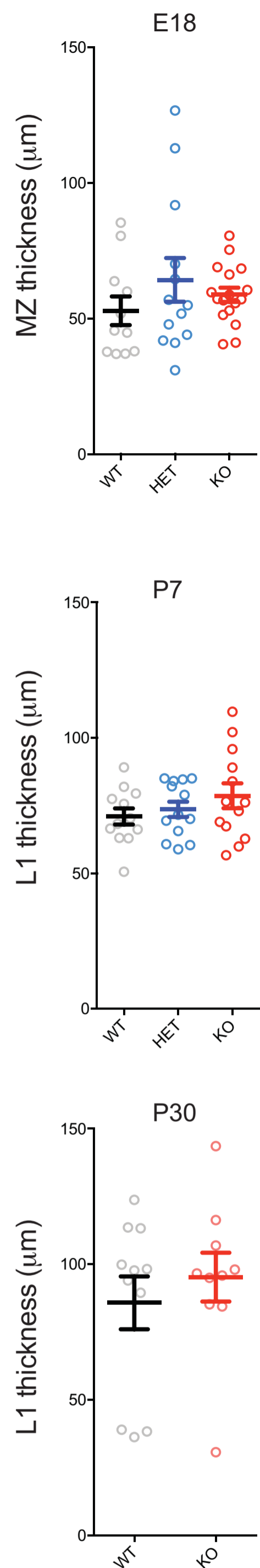

Supplement: Supplementary file 4 — Figure S4 [file 41380_2021_1179_MOESM4_ESM.pdf]

**S6****a**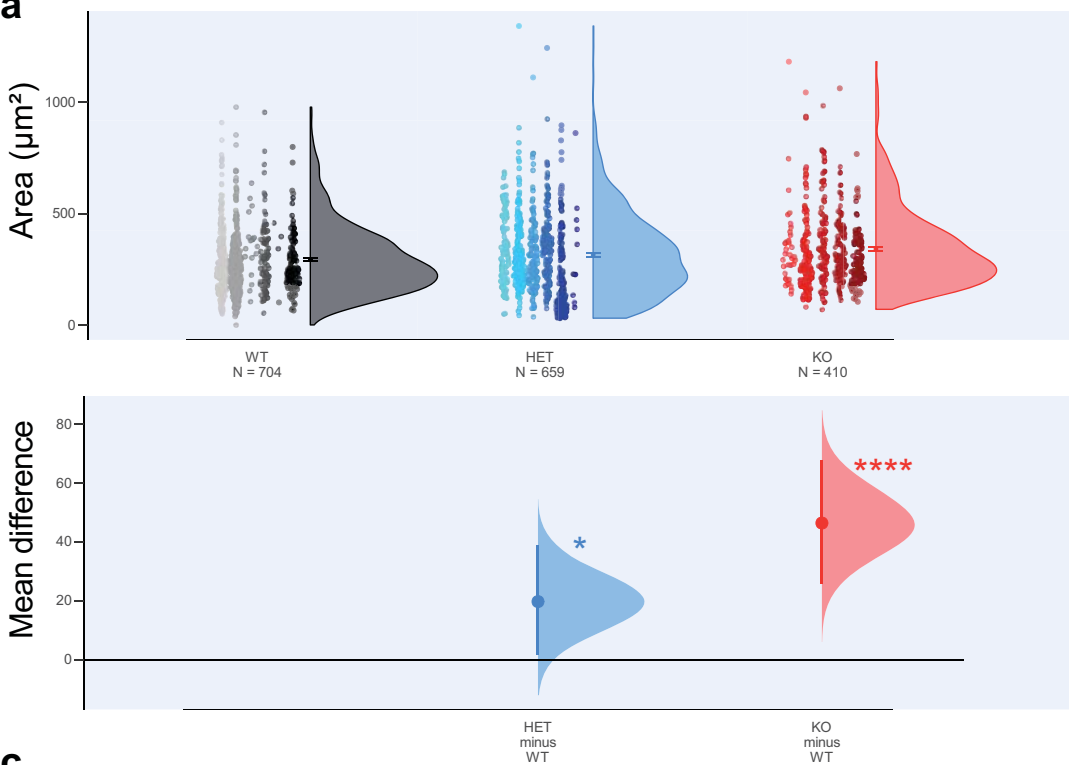**b**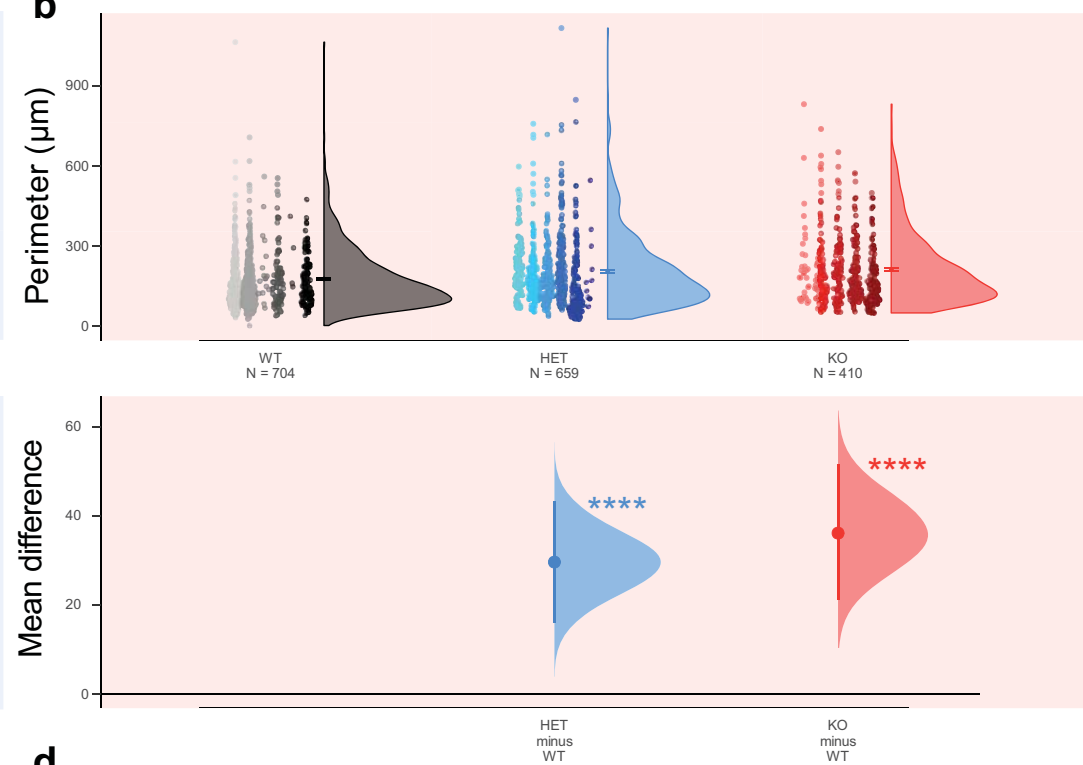**c**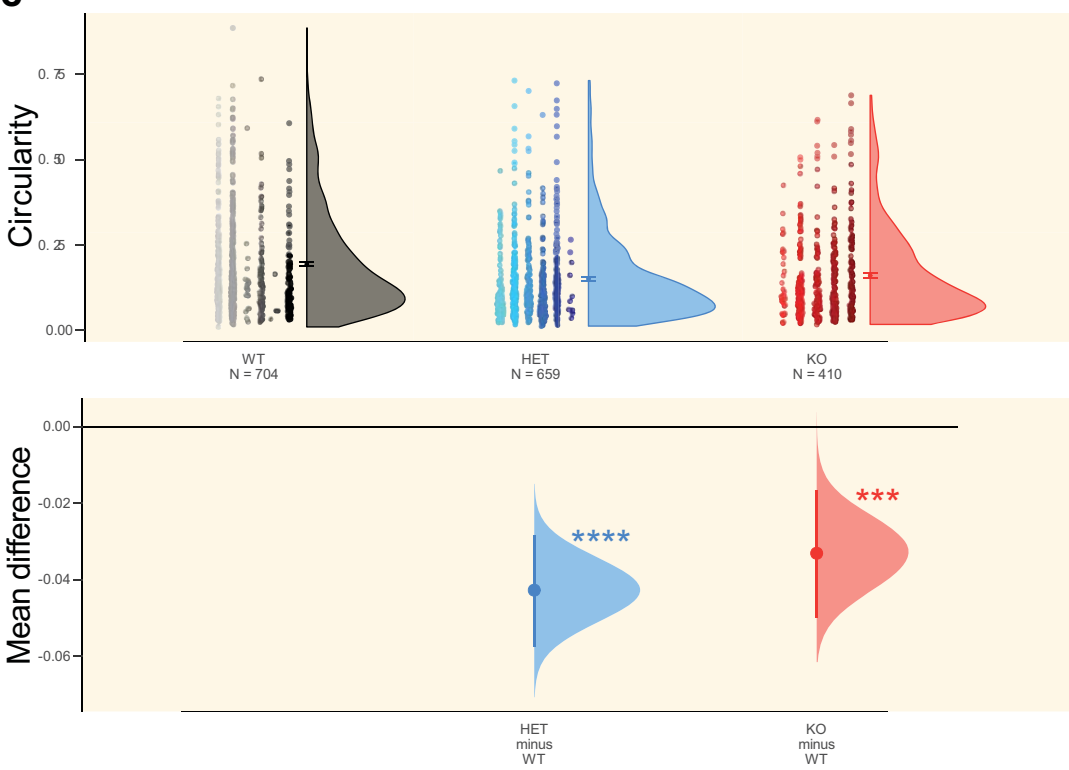**d**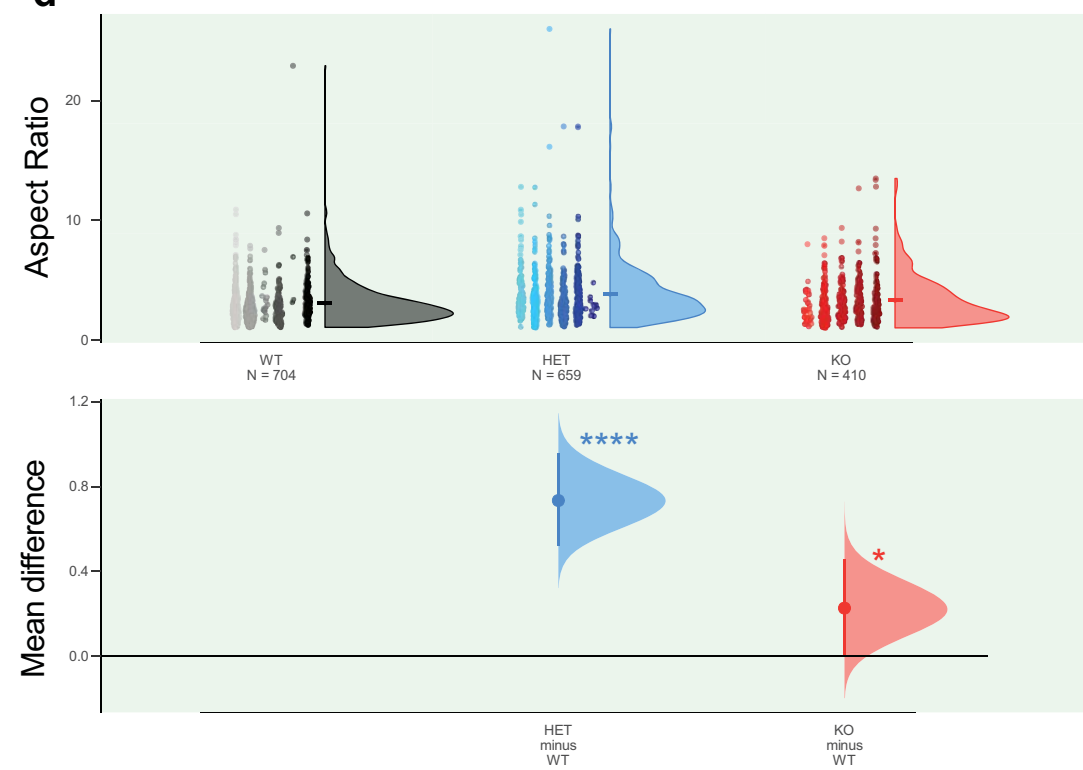

Supplement: Supplementary file 6 — Figure S6 [file 41380_2021_1179_MOESM6_ESM.pdf]

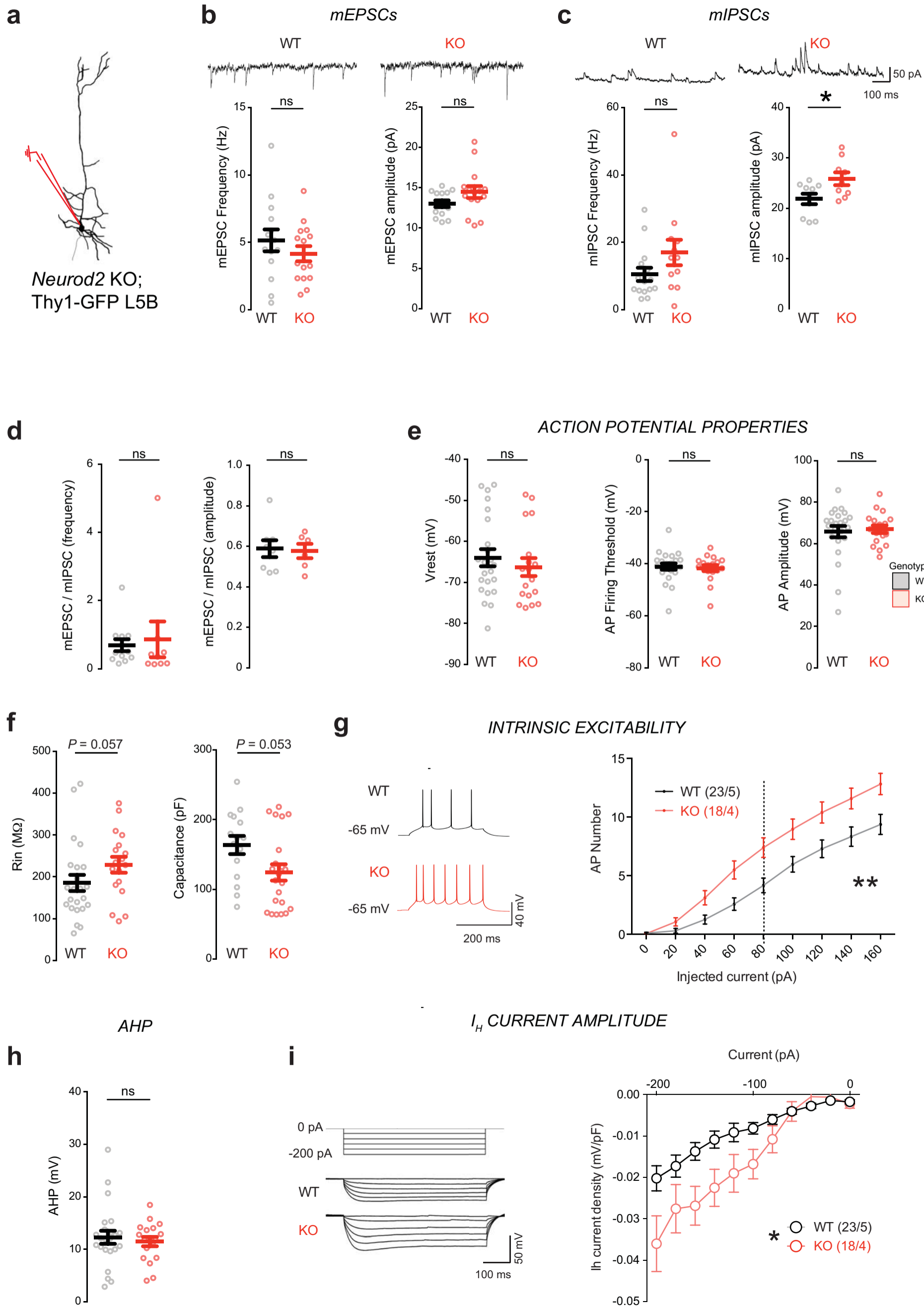

Supplement: Supplementary file 8 — Figure S8 [file 41380_2021_1179_MOESM8_ESM.pdf]

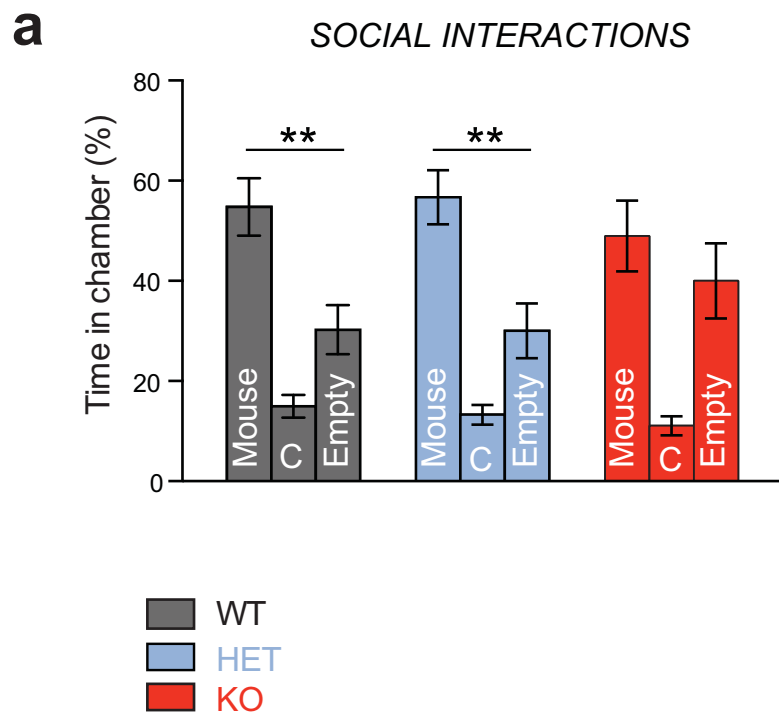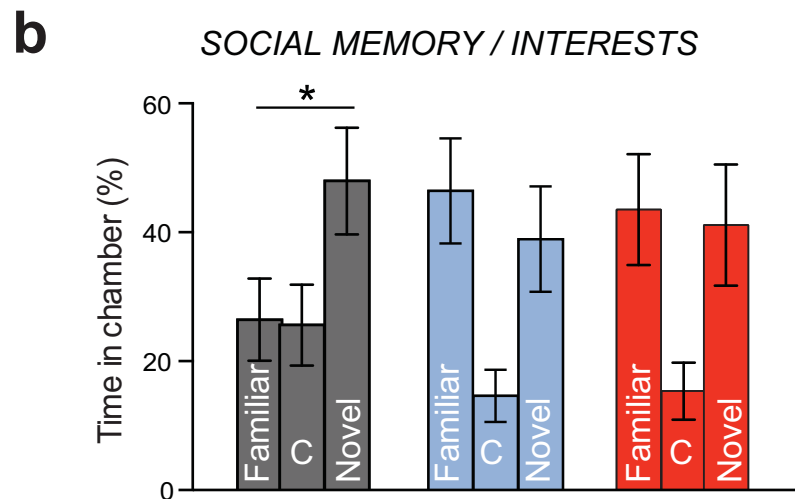

*OPEN FIELD - DETAILED BEHAVIOR OVER AN HOUR*

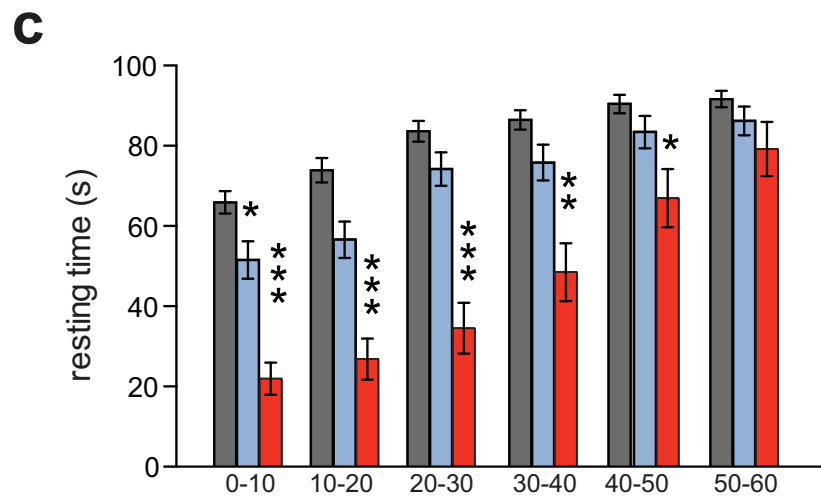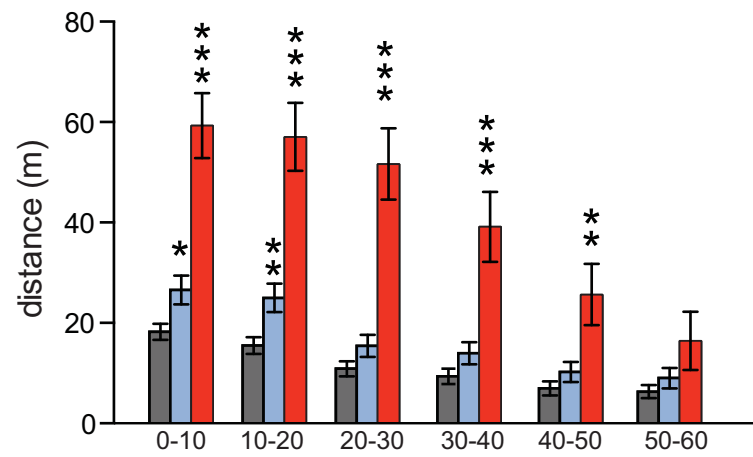

Supplement: Supplementary file 9 — Figure S9 [file 41380_2021_1179_MOESM9_ESM.pdf]

a

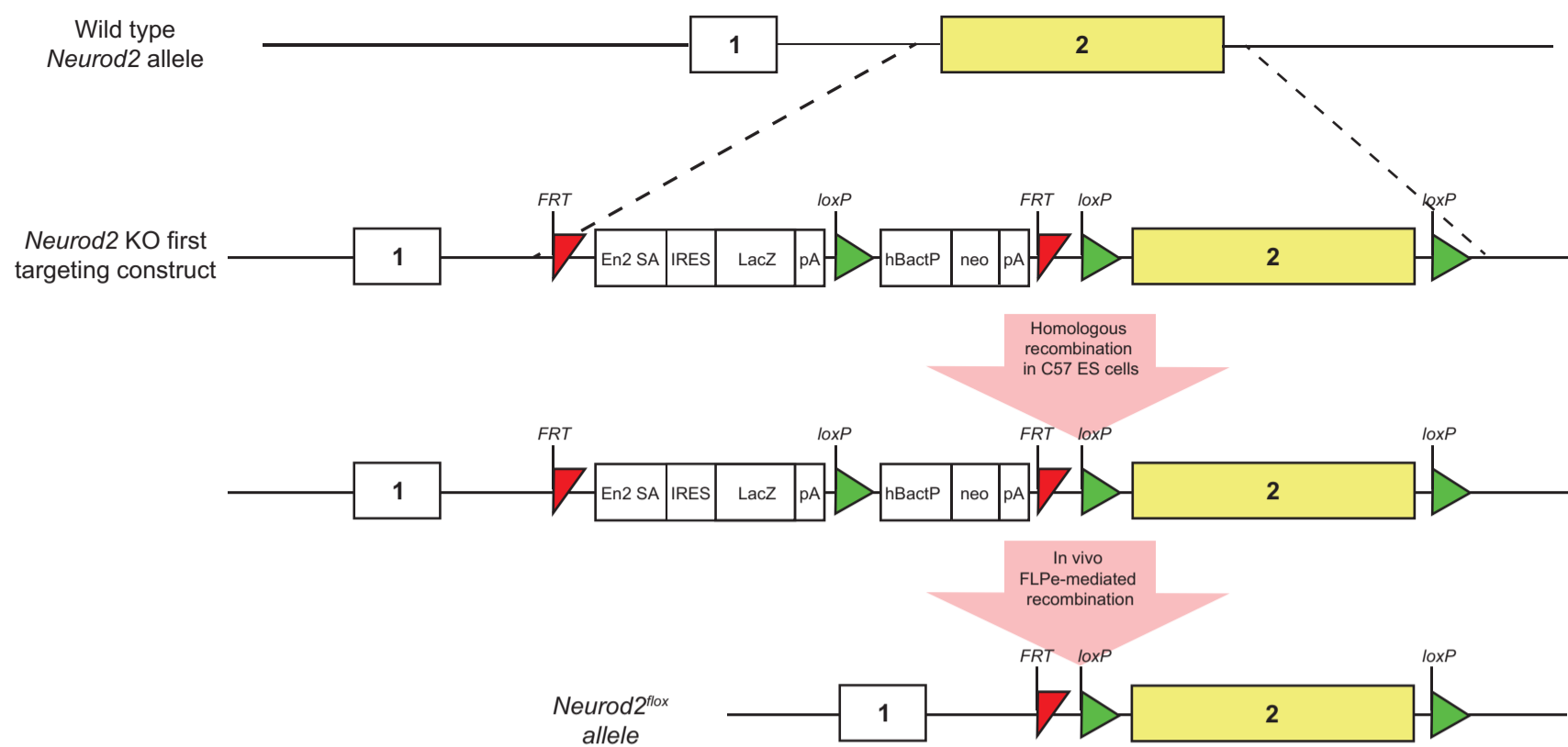

b

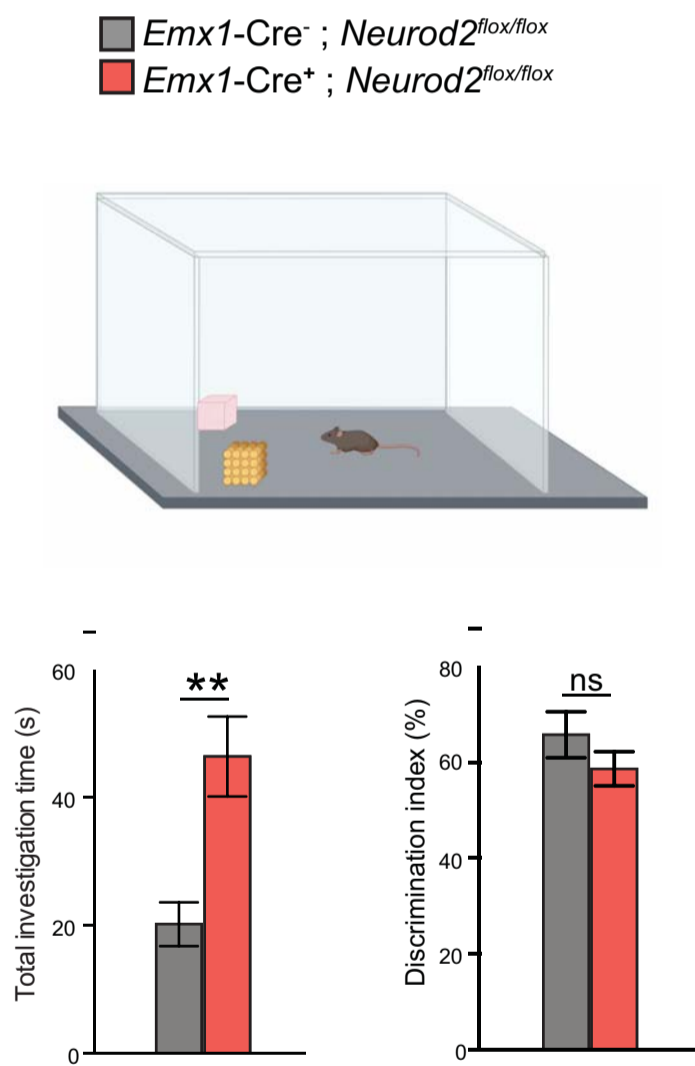

c

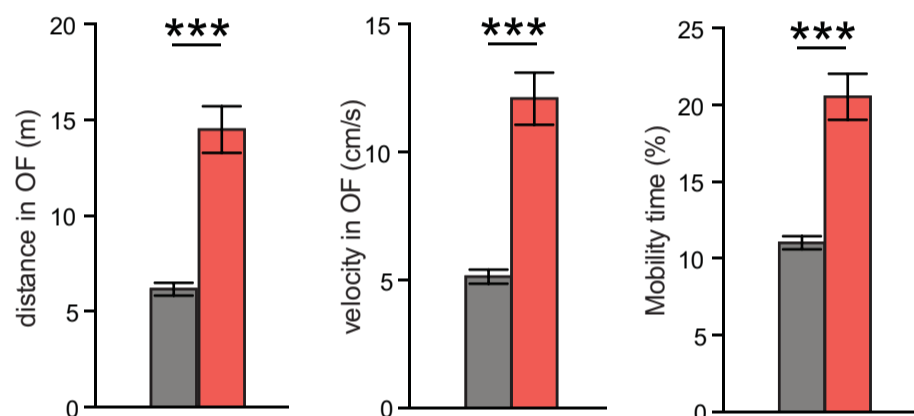

d

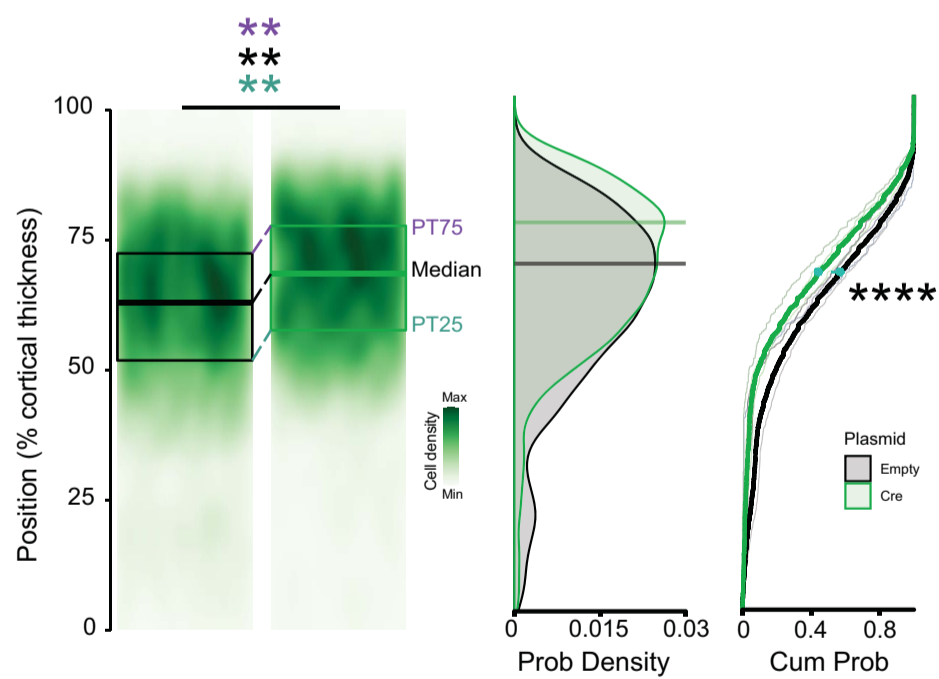

e

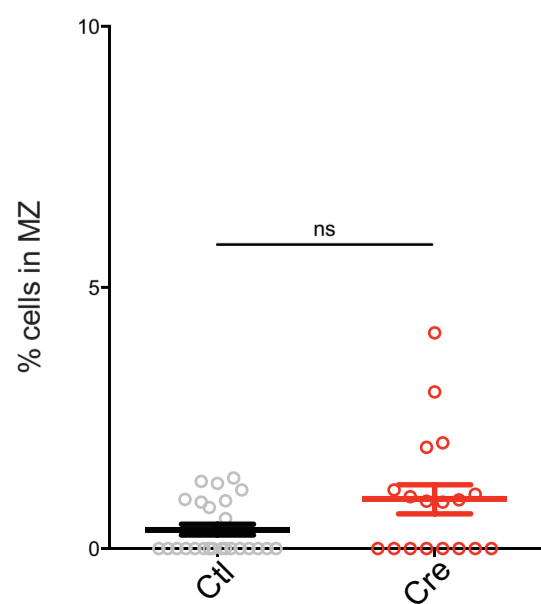

f

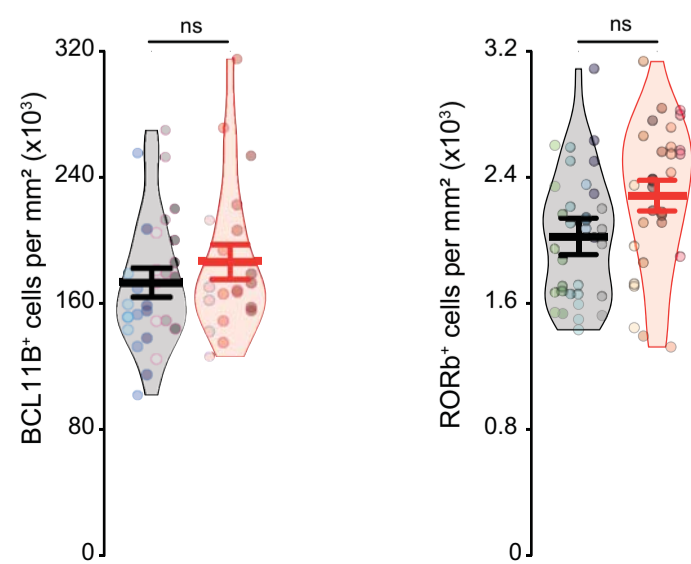

Supplement: Supplementary file 10 — Figure S10 [file 41380_2021_1179_MOESM10_ESM.pdf]

a

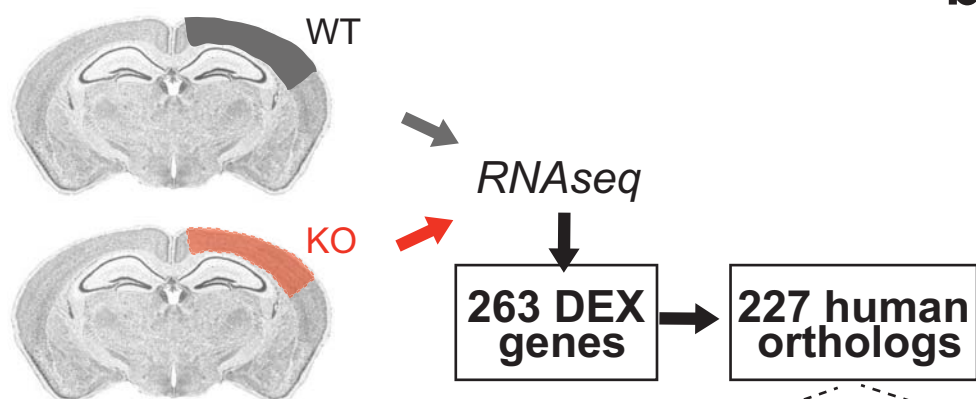

b

## Extensive Pumbed search

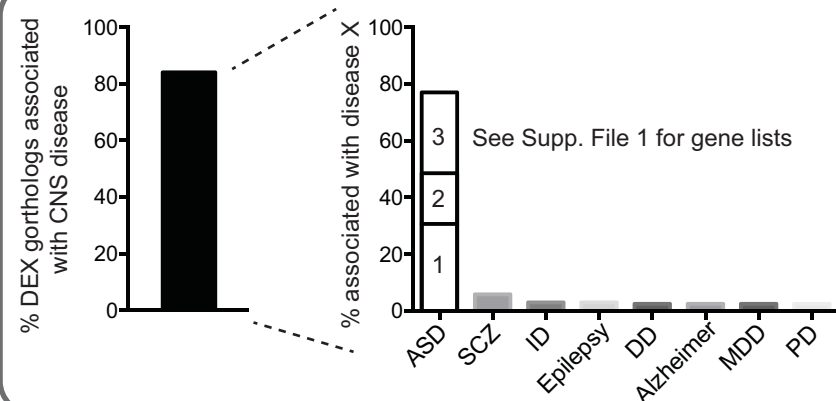

c

SCZ genes  
(OMIM «SCZ» and REF 32)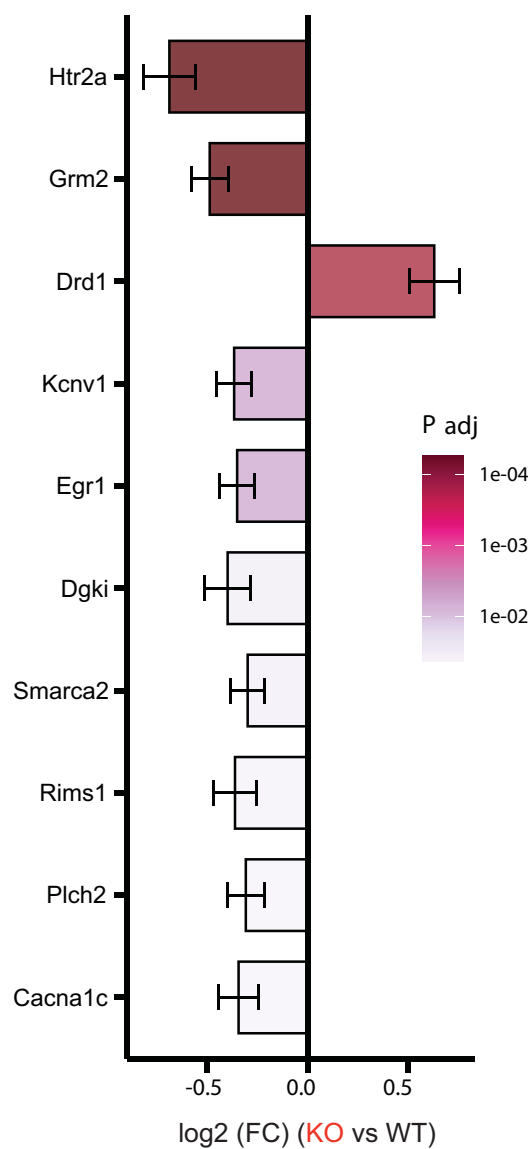FMRP genes  
(Darnell et al. 2011)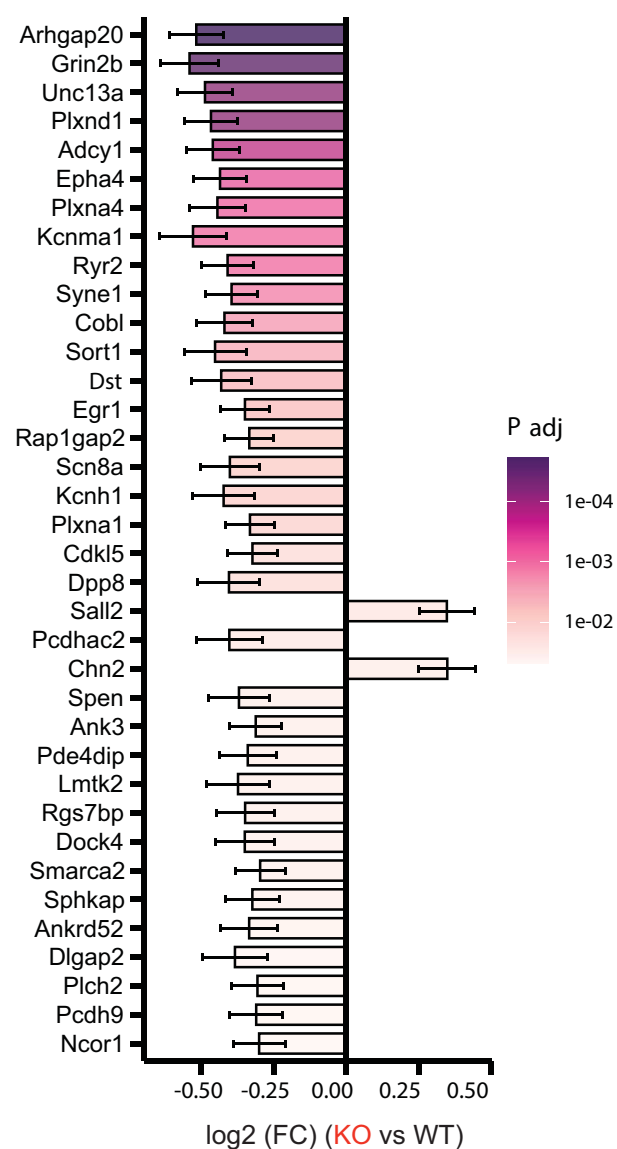

Supplement: Supplementary file 12 — Figure S12 [file 41380_2021_1179_MOESM12_ESM.pdf]

# S13

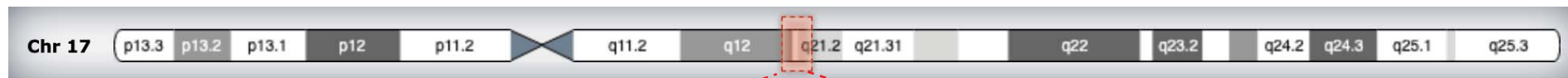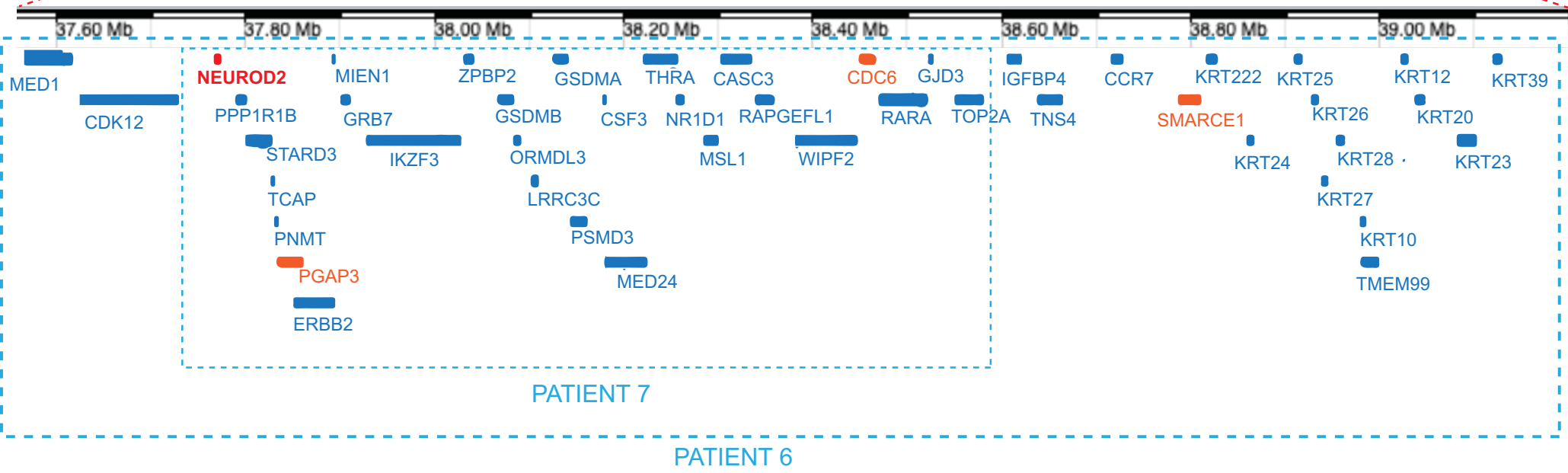

Supplement: Supplementary file 13 — Figure S13 [file 41380_2021_1179_MOESM13_ESM.pdf]
