# Supplementary material for: Disruption of NEUROD2 causes a neurodevelopmental syndrome with autistic features via cell-autonomous defects in forebrain glutamatergic neurons
Source: Mol Psychiatry. 2021 Jun 29;26(11):6125–48. doi: 10.1038/s41380-021-01179-x (PMC8760061; doi:10.1038/s41380-021-01179-x)
Supplement: Supplementary file 5 — Figure S5 [file 41380_2021_1179_MOESM5_ESM.pdf]

S5

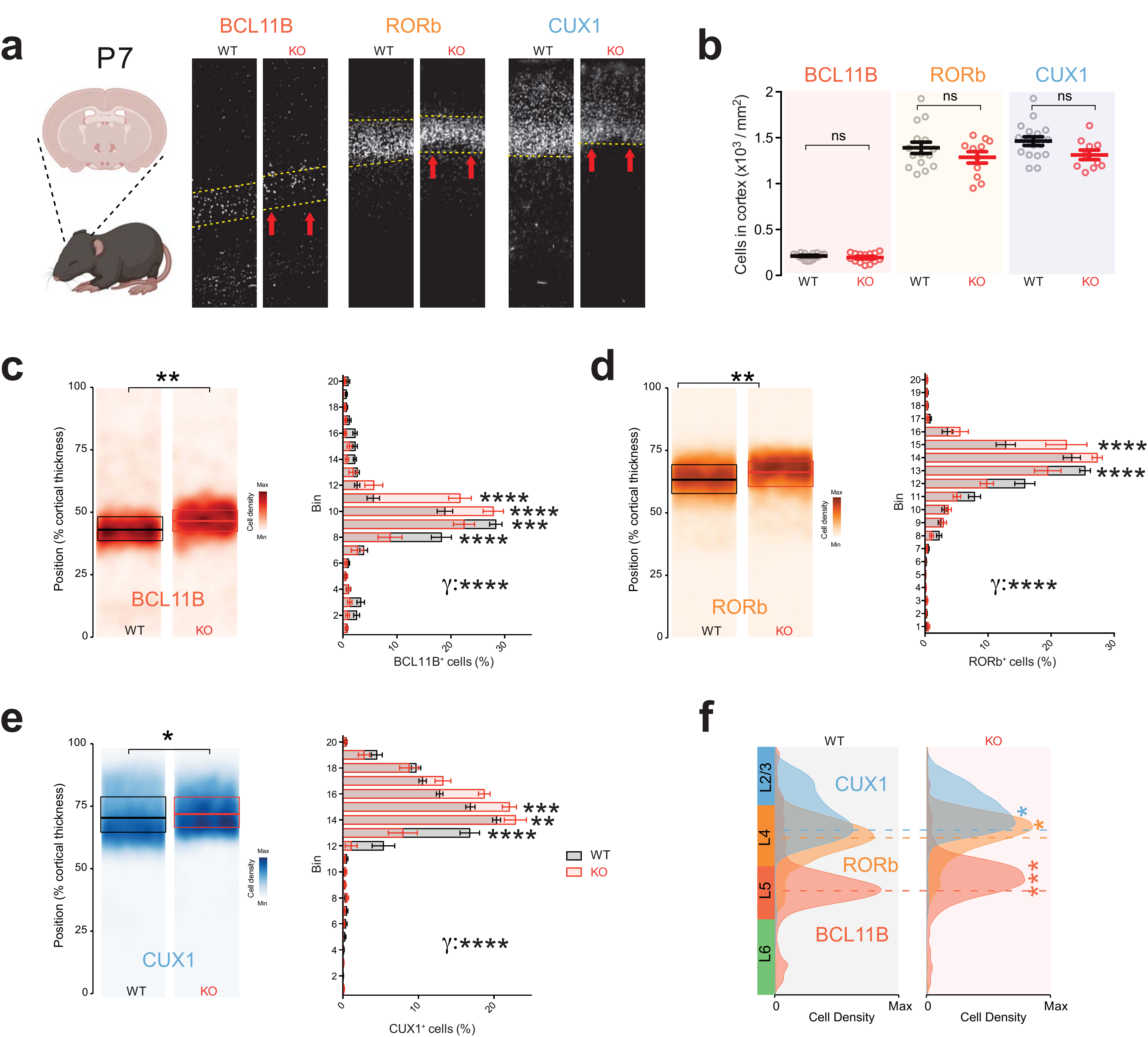

a
P7
BCL11B
RORb
CUX1
WT
KO
b
BCL11B
RORb
CUX1
ns
Cells in cortex (x10³ / mm²)
2
1.5
1
0.5
0
WT
KO
c
**
100
75
50
25
0
Position (% cortical thickness)
BCL11B
Cell density
Max
Min
Bin
BCL11B⁺ cells (%)
γ:****
d
**
RORb
RORb⁺ cells (%)
e
*
CUX1
CUX1⁺ cells (%)
f
WT
KO
L2/3
L4
L5
L6
0
Max
Cell Density
