# Supplementary material for: Disruption of NEUROD2 causes a neurodevelopmental syndrome with autistic features via cell-autonomous defects in forebrain glutamatergic neurons
Source: Mol Psychiatry. 2021 Jun 29;26(11):6125–48. doi: 10.1038/s41380-021-01179-x (PMC8760061; doi:10.1038/s41380-021-01179-x)
Supplement: Supplementary file 7 — Figure S7 [file 41380_2021_1179_MOESM7_ESM.pdf]

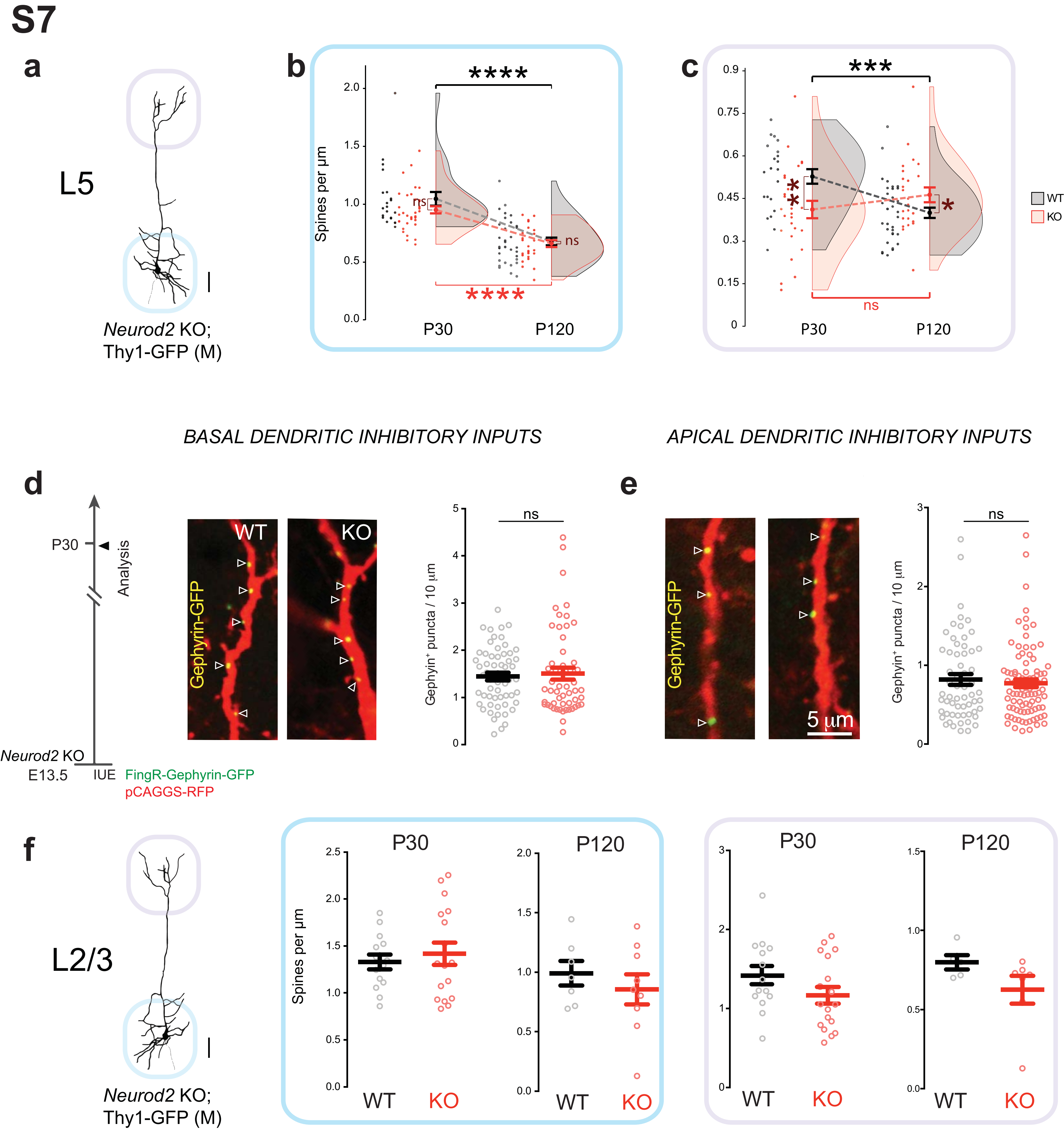

S7
a
L5
Neurod2 KO;
Thy1-GFP (M)
b
Spines per µm
****
ns
ns
****
P30
P120
c
***
ns
P30
P120
WT
KO
BASAL DENDRITIC INHIBITORY INPUTS
APICAL DENDRITIC INHIBITORY INPUTS
d
P30
Analysis
Neurod2 KO
E13.5
IUE
FingR-Gephyrin-GFP
pCAGGS-RFP
Gephyrin-GFP
WT
KO
Gephyin⁺ puncta / 10 µm
ns
e
Gephyrin-GFP
5 µm
Gephyin⁺ puncta / 10 µm
ns
f
L2/3
Neurod2 KO;
Thy1-GFP (M)
P30
P120
Spines per µm
WT
KO
WT
KO
P30
P120
WT
KO
WT
KO
