# Supplementary material for: Disruption of NEUROD2 causes a neurodevelopmental syndrome with autistic features via cell-autonomous defects in forebrain glutamatergic neurons
Source: Mol Psychiatry. 2021 Jun 29;26(11):6125–48. doi: 10.1038/s41380-021-01179-x (PMC8760061; doi:10.1038/s41380-021-01179-x)

S11

a

	Term	Count	*P* Value	Benjamini	FDR
GLOBAL	Voltage-gated ion channel activity	13	1.91E-06	0.002	0.003
GLOBAL	Signal transduction	30	7.34E-06	0.005	0.012
GLOBAL	Chemical synaptic transmission	11	3.06E-05	0.013	0.050
GLOBAL	Neuronal action potential	6	3.71E-05	0.012	0.061
GLOBAL	Intracellular signal transduction	18	5.25E-05	0.014	0.086
GLOBAL	Axon guidance	11	5.99E-05	0.013	0.098
GLOBAL	Regulation of membrane potential	8	2.13E-04	0.039	0.347
GLOBAL	Neuromuscular junction development	6	2.85E-04	0.045	0.464
GLOBAL	Adult walking behavior	6	3.29E-04	0.046	0.536
GLOBAL	Nervous system development	15	9.69E-04	0.118	1.570
GLOBAL	Long-term synaptic potentiation	6	9.99E-04	0.111	1.618
DOWN	Voltage-gated ion channel activity	11	4.27E-06	0.004	0.007
DOWN	Axon guidance	10	1.75E-05	0.008	0.027
DOWN	Neuronal action potential	5	1.40E-04	0.042	0.218
DOWN	Regulation of membrane potential	7	1.91E-04	0.043	0.298
DOWN	Ion transport	15	2.68E-04	0.048	0.419
DOWN	Nervous system development	13	3.19E-04	0.048	0.498
DOWN	Chemical synaptic transmission	8	4.53E-04	0.058	0.705
DOWN	Signal transduction	20	5.17E-04	0.058	0.805
UP	Regulation of cell growth	4	0.002	0.701	2.977
UP	Insulin secretion	3	0.006	0.851	9.085
UP	Signal transduction	10	0.010	0.858	13.619
UP	Cell adhesion	7	0.012	0.824	15.969
UP	Positive regulation of stress fiber assembly	3	0.016	0.848	20.979

b

GENE ONTOLOGY WITH CLUEGO

all differentially-expressed genes | *down-regulated in NeuroD2 KO*

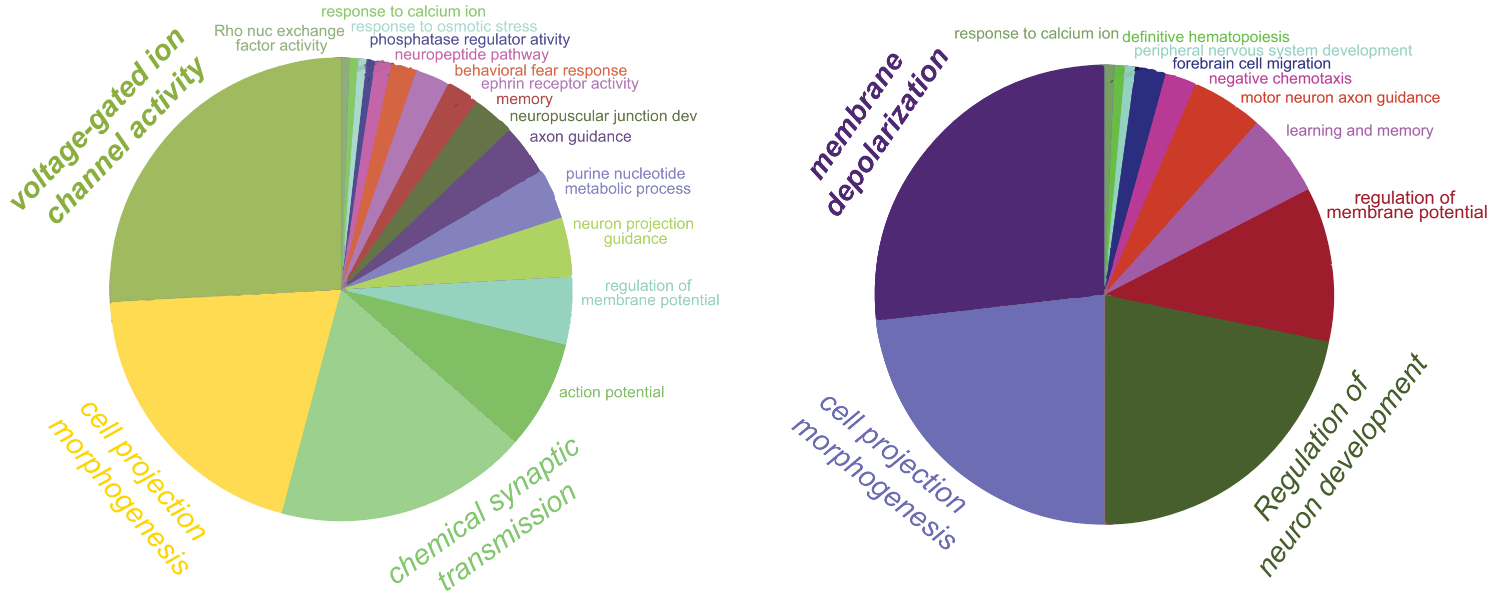

Supplement: Supplementary file 11 — Figure S11 [file 41380_2021_1179_MOESM11_ESM.pdf]
