# Supplementary material for: Disruption of NEUROD2 causes a neurodevelopmental syndrome with autistic features via cell-autonomous defects in forebrain glutamatergic neurons
Source: Mol Psychiatry. 2021 Jun 29;26(11):6125–48. doi: 10.1038/s41380-021-01179-x (PMC8760061; doi:10.1038/s41380-021-01179-x)
Supplement: Supplementary file 16 — Supplementary Legends [file 41380_2021_1179_MOESM16_ESM.docx]

**Figure S1: Expression of Neurod2 in neocortical cell subtypes across development (complement to Figure 1).** (a) qPCR shows permanent cortical expression of *Neurod2* mRNA with a peak of expression at E18.5. (b) NEUROD2 protein (green) is co-expressed with BCL11B (red) at P4 in L5. (c) At P28, NEUROD2 (red) never co-localizes with Gad67-GFP (green), but co-localizes with BCL11B (blue) (image in L5). Data are represented as means ± SEM. Statistical significance was evaluated by Kruskal-Wallis followed by Dunn’s post-hoc test with Bonferroni correction (a) (***P* < 0.01).

**Figure S2: Gross cortical anatomy and axonal projections are preserved in *Neurod2* KO mice**

(a-b) Callosal size and cortical size. Callosal width was slightly decreased (a) but cortical width was not significantly altered (b) in *Neurod2* KO mice. However, whole cortical area was decreased at P30 (right dot plot). Numbers of animals per condition are in parenthesis in the dot plots. (c,d) Axonal targeting specificity of cPN subtypes. Fluorescent retrograde cholera toxin beta injection in striatum, thalamus (c) and contralateral M1 L2-5 (d) led to unaltered distribution of retrogradely-labeled somata in ipsilateral motor/somatosensory cortex. N=4 WT and KO mice, 5 slices per mouse were counted. Data are represented as means ± SEM. Statistical significance was evaluated by Student t-test or Mann-Whitney test depending on normality of samples [(a) and (b)] (**P* < 0.05).

**Figure S3: Proliferation and apoptosis at E18.5, P7 and P30**

(a) KI67 images and cell quantifications in WT versus KO mice. KI67^+^ cells were counted in entire S1 cortical columns ranging from VZ to pia at E18.5, and in the V/SVZ at P7 and P30. Quantifications with violin plots show no difference between WT and KO. (b) PH3 images and cell quantifications in WT versus KO mice. PH3^+^ cells were counted in the V/SVZ at all ages. (c) Activated capsase-3 cell quantifications in WT versus KO mice. Activated caspase-3^+^ cells were counted in the V/SVZ, the whole cortex or the whole S1 cortex separately. N=10 mice per condition, circles in violin plots represent individual mice. Data are represented as means ± SEM. Statistical significance was evaluated by Student t-test or Mann-Whitney test depending on normality of samples [(a) and (b)].

**Figure S4: Altered radial migration in *Neurod2* KO mice (complement to Figure 2)**

(a) Gene ontology on NEUROD2 Chip-seq target genes in the E14.5 cortex (data from Bayam et al., 2015). Displayed genes are genes that are at least encompassed in 3 of the 5 GO terms. (b) Excess migration in *Neurod2* KO and HET mice. Left, mean cell laminar position in percentage of the cortical thickness for WT, *Neurod2* KO and HET mice. Middle, cumulative probability distribution of cells in the laminar axis. Right, bin-based laminar distribution. We analyzed 21 slices from 4 WT, 20 slices from 5 HET and 26 slices from 4 KO mice. (c) Diminished migration of L5 cPNs when human *NEUROD2* is overexpressed post-mitotically. Left, mean cell laminar position in percentage of the cortical thickness for pND1-GFP and pND1-*NEUROD2*-GFP plasmid electroporations. Middle, cumulative probability distribution of cells in the laminar axis. Right, probability density function showing the average laminar distribution of cells. We analyzed 21 slices from 3 pND1-GFP and 65 slices from 9 pND1-*NEUROD2*-GFP mice. (d-e) Images and percentage calculations of RFP-electroporated cells in MZ from E13.5-E15.5 experiments. (e) The percentage of electroporated cells in MZ was unaffected by genotypes. (f) MZ thickness at E18.5, L1 thickness at P7 and P30. For (d-f), we analyzed 21 slices from 4 WT, 19 slices from 5 HET and 26 slices from 3 KO mice. (g) Mean number of neurites per electroporated cell in IZ for WT HET and KO mice. We analyzed 30 slices from 6 WT, 30 slices from 5 HET and 19 slices from 5 KO mice. Data are represented as means ± SEM except in mean cell position plots (b-c) where median, first and third quartiles are represented. Statistical significance was evaluated by permutation test for medians [(b-c)], Anderson-Darling test [cumulative probabilities and probability densities in (b-c)] or by Permutation test for a spatially adjusted two-way ANOVA followed by Bonferroni’s post-hoc test [bin graphs in (b)] (**P* < 0.05, ***P* < 0.01, ****P* < 0.001, *****P* < 0.0001).

**Figure S5: Altered laminar position of cPN types at P7 in *Neurod2* KO mice (complement to Figure 2)**

(**a**) Representative confocal photomicrographs of S1 cortical columns at the somatosensory level for BCL11B, RORb and CUX1 layer markers. (**b**) Number of cPN subtypes in a S1 cortical column, labeled using for the four-layer markers; note the absence of significant difference between genotypes. (**c**-**f**) Left, mean cell laminar position in percentage of the cortical thickness. Right, twenty-bin based laminar distribution of cPN subtypes in WT (grey bars) and KO mice (red bars). We used the markers BCL11B for L5 (**c**), RORβ for L4 (**d**) and CUX1 for L2-4 (**e**). We analyzed 25 to 42 S1 cortical columns from 9/6/9/7 WT (for TBRA, BCL11B, RORb and CUX1) and 5 KO mice. (**f**) Probability density function showing the average laminar distribution of cells. All layers were switched superficially. Data are represented as means ± SEM except in mean cell position plots (**c**-**f**) where median, first and third quartiles are represented. Statistical significance was evaluated by permutation test for medians [(c), (d), (e) left], Student t-test or Mann-Whitney test depending on normality of samples [(b)], Permutation test for a spatially adjusted two-way ANOVA followed by Bonferroni’s post-hoc test [bin graphs in (c-e)] or by Anderson-Darling test [probability densities in (f)] (**P* < 0.05, ***P* < 0.01, ****P* < 0.001, *****P* < 0.0001).

**Figure S6: Morphological changes in IZ cells of *Neurod2* HET and KO mice (complement to Figure 2)**

Cumming estimation plot for cell area (a), perimeter (b), circularity (c) and aspect ratio (d) (ref Georg Cumming 2012, “Understanding The New Statistics”). For each parameter, dot and violin plot visualizations along with summary measurements (mean +/- sem) of raw data are shown on top panels. On bottom panels, mean differences are plotted as bootstrap sampling distributions. Each mean difference is depicted as a dot. Each 95% confidence interval is indicated by the ends of the vertical error bars. P values of the two-sided permutation t-test are shown.

**Fig. S7: Evolution of spine density in *Neurod2* KO mice (complement to Figure 3)**

(**a**) Imaris reconstruction of a L5 cPN, basal and apical compartments are underscored by a blue and purple halo, respectively. (**b**,**c**) Spine density at P30 and P120 in basal (**b**) and apical (**c**) compartments. (d,e) Inhibitory input puncta. GEPHYRIN-GFP intrabodies and RFP were co-electroporated at E13.5 and the number of GEPHYRIN-GFP+ puncta per unit of dendrite length in RFP+ cells measured in basal (d) and apical (e) compartments. (f) Spine density in L2/3 neurons. AAV1.hSyn.TurboRFP.WPRE.RBG was slowly injected in L2/3 of M1 and spine density analysis performed on brains collected 7 days later. Data are represented as means ± SEM. Statistical analyses were performed using two-tailed t-tests or Mann-Whitney test depending on the normality of samples (**P* < 0.05, ***P* < 0.01, ****P* < 0.001, *****P* < 0.0001).

**Figure S8: Electrophysiological properties of L5 cPNs in Neurod2 KO mice**

(**a-c**) Miniature post-synaptic currents. (**a**) Patch clamp recording of Thy1-GFP cPNs in L5B. (**b**) Representative traces, frequency and amplitude of mEPSCs (each circle is a recorded cell with a mouse color code in the violin plots). (**c**) Example traces, frequency and amplitude of mIPSCs. Amplitude of mIPSC was significantly increased. (**d**) ﻿The ratio of mEPSC to mIPSC frequency and amplitude was unchanged in *Neurod2* mutant neurons compared to WT. (**e**) Action potential properties, including Vrest, AP firing threshold and AP amplitude. (**f**) Membrane resistance and capacitance. (**g**) Increased intrinsic excitability. Left, representative firing responses to +80 pA current steps in a WT (black) and a KO (red) cell. Right, *Neurod2* KO neurons reached action potential (AP) firing threshold earlier than WT neurons and exhibited a steeper input-output relationship, as assessed by the number of APs elicited by increasing current injections (from +20 to +160 pA, 20-pA increments) during current-clamp recordings. (**h**) After-hyperpolarization (AHP) was normal in L5 cPNs of *Neurod2* KO mice. (**i**) *Neurod2* KO L5 cPNs exhibited increased Ih-current amplitudes compared with WT PNs (current/voltage relation of Ih currents). Left, experimental protocol and representative traces; right, summary graph of the voltage-current relation. N≥4 mice per genotype, circles in dot plots represent cells. Data are represented as means ± SEM. Statistical analyses were performed using two-tailed t-tests or Mann-Whitney test depending on the normality of samples [(b-f) and (h)], by two-way ANOVA followed by Bonferroni’s post hoc test [(b), (c)] and by two-way repeated measure ANOVA followed by Bonferroni’s post hoc test [(g), (i)]. **P* < 0.05, ***P* < 0.01.

**Figure S9: Behavioral phenotypes in *Neurod2* KO and HET mice (complement to Figure 4)**

(a,b) Time spent in the different chambers during the 10 minutes social interaction (a) and social memory (b) tests. (c) Resting time and distance during 10 minutes intervals over the 1-hour open field assessment. Data are means ± SEM. Statistical significance was evaluated by two-way Mixed ANOVA followed by post hoc analysis using paired t-test [(a) to (c)] (ns, not significant; **P* < 0.05; ***P* < 0.01; ****P* < 0.001).

**Figure S10: Forebrain excitatory neuron-specific *Neurod2* deletion recapitulates ASD-like phenotypes in mice (complement to Figure 5)**

(a) Scheme of the strategy used to generate C57BL/6 mice carrying the Neurod2 FLOX allele. 1 and 2 are exons, exon 2 is the only coding exon. (b) Emx1^IRES^*^Cre^* ; *Neurod2*^flox/flox^ mice displayed increased total investigation time and discriminated normally between a familiar and a novel object. (c) Hyperactivity in Emx1^IRES^*^Cre^* ; *Neurod2*^flox/flox^ mice. Left graph depicts the distance traveled in 20 minutes in the open field. Middle graph shows velocity during motion and right graph shows the percentage of time during which the mouse is mobile. We analyzed 10 mice aged 8-14 weeks depending on the test. (d) Excess migration after acute deletion using Cre/flox strategy. Left, mean cell laminar position in percentage of the cortical thickness. Middle, Probability density function showing the average laminar distribution of cells. Right, empirical cumulative distribution function. (e) Density of BCL11B and RORb-expressing cells in Emx1^IRES^*^Cre^* ^+^ ; *Neurod2*^flox/flox^ *vs* control Emx1^IRES^*^Cre^* ^-^ ; *Neurod2*^flox/flox^ littermates per S1 cortical column, represented with a violin plot. For BCL11B, we analyzed 20 slices from 4 Emx1^IRES^*^Cre^* ^-^ ; *Neurod2*^flox/flox^ mice and 30 slices from Emx1^IRES^*^Cre^* ^+^ ; *Neurod2*^flox/flox^ mice. For RORb, we analyzed 35 slices from 7 *Emx1*-Cre^-^ ; *Neurod2*^flox/flox^ mice and 30 slices from Emx1^IRES^*^Cre^* ^+^ ; *Neurod2*^flox/flox^ mice. Data are means ± SEM. Statistical significance was evaluated by two-tailed t-tests or Mann-Whitney test depending on the normality of samples [(b), (c), (e)], permutation test for medians (d), and by Anderson-Darling test [probability densities and cumulative probability in (d)] (ns, not significant; **P* < 0.05; ***P* < 0.01; ****P* < 0.001).

**Figure S11: Gene ontology (complement to Figure 6)**

(a) Gene ontology Table of DEX genes in M1/S1 of *Neurod2* KO mice with DAVID (<https://david.ncifcrf.gov/>). GLOBAL: all DEX genes. DOWN: down-regulated genes. UP: up-regulated genes. FDR: False Discovery Rate. (b) Gene ontology pie charts from ClueGO (Cytoscape).

**Figure S12: *Neurod2* KO DEX genes associated with neuropsychiatric disorders (complement to Figure 6)**

(a) From mixed motor and somatosensory cortical bulk tissue, there were 263 DEX genes in KO mice, 227 of which had human orthologs. (b) ﻿Human brain and nervous system pathologies associated with orthologs of the Neurod2-regulated DEX genes. Scores: 1, one study; 2, two studies; 3, three or more studies. DD, Developmental Delay; ID, intellectual disability; MDD, major depressive disorder; PD, Parkinson’s disease. (c) Fold change expression (FC; log2 scale) of Neurod2 KO DEX genes belonging to specific gene families, ranked according to adjusted *P* value. In the graph representing synaptome genes (SYN), presynaptic genes are underscored in light blue while all other genes are postsynaptic.

**Figure S13: Schematic representation of 17q12 deletions in patients 6 and 7 (complement to Figure 7).**

In orange, genes associated with CNS disease. See File S1, sheet “Genelist_ 2 deletion patients”, for more infos on these genes.

**Video S1: Spontaneous epileptic seizures in *Neurod2* KO and cKO mice (complement to Figure 4)**

Behavioral seizures were observed in 6/18 *Neurod2* KO, 2/22 HET and 1/10 cKO mice during the course of the behavioral experiments (5 KO and 1 HET during open field test, 1 KO, 1 HET and 1 cKO during the 3-chamber test), and were followed by sudden death in 3 KO mice. Behavioral seizures were captured on video in 2 *Neurod2* KO mice and the cKO mouse. In KO mouse #215-106, seizure manifested as a sudden stop of exploratory behavior and brief myoclonic jerks (1-2 sec), immediately followed by a myoclonic seizure (10sec) with total loss of quadruped posture. During myoclonic seizure, mouse #215-106 exhibited repeated clonic jerks of the limbs while lying on its side in a curled-up position. Episode stopped after ~10sec when the mouse recovered postural control, and was followed by 1-2min of hypoactivity. In KO mouse #215-088, seizure started with neck jerks, followed by a brief period (6-7sec) with rearing and forelimb clonus. Seizure behavior quickly progressed to a high intensity stage of wild jumps that stopped after ~25sec, and the mouse remained immobile/unresponsive for ~1min. In the Emx1^IRES^*^Cre^* ; NeuroD2^flox/flox^ mouse, the behavioral seizure started with a sudden rearing on hindlimbs, unilateral hindlimb jerks and falling, followed by brief myoclonic movements of the limbs while lying on its side. Episode rapidly progressed to a brief stage of wild jumps (~3sec) followed by a loss of quadrupedal posture and tonic-clonic seizures. The later started with tonic contractions resulting in a hunchback posture followed with sustained clonus of the limbs occasionally interrupted by phases of tonic extension of the body. The mouse recovered after ~15sec and went back to exploratory behavior while manifesting hyperactivity and circling movements.

**File S1:** NEUROD2 and Neurodevelopmental disorders.

This File contains 7 sheets:

- 263 Neurod2 DEX genes_Fig1: list of DEX genes, related to Fig. 1

- Orthologs_Disease(PubMed)_Fig6: Human paralogs of DEX genes: disease association (Pubmed analysis), related to Fig. 6

- Orthologs_NND_asso_Fig6: Overlap of human paralogs of DEX genes with published NDD gene lists, related to Fig. 6

- GWAS_NEUROD2_GeneModule_Fig6: GWAS datasets used for disease association in gene module 37, related to Fig. 6

- Genelist_DeletionPatients_Fig7: Map of chromosome 17 showing genes in the two patients with short deletion, and association with CNS/CNS disorders according to OMIM for each of these genes, related to Fig. 7

- NEUROD2_PathoPredict_Fig7: Pathogenicity indicators for patients’ variants, related to Fig. 7

- egoE14.5_ChipSeq_neuroMigr_Disc: Gene lists of migration-related GOs for E14.5 Chip-seq study [ref 20]. For each gene in GO “cerebral cortex radially oriented cell migration” (GO:0021799), we added a Pubmed-based search for radial migration processes altered, related to Discussion.
